# Extracellular matrix assembly stress initiates *Drosophila* central nervous system morphogenesis

**DOI:** 10.1016/j.devcel.2023.03.019

**Published:** 2023-05-22

**Authors:** Eduardo Serna-Morales, Besaiz J. Sánchez-Sánchez, Stefania Marcotti, Angus Nichols, Anushka Bhargava, Anca Dragu, Liisa M. Hirvonen, María-del-Carmen Díaz-de-la-Loza, Matyas Mink, Susan Cox, Emily Rayfield, Rachel M. Lee, Chad M. Hobson, Teng-Leong Chew, Brian M. Stramer

**Affiliations:** 1 Randall Centre for Cell and Molecular Biophysics, King’s College London, SE1 1UL London, UK; 2 Institute of Medical Biology, University of Szeged, 6720 Szeged, Hungary; 3 School of Earth Sciences, University of Bristol, BS8 1QU Bristol, UK; 4 Advanced Imaging Center, Howard Hughes Medical Institute Janelia Research Campus, Ashburn, VA 20147, USA

**Keywords:** *Drosophila*, basement membrane, collagen IV, morphogenesis, central nervous system, extracellular matix, surface tension, embryonic development

## Abstract

Forces controlling tissue morphogenesis are attributed to cellular-driven activities, and any role for extracellular matrix (ECM) is assumed to be passive. However, all polymer networks, including ECM, can develop autonomous stresses during their assembly. Here, we examine the morphogenetic function of an ECM before reaching homeostatic equilibrium by analyzing *de novo* ECM assembly during *Drosophila* ventral nerve cord (VNC) condensation. Asymmetric VNC shortening and a rapid decrease in surface area correlate with the exponential assembly of collagen IV (Col4) surrounding the tissue. Concomitantly, a transient developmentally induced Col4 gradient leads to coherent long-range flow of ECM, which equilibrates the Col4 network. Finite element analysis and perturbation of Col4 network formation through the generation of dominant Col4 mutations that affect assembly reveal that VNC morphodynamics is partially driven by a sudden increase in ECM-driven surface tension. These data suggest that ECM assembly stress and associated network instabilities can actively participate in tissue morphogenesis.

## Highlights

• Sudden uneven Col4 assembly surrounding the fly VNC initiates tissue condensation
• Col4 flows coherently on the tissue surface independently of cellular contractility
• Modeling indicates that a BM-dependent increase in surface tension drives morphogenesis
• Perturbations of Col4 assembly affect the rate of VNC condensation

## Graphical abstract

**Figure:**
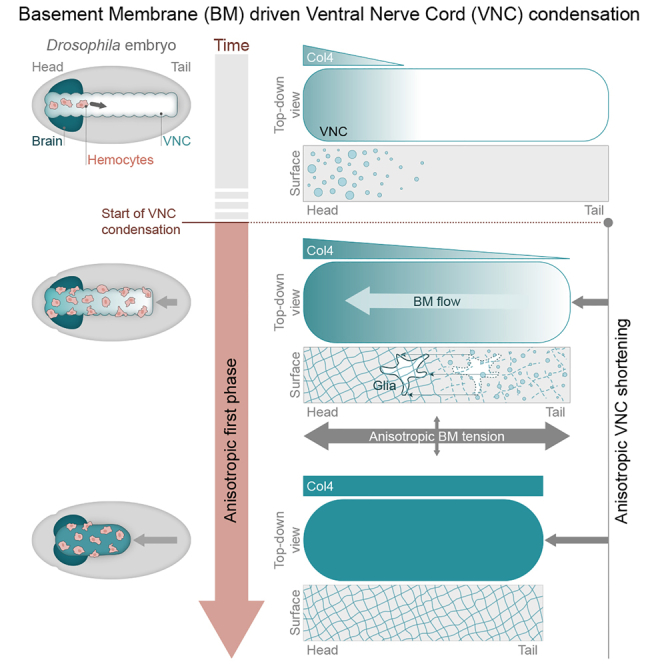

## Teaser

Serna-Morales et al. demonstrate that the initiation of *Drosophila* VNC condensation is triggered by sudden basement membrane assembly around the tissue, which increases the surface tension independently of cellular contractile activity. Their work suggests that intrinsic stresses in an assembling basement membrane network can actively contribute forces that drive tissue morphogenesis.

## Introduction

The forces controlling tissue development are attributed to cellular-driven activities.^1^ Any role for extracellular matrix (ECM) in modulating overall tissue shape is largely assumed to be passive by providing a substrate that allows cells to actively remodel their environment.^2^ However, recent work on developing zebrafish semicircular canals has revealed that ECM accumulation can have a more instructive role in shaping tissues through swelling and modulation of osmotic pressure.^3^ In addition, all polymer networks—including ECM—can theoretically develop autonomous stresses during their initial polymerization, and there has been speculation that out-of-equilibrium ECM behaviors may be playing a more active role during tissue morphogenesis than many would assume.^4^^,^^5^^,^^6^^,^^7^ Investigating these active mechanisms requires analyzing the ECM network during embryonic assembly before reaching a crosslinked, homeostatic state. Here, we exploit *Drosophila* embryogenesis, which involves a *de novo* burst of ECM polymerization midway through development,^8^^,^^9^ to examine the role of an out-of-equilibrium ECM network during central nervous system development. Although altering cellular activity failed to stop the initiation of ventral nerve cord (VNC) morphogenesis, we found that perturbing ECM assembly or delivery to the tissue surface severely affected the process. Live imaging of ECM polymerization during morphogenetic initiation identified a sudden coherent flow of matrix on the tissue surface—independent of cellular dynamics—in a posterior to anterior direction, which correlates with the overall motion of the tissue. In addition, finite element modeling identified that an anisotropic surface tension along the axis of ECM motion was sufficient to explain the complex shape changes associated with VNC morphogenesis. These findings show that ECM assembly can actively contribute forces that shape a developing tissue.

## Results

### *Drosophila* VNC condensation consists of two distinct temporal phases, and initial anisotropic changes in tissue morphology are independent of VNC cellular activity

**Figure 1:**
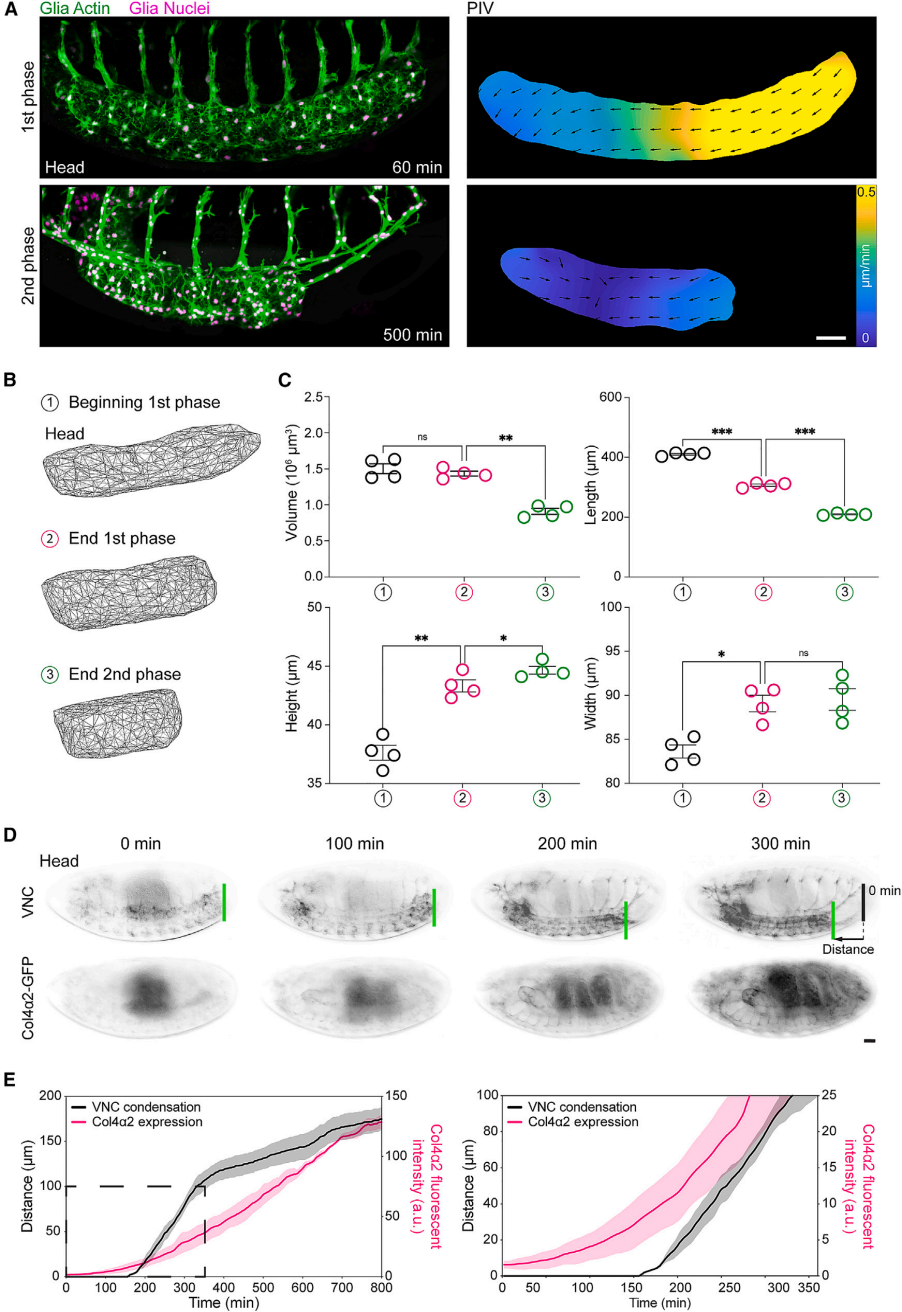
VNC morphogenesis involves distinct stages and correlates with the initiation of Col4 assembly (A) Live imaging of VNC morphogenesis (left panels) and quantification of tissue motion by PIV (right panels) revealing anisotropic (1^st^ phase) and isotropic (2^nd^ phase) phases of condensation. Scale bar, 30 μm. (B) 3D reconstruction of VNC shape during the phases of condensation. (C) Quantification of VNC shape at the start (1), end of the 1^st^ phase (2) and end of the 2^nd^ phase (3) of condensation. Repeated measures one-way ANOVA with Geisser-Greenhouse correction and Holm-Šídák’s multiple comparisons tests. Each dot represents one embryo, n= 4 embryos. Error bars show standard error of the mean. Volume: ns p = 0.4130, ∗∗p = 0.0073; Length: ∗∗∗p = 0.0004; Height: ∗∗p = 0.0045, ∗p = 0.0155; Width: ∗p = 0.0278, ns p = 0.6803. (D) Live imaging of VNC condensation (highlighted by the difference between the green and black lines) and induction of Col4 production by quantifying fluorescence intensity of Col4α2-GFP. Scale bar, 30 μm. (E) Correlation of Col4 fluorescence intensity with the rate of VNC condensation as measured by tracking the motion of the tail of the tissue. Right panel focuses on the data within the dashed square. n = 3 embryos. See also Video S1.

During stage 15 of *Drosophila* development, the embryonic nerve cord undergoes a sudden reduction in length in a process called VNC condensation. Time-lapse imaging of the entire ∼12 h of condensation revealed two distinct phases in VNC morphodynamics: a rapid 1^st^ phase when most of the morphological remodeling occurs in which the tissue shortens asymmetrically from tail to head over ∼3 h and a slower 2^nd^ phase resulting in a symmetric reduction in length over the remaining ∼9 h of development (Figures 1A, S1A, and S1B; Video S1, parts 1–3). Analyses of tissue geometry revealed that although the 2^nd^ phase involved a reduction in tissue volume, the change in shape during the 1^st^ phase was isovolumetric, suggesting that at least for the 1^st^ phase, the process is not a simple “condensation” (Figures 1B and 1C). Interestingly, it was recently reported that the scaling of VNC length with embryo length is suddenly lost after the start of condensation,^10^ which is likely explained by the increase in thickness of the tissue (Figures 1B and 1C). These data suggest that VNC morphogenesis involves temporally controlled and mechanistically distinct processes.

Video S1. VNC condensation involves two distinct phases and the initiation of VNC morphogenesis correlates with hemocyte migration and Col4 deposition, related to Figures 1 and S1 Part 1. Lattice light-sheet imaging of VNC condensation. Time-lapse imaging of the full thickness of the VNC by lattice light-sheet microscopy in embryos containing glia labelled for actin. The top and bottom panels are the lateral and ventral view of the tissue, respectively. For visualisation purposes, the intensity of each time point was normalised to account for the increase in signal over time. Time stamp h:mm:ss.
Part 2. Lattice light-sheet imaging and tracking tissue deformation by PIV. Lateral (top panels) and ventral (bottom panels) views of the VNC (as in part 1) together with corresponding PIV analysis (right panels). Note the wave of VNC motion in a tail to head direction. Time stamp hh:mm:ss.
Part 3. Confocal imaging of VNC condensation and tracking tissue deformation by PIV highlights distinct phases of condensation. (Left panel) Time-lapse imaging of VNC condensation in embryos containing glia labelled for actin (green) and nuclei (magenta). (Right panel) PIV of VNC condensation reveals that the first ∼3 hrs of VNC condensation is asymmetric from tail to head. After the first 3 hrs condensation is predominantly isotropic as the tissue shrinks symmetrically.
Part 4. Tracking glial motion during VNC morphogenesis highlights distinct phases of condensation. Automatic tracking of glia in the head (magenta tracks) and tail (white tracks) of the VNC. Note that during the first ∼3hrs of VNC condensation cell motion is rapid and predominantly in a tail to head direction. In contrast, during the remainder of VNC morphogenesis cells are slower with movement towards the centre of the tissue.
Part 5. Live imaging of VNC morphogenesis and Col4 expression by stereomicroscopy reveals condensation coincides with the induction of Col4. Simultaneous live imaging of VNC condensation and induction of Col4 levels on a stereomicroscope using an endogenous GFP-trap in Col4a2. Example movie for data used for quantification of condensation and Col4 levels as in Figures 1D and 1E.
Part 6. Live imaging of VNC morphogenesis and Col4 expression by confocal microscopy reveals condensation coincides with the induction of Col4. Simultaneous live imaging of VNC condensation and induction of Col4 levels by confocal microscopy using an endogenous GFP-trap in Col4a2. Note that as VNC condensation starts, Col4 rapidly begins to assemble on tissues throughout the embryo.
Part 7. Hemocyte embryonic dispersal correlates with the initiation of VNC condensation. Simultaneous live imaging of VNC (green) condensation and the embryonic migration of hemocytes (magenta) revealing that condensation coincides with the even hemocyte dispersal within the embryo. Time stamp hh:mm.

The VNC is composed of a central nerve bundle surrounded by supporting glial cells, and previous work revealed that affecting glial or neuronal activity perturbs condensation.^11^ However, inhibiting cell activity by driving a dominant negative (DN) Myosin-II or Rac GTPase in glia or neurons led to relatively minor effects on condensation, whereby the 1^st^ phase was left intact (Figures S1C–S1H). In addition, tracking glial movement during the 1^st^ phase revealed a coherent flow of cells without any cellular rearrangements that could explain the VNC morphodynamics (Figures S1I–S1L; Video S1, part 4). Consistent with these results, recent work revealed that the genetic ablation of glia or neurons and RNAi knockdown of Myosin-II in glia left the initial fast phase of VNC condensation intact^12^ (Figure S1D). However, unlike what was previously reported, we did not observe any oscillatory behavior during the 1^st^ phase of condensation.^12^ Instead, we observed a highly coordinated anisotropic movement of VNC segments across the tissue rather than independently contracting elements. These data suggest that there are VNC cell-extrinsic forces driving the 1^st^ phase of condensation, and we therefore set out to determine what was initiating the process.

### Initiation of VNC condensation is correlated with hemocyte deposition of Col4 on the tissue surface and a sudden increase in tissue stiffness

There is an underexplored function of the ECM during *Drosophila* VNC morphogenesis. The VNC is ensheathed by a basement membrane (BM) composed of collagen type IV (Col4) and Laminin, and mutations in BM components perturb condensation.^13^^,^^14^ Correlation of VNC condensation with Col4 levels using a GFP-trap in the Col4α2 subunit revealed that the initiation of the process coincided with the embryonic induction of Col4 expression and an exponential increase in production (Figures 1D and 1E; Video S1, parts 5 and 6). By contrast, Laminin is produced at much earlier stages of embryogenesis,^8^^,^^9^ which led us to hypothesize that Col4 assembly was specifically involved in triggering the start of VNC morphogenesis.

**Figure 2:**
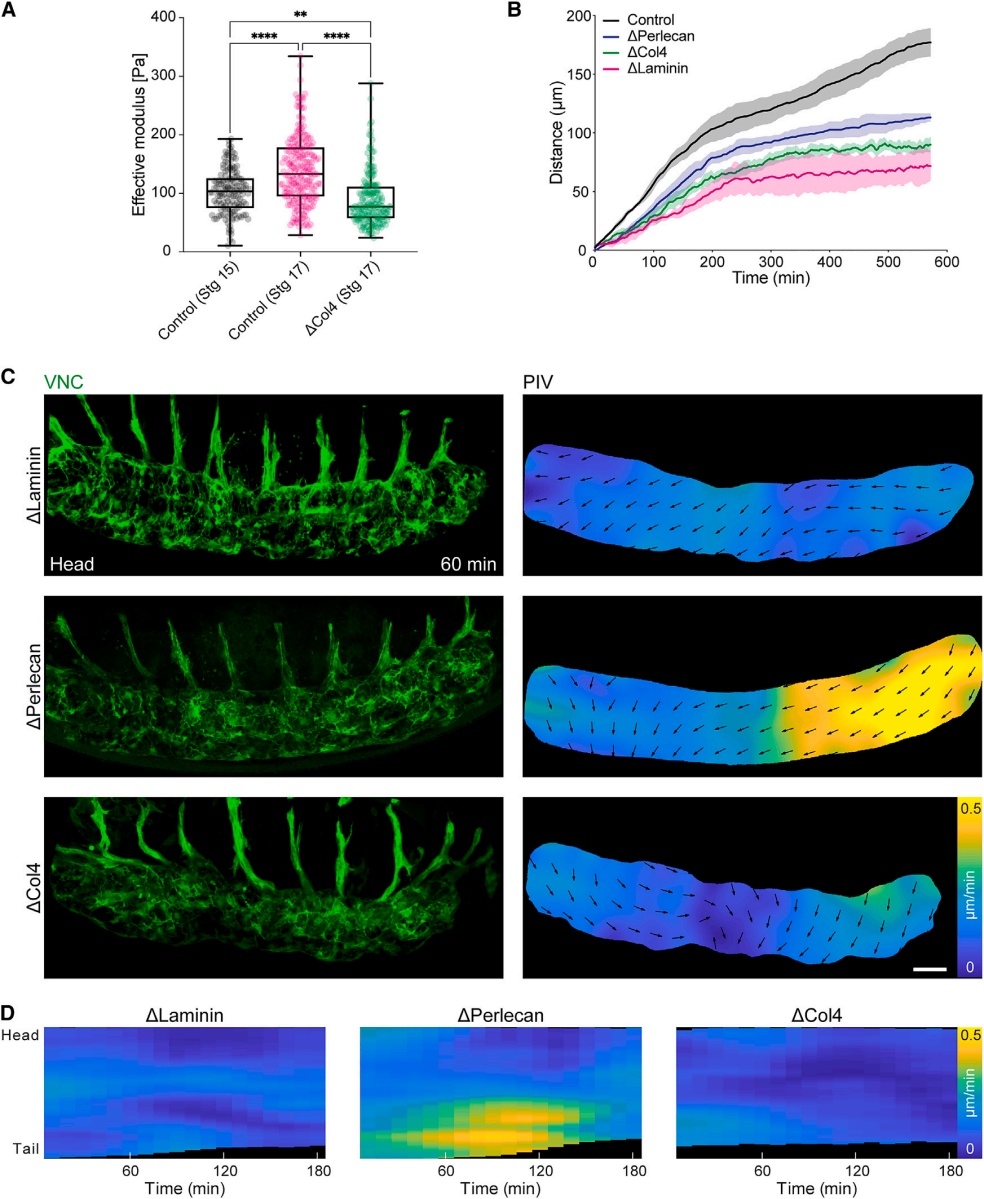
Col4 induction correlates with an increase in VNC stiffness, and the loss of Col4 inhibits the anisotropic phase of VNC condensation (A) Atomic force microscopy of the VNC revealing an increase in stiffness from stage 15 to 17 (1^st^ and 2^nd^ phases, respectively), which is lost in the absence of Col4. Kruskal-Wallis tests and Dunn’s multiple comparisons tests. n = 137 indentations (one control embryo, stg 15), n = 207 (two control embryos, stg 17), n = 181 (two ΔCol4 embryos, stg 17). Each dot represents one indentation. Boxplots show medians, 25th and 75th percentiles as box limits, 10th and 90th percentiles as whiskers. ∗∗∗∗p < 0.0001, ∗∗p = 0.0018. (B) Quantification of VNC condensation rate by tracking tail motion as in Figures 1D and 1E in control (n = 4 embryos) and *laminin*, *perlecan*, and *col4* mutant embryos (n = 3 embryos each). (C) Live imaging of the 1^st^ phase of VNC condensation as in Figure 1A in *laminin*, *perlecan*, and *col4* mutants. Scale bar, 30 μm. (D) Kymograph of the average speed of VNC condensation from PIV analysis in (C) highlighting an absence of an anisotropic phase of condensation in *laminin* and *col4* mutants. See also Video S2.

*Drosophila* hemocytes (macrophages), which are also hypothesized to be involved in VNC condensation,^9^^,^^11^ are the major producers of Col4 during embryogenesis^9^ and their developmental dispersal from their birth in the head mesoderm coincides with the start of condensation (Video S1, part 7). Hemocytes deposit Col4 around the VNC as they disperse evenly throughout the embryo,^9^ which consequently results in a transient gradient of Col4 from head to tail along the tissue (Figures S2A and S2B). Interestingly, this is consistent with a recently observed head-to-tail gradient in VNC stiffness^12^ along with data in *Drosophila* egg chambers revealing that graded Col4 assembly translates into graded BM stiffness.^15^ This suggests that local differences in Col4 assembly can indeed lead to a mechanical gradient across the VNC surface. Subsequently, as condensation proceeds, the Col4 gradient equilibrated such that by the start of the 2^nd^ phase, there was an even distribution of Col4 around the VNC (Figures S2A and S2B). Col4 induction also correlated with an increase in VNC stiffness from the 1^st^ to the 2^nd^ phase of condensation, consistent with data revealing that BM stiffness mainly depends on Col4^16^ (Figure 2A). These data suggest that Col4 assembly was suddenly altering the mechanical properties of the tissue.

### Inhibiting Col4 deposition or altering its distribution along the VNC surface prevents the initial anisotropic change in VNC morphology

Analyses of BM mutants revealed that in the absence of Laminin, which is required for subsequent BM component assembly,^9^ condensation is severely affected and the VNC falls apart due to cell clumping (Figures 2B–2D; Video S2, part 1). By contrast, in the absence of Perlecan, which is expressed slightly later during embryogenesis than Col4,^9^ condensation was slower and the 2^nd^ phase was severely affected; however, there was a clear asymmetric 1^st^ phase of VNC morphogenesis (Figures 2B–2D; Video S2, part 1). However, Col4 mutants had a distinct phenotype: Col4 mutant VNCs remained intact, yet deformed, and both phases of VNC condensation were severely inhibited (Figures 2B–2D; Video S2, part 1). Importantly, the 1^st^ phase of condensation was lost in the absence of Col4 and what little condensation was present was isotropic rather than asymmetric (Figures 2C and 2D; Video S2, part 1). In addition, Col4 mutants failed to show an increase in tissue stiffness (Figure 2A). The Col4 mutant VNC phenotype was specific to Col4 assembly around the tissue as local disruption of Col4 by expression of a surface-bound matrix metalloprotease (*Drosophila* MMP2)^17^^,^^18^ with the same glial-Gal4 driver used to inhibit glial cell activity, phenocopied the Col4 mutant (Figures S2C and S2D). Genetically deleting hemocytes, a major source of embryonic Col4, also reduced VNC shortening (Figure S3). Furthermore, preventing hemocyte release from the head of the embryo by expression of Rac DN, which causes a relative overabundance of Col4 in the head and an exacerbation of the Col4 gradient^9^ led to an almost complete cessation of VNC condensation and abnormal deformation of the tissue (Figure S3; Video S2, parts 2 and 3). Therefore, similar to what was previously observed,^11^^,^^19^ disrupting hemocytes and Col4 distribution leads to a far more severe effect on VNC condensation than inhibiting VNC cellular activity.

Video S2. BM mutations and perturbation of hemocytes prevents the initiation of VNC condensation, related to Figures 2 and S3 Part 1. Live imaging of VNC condensation in *laminin*, *perlecan*, and *col4* mutants. (Left panels) Time-lapse imaging of VNC morphogenesis during the 1^st^ phase of condensation in embryos containing glia labeled for actin in *laminin*, *perlecan*, and *col4* mutants. Note the specific disintegration of the VNC in the absence of laminin. (Right panels) PIV of VNC condensation in the different mutants reveals that *laminin* and *col4* mutants specifically lack an asymmetric 1^st^ phase of condensation.
Part 2. Live imaging of VNC condensation after perturbation of hemocytes. (Left panels) Time-lapse imaging of hemocyte migration (magenta) and VNC condensation (green) in control embryos, embryos lacking hemocytes, and embryos containing hemocytes expressing Rac DN to inhibit their migration from the head of the embryo. Note the herniation of the gut specifically during inhibition of hemocyte migration, which appears to severely deform the VNC. (Right panels) PIV of VNC morphogenesis after perturbation of hemocytes reveals a severe defect in the 1^st^ phase of condensation.
Part 3. Live imaging of VNC condensation after perturbation of hemocyte migration. Time-lapse imaging of VNC condensation in control embryos, embryos containing hemocytes expressing Rac DN to inhibit their migration, and embryos containing hemocytes expressing Rac DN and a dominant negative Col4 transgene (G552D). Note the complete inhibition of VNC condensation after inhibition of hemocyte migration and an apparent severing of the VNC in the middle of the embryo, which is partly rescued by inhibition of Col4. Blue and green arrows highlight the tail of the VNC at the start and end of imaging, respectively.

### Finite element analysis reveals that the initial anisotropic change in VNC morphology can be explained by a sudden increase in asymmetric surface tension

**Figure 3:**
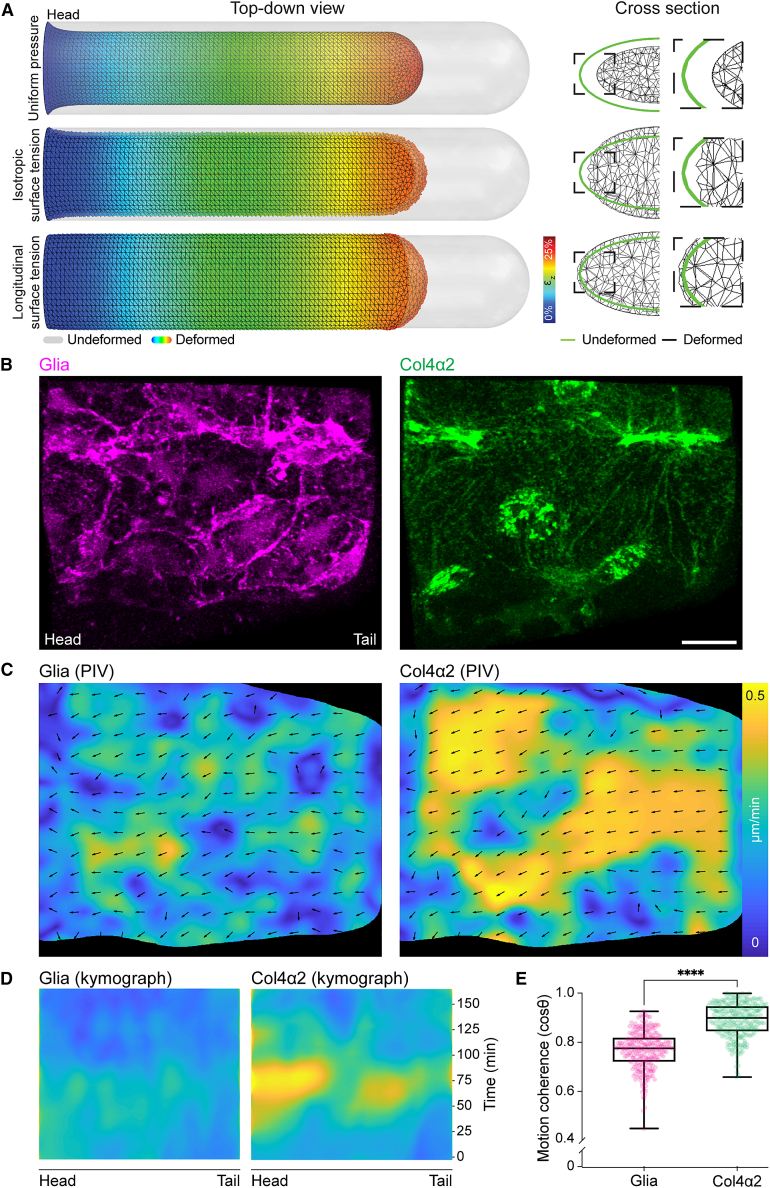
Anisotropy in surface tension and coherent long-range flow of Col4 is sufficient to explain the sudden isovolumetric change in VNC shape (A) Finite element analysis (FEA) of VNC morphogenesis. Simulations of VNC deformation assuming (top panel) uniform normal surface pressure, (middle panel) uniform increase in surface tension, or (bottom panel) anisotropic increase in surface tension along the length of the tissue. Only anisotropic surface tension leads to reduction in length and increase in height and width of the tissue. (B) Live imaging of glia and Col4 motion on the VNC surface. Scale bar, 10 μm. (C) Simultaneous tracking of glia and Col4 motion by PIV. (D) Kymograph of the PIV analysis in (C) highlighting a sudden increase in Col4 speed during VNC condensation which is not observed by tracking glia. (E) Correlation of local alignment of PIV vectors reveals that motion of the Col4 network is more coherent than glial motion. Mann-Whitney test. n = 256 vectors each. Each dot represents one PIV vector. Boxplots show medians, 25th and 75th percentiles as box limits, 10th and 90th percentiles as whiskers. ∗∗∗∗p < 0.0001. See also Video S3.

Polymer networks, especially in thin film organization, are known to generate stresses during assembly, which can lead to polymer motion as stresses equilibrate.^20^ Indeed, inhomogeneities in mixtures of ECM components in a test tube can generate forces that directly drive long-range translocation of inert particles.^5^^,^^6^ To determine whether BM-derived surface stress could contribute to the observed changes in VNC shape on the initiation of condensation, we developed a finite element model consisting of two materials: an internal core representing the VNC attached to the brain, which is surrounded by a thin BM shell. Simulations revealed that a uniform pressure perpendicular to the surface was sufficient to decrease the length of the tissue from tail to head. However, this also led to the constriction of the diameter of the VNC, which was not observed experimentally during the 1^st^ phase of condensation (Figure 3A; Methods S1). In a second approach, we modeled the shell such that it instead exerted a surface tension, which can be modulated by altering its capacity to shrink and its stiffness. Consistent with the Col4-dependent increase in tissue stiffness during condensation, a simulated isotropic increase in surface tension asymmetrically reduced the tissue length; however, this also led to a reduction in tissue width and rounding of nerve cords, which was also not observed in real specimens (Figures 1B, 1C, and 3A; Methods S1). However, further simulations suggested that an anisotropic surface tension, which is predominantly along the length of the tissue, could explain the increase in width and height of the VNC during the 1^st^ phase of condensation (Figures 1B, 1C, and 3A; Methods S1). These data suggest that a BM-dependent increase in tissue surface tension predominantly along the axis of the previously observed stiffness gradient^12^ (and Col4 gradient, this work) can explain the rapid anisotropic change in tissue shape during the initiation of VNC morphogenesis.

### Live imaging of Col4 assembly on the VNC surface reveals a coherent viscous-like flow along the axis of the predicted increase in tissue surface tension

We subsequently live-imaged surface glial dynamics and Col4 accumulation surrounding the VNC at a high spatiotemporal resolution to determine the possible interplay between cell activity and ECM remodeling. As VNC condensation initiated, a rapid and coherent long-range flow of Col4 was observed—preceding glial motion—in a direction antiparallel to the Col4 gradient (i.e., tail to head direction), which was along the predicted predominant axis of surface tension (Figures 3B–3D and S4A–S4E; Video S3, parts 1–3). Subsequently, there was a drift in the surface glia, which are in direct contact with the overlying ECM enveloping the tissue (Figures 3B and 3C; Video S3, parts 2 and 3). At the start of condensation, the surface glia are initially spaced out on the VNC surface and not in contact with each other and eventually undergo a mesenchymal to epithelial transition to form an epithelial monolayer by the end of the 1^st^ phase.^21^^,^^22^ Despite the approximate 16% decrease in VNC surface area during the 1^st^ phase (determined from our measured change in tissue dimensions, Figures 1B and 1C), surface glial cells paradoxically increase in area as they spread themselves around the tissue,^21^^,^^22^ highlighting that glial cell contraction is not involved in this change in tissue geometry. We observed no obvious cell migration or cell rearrangements of glial cells that would explain the overall change in VNC morphology nor did we observe any local ECM remodeling by the underlying glial cells (Video S3, parts 2 and 3). Indeed, correlating the local alignment of particle image velocimetry (PIV) vectors of ECM and glial motion revealed that the Col4 network was moving more coherently over the tissue surface than the cells themselves (Figures 3D and 3E). In addition, the Col4 network appeared to self-assemble with polymers stretching tens of microns linking the dispersed population of glial cells (Figure 3B). Therefore, as predicted of non-uniform thin film growth with a strain or structural gradient,^20^ these data reveal that BM-autonomous assembly dynamics lead to long-range viscous flow and equilibration of the network.

Video S3. The initiation of VNC condensation correlates with coherent Col4 flow along the tissue surface in a tail to head direction, related to Figures 3 and S4 Part 1. Lattice light-sheet imaging and PIV analysis of Col4 and glial motion during initiation of VNC condensation. (Left panels) Time-lapse imaging and PIV analysis of glial motion. (Right panels) Time-lapse imaging and PIV analysis of Col4 motion. Note that the Col4 tail to head motion precedes the glial motion. Time stamp hh:mm.
Part 2. Live imaging and PIV analysis of glial activity and Col4 motion on the VNC surface. (Top panels) Time-lapse imaging of glia containing labeled actin and Col4a2-GFP. (Bottom panels) PIV of glia and Col4 motion. Note the coherent motion of Col4 from tail to head without any noticeable local remodeling by the underlying glial cells.
Part 3. Live imaging and PIV analysis of Col4 motion on the VNC surface in the head vs. tail of the tissue. (Left panels) Simultaneous time-lapse imaging of Col4α2-GFP on the VNC surface in the head vs. tail of the tissue. (Right panels) PIV of Col4 motion in the head vs. tail of the tissue. Note that in the tail of the VNC there is a predominant movement of Col4 towards the head of the tissue. Movies are oriented with the head at the top of the image. Time stamp hh:mm:ss.

### Local disruption of the BM network or perturbation of Col4 assembly alters VNC morphodynamics

**Figure 4:**
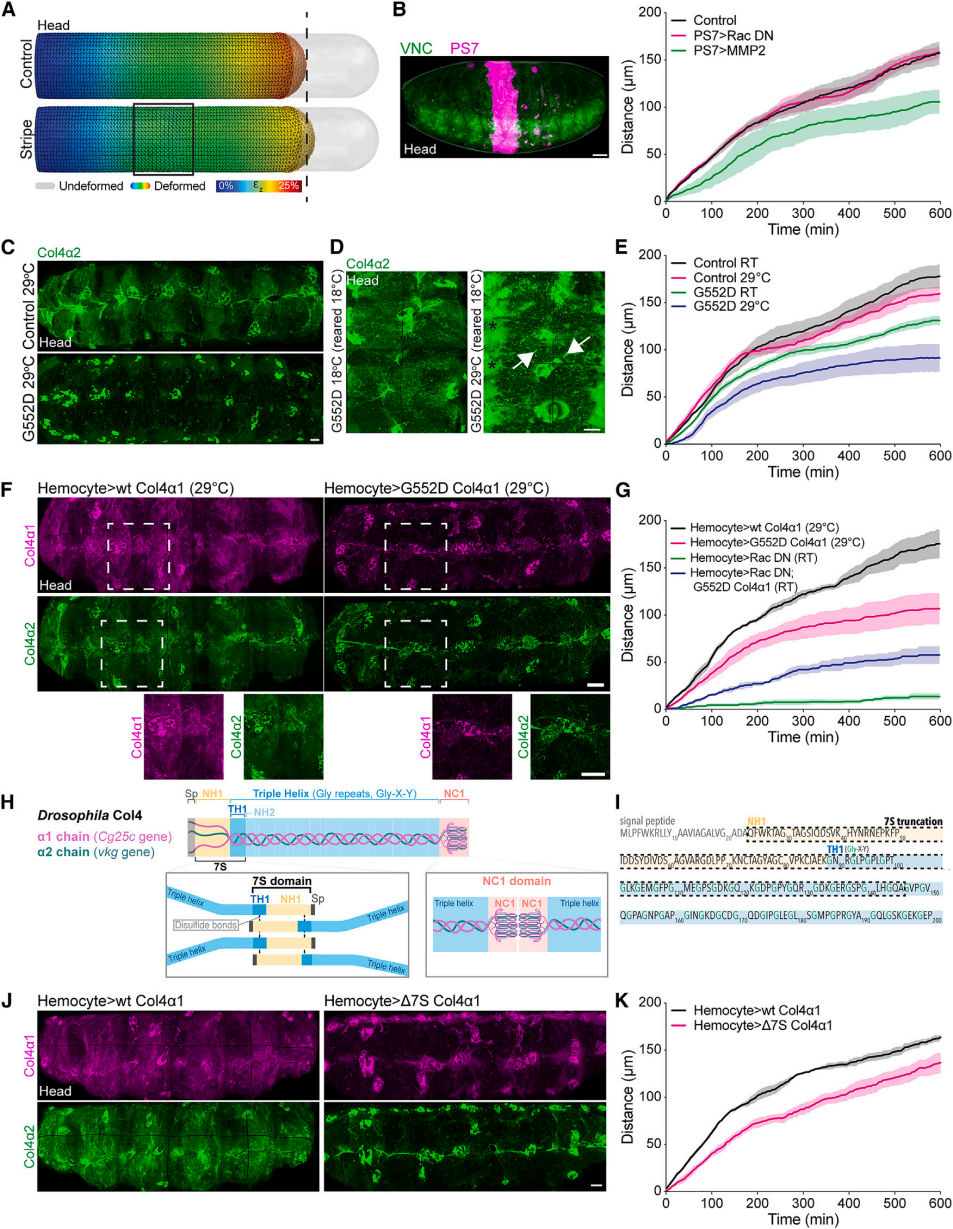
Direct perturbations of Col4 assembly slow the rate of VNC condensation (A) FEA of VNC condensation in which surface tension is locally reduced in a stripe in the middle of the tissue. Simulations suggest that local perturbation of surface tension leads to long-range reduction in deformation of the tail of the tissue. (B) Local disruption of the ECM network by MMP2 expression in a central parasegment (PS7, n = 4), in contrast to control (n = 3) and expression of Rac DN (n = 3), is sufficient to affect the rate of VNC condensation as measured by tracking the tail of the tissue as in Figures 1D and 1E. Scale bar, 30 μm. (C) Live imaging of Col4α2-GFP reveals that rearing embryos containing a temperature-sensitive (TS) point mutation in Col4α1 (G552D) at the non-permissive temperature (29°C) inhibits BM network assembly. Scale bar, 10 μm. (D) Rearing the TS mutant at the permissive temperature (18°C) to allow some ECM assembly and switching to the non-permissive temperature (29°C) leads to aggregation of extracellular Col4 (arrows) and accumulation of soluble Col4 in the hemocoel (asterisks) showing that the TS mutant affects the Col4 network. Scale bar, 10 μm. (E) Quantification of VNC condensation in the TS mutant reveals that the condensation rate can be affected by temperature titration (RT, room temperature). Control data reused from Figure 2B. n = 4 control RT and 29°C, n = 5 G552D RT and 29°C. (F) Expression of wild-type or G552D point mutant Col4α1 in hemocytes during VNC condensation in the background of endogenously GFP-tagged Col4α2. Bottom panels are high-magnification views of the highlighted regions revealing that the G552D transgene fails to incorporate into the ECM network and inhibits incorporation of Col4α2. Scale bars, 10 μm. (G) Quantification of VNC condensation in the genotypes highlighted in (F) reveals that hemocyte-specific expression of the G552D transgene slows VNC condensation. Furthermore, expression of the G552D mutant in hemocytes that also express Rac DN is sufficient to rescue the severe phenotype. n = 3 Hemocyte>wt Col4α1, Hemocyte>Rac DN; G552D Col4α1, n = 5 Hemocyte>G552D Col4α1; Hemocyte>Rac DN is from Figure S3C. (H) Schematic highlighting Col4 domains and interactions hypothesized to drive network assembly. In the C terminus of Col4 are interactions between NC1 domains, which lead to dimerization of Col4 trimers. At the N terminus are interactions of a putative 7S domain, which consists of the first non-helical (NH1) and triple-helical (TH1) regions of the protein. Disulfide bonds and noncovalent interactions between 7S domains are hypothesized to lead to tetramerization of Col4 trimers. Sp, signal peptide. (I) Amino acid sequence of the N terminus of *Drosophila* Col4. The dashed line highlights the amino acid sequence truncated from the putative 7S domain of Col4α1 to generate a dominant negative transgene. (J) Expression of wild-type (WT) Col4α1 or a truncation of the 7S domain in hemocytes shows that deletion of the 7S domain decreases its incorporation and reduces incorporation of Col4α2. Scale bar, 10 μm. (K) Quantification of VNC condensation after expression of the transgenes in (H) reveals that removal of the 7S domain slows the rate of motion of the tail of the tissue. n = 4 embryos each. See also Video S2.

We next examined perturbations that affect ECM polymerization or network organization on VNC morphodynamics. First, modeling predicted that an increase in uniform surface tension should lead to rounder nerve cords when viewed in cross-section, which was observed in Col4 mutants (Figures S4F and S4G). This suggests that in the absence of Col4 assembly, ensheathment of the VNC by surface glia, which are hypothesized to act as a “compression sock” around the tissue,^12^ results in isotropic surface tension that alters VNC morphology. Simulations further suggested that local changes in surface tension should lead to global effects on condensation rate (Figure 4A; Methods S1). To confirm the presence of long-range network effects, we locally cleaved the ECM network in a narrow stripe along the VNC using a parasegmental driver^23^ with MMP2 expression^17^^,^^18^ and examined VNC motion by tracking the tail of the tissue. Cleaving the ECM network in a stripe in the middle of the VNC slowed the motion of the tail of the tissue, which was over a hundred microns distant to the site of cleavage (Figure 4B). This long-range effect suggests that a coherent and interconnected ECM network is required for VNC morphogenesis. We subsequently examined a temperature-sensitive (TS) point mutant Col4 allele (G552D)^24^^,^^25^ that resulted in the ECM network disintegrating when shifted to 29°C (Figures 4C and 4D). This revealed that subtle alteration of the temperature in this TS mutant was sufficient to affect the overall rate of VNC motion (Figure 4E). In addition, driving a G552D Col4 transgene specifically in hemocytes severely inhibited VNC condensation, phenocopying the Col4 mutant, confirming that hemocyte-derived Col4 is indeed essential for initiating the process (Figures 4F and 4G). In addition, expressing this same DN Col4 transgene (G552D) while also trapping hemocytes in the head partially rescued the severe condensation defect, suggesting that uneven Col4 assembly was causing this severe phenotype (Figure 4G; Video S2, part 3). We also generated an N-terminal truncation in the Col4α1 protein that removed part of the putative 7S domain (Figures 4H and 4I), which is hypothesized to be involved in multivalent interactions important for Col4 network assembly.^26^^,^^27^ Driving this transgene specifically in hemocytes was sufficient to perturb Col4 network formation and subtly affect condensation rate, which we speculate is due to a retardation of Col4 polymerization (Figures 4J and 4K).

## Discussion

The textbook view of morphogenesis has assumed that the changes in tissue shape are driven by a limited repertoire of cell-autonomous activities (e.g., cell rearrangements, shape changes, migrations, and divisions).^1^ However, the supramolecular nature of ECM networks, such as BMs, that rely on underexplored covalent and noncovalent interactions for their self-assembly^8^^,^^28^^,^^29^^,^^30^^,^^31^ should not be neglected as sources of stress that shape an embryo. In addition, surface tension has been predicted to be a dominant force in shaping developing tissues,^32^ and although alterations in surface tension are assumed to be driven by epithelial activity,^33^ BM networks are also likely to be involved. Indeed, in the case of VNC condensation, the sudden exponential increase in Col4 formation around the VNC is behaving as a compression sleeve, which actively envelops the tissue to shrink its surface area, resulting in an initial isovolumetric shape change as surface stresses equilibrate. By contrast, in the 2^nd^ phase of condensation, once the asymmetric Col4 network stresses have dissipated, the cells within the VNC likely take a more active part in the process with the ECM possibly playing a more stereotypical passive role.

There are many reasons why a polymer network such as the BM could generate forces during development. The polymerization process on its own, especially if there are instabilities such as concentration gradients during assembly, will lead to stress development and ECM-autonomous motion. Indeed, *in vitro* polymerization assays have revealed that non-uniform distributions of collagen self-assembly can generate surface tension gradients that lead to a Marangoni flow within the network.^34^ In addition, a mechanical mismatch between a polymer thin film (i.e., BM), and the underlying substrate can also lead to material deformations.^20^^,^^35^ As long-range coherent ECM motion has been observed during several developmental processes,^4^^,^^7^^,^^36^ nonequilibrium phenomena in an immature ECM network in flux may be playing unappreciated active roles in tissue morphogenesis.

### Limitations of the study

Although our data suggest an active role for ECM assembly in generating VNC stresses that initiate morphogenesis, it is difficult to completely rule out any role for cellular activity. Nevertheless, perturbations directly affecting cells within the VNC lead to relatively minor effects and normal initiation of condensation.^11^^,^^12^ Conversely, BM defects are far more severe. Understanding how intrinsic stresses develop in the embryonic BM is non-trivial. It requires a biophysical dissection of nucleation/growth of the polymer network and polymer-substrate interactions, which is currently beyond the resolution limit of *in vivo* imaging. Our work has also left out the remainder of VNC condensation after the initial fast phase, which likely involves glia working in conjunction with the ECM, and it will be interesting to understand their interplay during this cell-dependent phase of the process.

## STAR★Methods

### Key resources table

**Table:** 

REAGENT or RESOURCE	SOURCE	IDENTIFIER
**Experimental models: Organisms/strains**		

*D. melanogaster: w1118*	Bloomington Drosophila Stock Center	RRID: BDSC_3605
*D. melanogaster*: repo-Gal4	Bloomington Drosophila Stock Center	RRID: BDSC_7415
*D. melanogaster*: elav-Gal4	see Luo et al.^37^	N/A
*D. melanogaster*: Sn-Gal4	see Zanet et al.^38^	N/A
*D. melanogaster*: PS7-Gal4	see Bowman et al.^23^	N/A
*D. melanogaster*: UAS-LifeActGFP	see Zamet et al.^38^	N/A
*D. melanogaster*: UAS-RedStinger	Bloomington Drosophila Stock Center	RRID: BDSC_8547
*D. melanogaster*: UAS-MMP2	see Page-McCaw et al.^39^	N/A
*D. melanogaster*: UAS-RacN17	Bloomington Drosophila Stock Center	RRID: BDSC_6292
*D. melanogaster*: UAS- ZipDN-GFP	see Franke et al.^40^	N/A
*D. melanogaster:* UAS-Zip RNAi	Vienna *Drosophila* Resource Center	RRID: Flybase_FBst0470845
*D. melanogaster*: UAS-Col4α2-GFP	see Van de Bor et al.^41^	N/A
*D. melanogaster*: UAS-LifeAct-mScarlet	This paper	N/A
*D. melanogaster*: UAS-Col4α1^wt^-mScarlet	This paper	N/A
*D. melanogaster*: UAS-Col4α1^D7S^-mScarlet	This paper	N/A
*D. melanogaster*: UAS-Col4α1^G552D-^mScarlet	This paper	N/A
*D. melanogaster*: fax::GFP	see Buszczak et al.^42^	N/A
*D. melanogaster*: repo-Cherry	Gift from C. Gabernard	N/A
*D. melanogaster*: elav-mYFP	see Kohsaka et al.^43^	N/A
*D. melanogaster*: srpHemo-H2A::3xmCherry	see Gyoergy et al.^44^	N/A
*D. melanogaster*: ColIVα2 (Vkg)-GFP	see Morin et al.^45^	N/A
*D. melanogaster*: Df(2L)LanB1 (ΔLaminin)	see Urbano et al.^14^	N/A
*D. melanogaster*: Df(2L)BSC172 (ΔCol4)	Bloomington Drosophila Stock Center	RRID: BDSC_9605
*D. melanogaster: Cg25C*^*k13420*^	Kyoto Stock Center	RRID: DGGR_102873
*D. melanogaster: trol*^*null*^ (ΔPerlecan)	see Voigt et al.^46^	N/A
*D. melanogaster*: *srp*^*AS*^	see Rehorn et al.^47^	N/A
*D. melanogaster*: *Col4α1*^*G552D*^	see Kelemen-Valkony et al.^24^	N/A
*D. melanogaster: Mhc*^*1*^	Gift from F. Schnorrer	N/A

**Oligonucleotides**		

Primer: 5' GGGAATTGGCCGGCCA ATTAATTAAATGGGTGTCGCAGATT TGATCAAG 3'	This paper	N/A
Primer: 5' CGCAGACCTAGGAAAGCTA GCTTACTTGTACAGCTCGTCCATGCC 3'	This paper	N/A
Primer: 5' CCCCGTAATGCAGAAGAAGA 3'	This paper	N/A
Primer: 5' GACACTGGACTCGATGGACA 3'	This paper	N/A
Primer: 5' GACACTGGACTCGATGGACA 3'	This paper	N/A
Primer: 5' GCTGGTATTCCCGGAGTTTC 3'	This paper	N/A

**Recombinant DNA**		

Plasmid: pUASt-5C	General Fly Transformation Vectors	RRID: DGRC_1261
Plasmid: pUASt-Col4α1^wt^-mScarlet	see Matsubayashi et al.^8^	N/A
Plasmid: pUASt-attB	General Fly Transformation Vectors	RRID: DGRC_1419
Plasmid: pUASt-LifeAct-mScarlet	This paper	N/A
Plasmid: pUASt-attB-Col4α1^wt^-mScarlet	This paper	N/A
Plasmid: pUASt-attB-Col4α1^D7s^-mScarlet	This paper	N/A
Plasmid: pUASt-attB-Col4α1^G552D^-mScarlet	This paper	N/A

**Software and algorithms**		

LAS AF	Leica	http://leica-microsystems.com/home/
Zen	Carl Zeiss	https://www.zeiss.com/microscopy/int/products/microscope-software/zen-lite.html
Zen Black	Carl Zeiss	https://www.zeiss.com/microscopy/int/products/microscope-software/zen.html#downloads
ImageJ/Fiji	Fiji	http://fiji.sc/
Imaris	Bitplane	https://imaris.oxinst.com
Abaqus	Dassaults Systèmes	https://www.3ds.com/products-services/simulia/products/abaqus/
MATLAB	MathWorks	https://uk.mathworks.com/products/matlab.html
Photoshop	Adobe	https://www.adobe.com/uk/products/photoshop.html
Illustrator	Adobe	https://www.adobe.com/uk/products/illustrator.html
Prism	GraphPad	https://www.graphpad.com
Excel	Microsoft	https://www.microsoft.com/en-gb/microsoft-365/excel

**Other**		

In-Fusion Cloning	Takara Bio USA	638920
*Drosophila* injection service	BestGene	https://thebestgene.com/
10S Voltalef oil	VWR	24627.188
Glass-bottom dishes	WPI	https://www.wpi-europe.com/products/cell-and-tissue/fluorodish-cell-culture/fd35-100.aspx
M205 fluorescent dissection microscope	Leica	http://leica-microsystems.com/home/
PLANAPO 2.0x objective for M205	Leica	10450030
LSM 880 confocal microscope	Carl Zeiss	https://www.zeiss.co.uk/microscopy/dynamic-content/news/2014/news-lsm-880.html
63x NA 1.4 Plan-Apochromat oil objective for LSM 880	Carl Zeiss	https://zeiss.com/corporate/int/home.html
Cooling/Heating Plate TP-CHS-C	TOKAI HIT	https://www.tokaihit.com/products/TP-CHS-C/

### Resource availability

#### Lead contact

Further information and requests for resources and reagents should be directed to and will be fulfilled by the lead contact, Brian Stramer (brian.m.stramer@kcl.ac.uk).

#### Materials availability

*Drosophila* lines generated in this study are available from the lead contact upon reasonable request.

### Experimental model and subject details

#### Fly stocks and preparations

Repo-Gal4^48^ and elav-Gal4^37^ were used to target transgene expression in the glial cells and neurons forming the VNC. Sn-Gal4^38^ was used to express transgenes specifically in hemocytes. The parasegmental driver PS7-Gal4^23^ was used to express transgenes in a narrow stripe along the VNC. The following UAS lines were used: UAS-LifeActGFP,^38^ UAS-RedStinger, UAS-MMP2,^39^ UAS-Rac DN,^37^ UAS-Myosin II DN,^40^ UAS-Col4a2-GFP,^41^ UAS-LifeAct-mScarlet, UAS-Col4a1^wt^-mScarlet, UAS- Col4a1^D7S^-mScarlet, and UAS- Col4a1^G552D^-mScarlet (see following sections). Fax::GFP,^42^ repo-Cherry (gift from Clemens Cabernard, University of Basel), and elav-mYFP^43^ were used to label the VNC independently of Gal4. SrpHemo-H2A::3xmCherry^44^ was used to label the nuclei of hemocytes independently of Gal4. The homozygous viable Col4a2-GFP^45^ protein trap was used to visualize Col4. The following mutant alleles and deficiencies were used: Df(2L)LanB1 (referred to as DLaminin, removing lanB1),^14^ Df(2L)BSC172 (referred to as DCol4, removing a chromosomal region including the two *Drosophila* Col IV genes vkg and Cg25C), Cg25C^k13420^ (Col4a1 mutant), trol^null^ (referred to as DPerlecan),^46^ srp^AS^ (lacking hemocytes),^47^ Col4a1^G552D^ (referred as G552D and also known as *DTS-L2*, containing an aspartatic acid substitution in Col4a1 at G552),^24^ *Mhc*^1^ (gift from Frank Schnorrer and characterized for its use in imaging the *Drosophila* embryo in Matsubayashi et al.^8^). Unless used for temperature switch experiments, the flies were left to lay eggs on grape juice agar plates overnight at 25°C. Embryos were dechorionated in bleach. The appropriate genotype of the embryos was identified based on the presence of fluorescent probes and/or the absence of balancer chromosome expressing fluorescent markers. The genotypes of the embryos used in each experiment are listed in the supplemental information (Table S1).

### Method details

#### Construction of UAS-LifeAct-mScarlet, UAS- Col4a1^wt^-mScarlet, UAS- Col4a1^D7s^-mScarlet and UAS-Col4a1^G552D^-mScarlet

pUASt-LifeAct-mScarlet was generated by inserting an 802 bp fragment (synthesized by eurofins Genomics) containing LifeAct followed by mScarlet-I sequences,^49^ and an extra 15 bp at the 3' and 5' ends allowing their insertion into the linearized NheI-PacI pUASt-5C plasmid using In-Fusion cloning strategy (Takara Bio USA, Inc.).

The construct was sequenced (eurofins Genomics) using the following sequencing primers:

5' GGGAATTGGCCGGCCAATTAATTAAATGGGTGTCGCAGATTTGATCAAG 3'
5′ CGCAGACCTAGGAAAGCTAGCTTACTTGTACAGCTCGTCCATGCC 3′

pUASt-attB-Col4a1^wt^-mScarlet was generated inserting a 15 kb fragment containing the Col4a1^wt^-mScarlet sequence from pUASt-Col4a1^wt^-mScarlet,^8^ into the linearized PacI-AvrII pUASt-attB plasmid using ligation T4 strategy (New England Biolabs, Inc.).

pUASt-attB-Col4a1^D7s^-mScarlet was generated by replacing a region of the 7s domain sequence of the pUASt-attB-Col4a1^wt^-mScarlet with a truncated version (Figure S4). A 950 bp fragment containing the 7s region was excised from the plasmid using XhoI and AsiSI sites and substituted by a 588 bp fragment (synthesized by Bio Basic USA, Inc.) containing the 7s truncated region, into the linearised XhoI-AsiSI pUASt-attB-Col4a1^wt^-mScarlet plasmid using ligation T4 strategy (New England Biolabs, Inc.).

The constructs were sequenced using the following sequencing primers:

5' CCCCGTAATGCAGAAGAAGA 3'
5' GACACTGGACTCGATGGACA 3'

pUASt-attB-Col4a1^G552D^-mScarlet was generated by replacing the sequence for the 552 Glycine of Col4a1^wt^ sequence (GGC) of the pUASt-attB-Col4a1^wt^-mScarlet for a new sequence which produces an Aspartic acid (GAC). A 2003 bp region containing the wild-type version of 552 Glycine was excised from the plasmid using AsiSI and EcoNI sites and substituted by a 2003 bp fragment (synthesised by Bio Basic USA, Inc.) containing the sequence for 552 Aspartic, into the linearized AsiSI-EcoNI pUASt-attB-Col4a1^wt^-mScarlet plasmid using ligation T4 strategy (New England Biolabs, Inc.).

The constructs were sequenced using the following sequencing primers:

5' GACACTGGACTCGATGGACA 3'
5' GCTGGTATTCCCGGAGTTTC 3'

The plasmids obtained were injected into flies (BDSC 9744) which contain an attP-9A insertion at 3R chromosome (89E11) by BestGene.

#### Sample preparation and mounting for imaging

For all the confocal imaging, dechorionated embryos were mounted in a drop of Voltalef oil (VWR) between a glass coverslip covered with heptane glue and a gas-permeable Lumox culture dish (Sarstedt) as described previously.^50^ To image the embryos during stage 17 of embryogenesis on the confocal microscope, muscle myosin heavy chain (*Mhc*^1^) mutant embryos that do not twitch but have normal VNC condensation and expression of BM components were used as previously described.^8^ For the widefield imaging, the embryos were mounted in the same way but without heptane glue. For lattice light-sheet imaging, the dechorionated embryos were mounted on a 25 mm glass coverslip covered with heptane glue; the embryos were then covered with a drop of PBS and transferred to the microscope chamber, where imaging was performed in PBS. Temperature controlled experiments were performed by mounting the gas-permeable Lumox culture dish on a TOKAI HIT TP-CHS-C heating/cooling plate.

For the AFM experiments, embryos were prepared as previously described.^9^^,^^51^^,^^52^ After dechorionation with bleach, embryos were transferred to the heptane glue covered glass bottom of a 35 mm cell culture dish (FD35-100) which was then filled with 1x PBS. A cut was made along the dorsal midline of the embryos using an insect pin/needle, exposing the dorsal surface of the VNC.

#### Widefield and confocal microscopy

Widefield images were acquired every 2 min with an M205 fluorescent dissection microscope (Leica) equipped with a PLANAPO 2.0x objective. All the confocal imaging was performed using an LSM880 confocal microscope (Carl Zeiss) equipped with a 63x NA 1.4 Plan-Apochromat oil objective unless stated otherwise. Tilescans (8 x 2 overlap 10%) of 40 mm Z-stacks with a zoom of 1.2 were used for PIV dynamics of the full VNC, with lateral view and temporal resolution of 10 min. For live imaging of Col4 and glia in the tail of the VNC, 20 mm Z-stacks of Col4a2-GFP and repo>LifeAct-mScarlet were acquired with a 63X objective with 2x zoom, and a temporal resolution of 5 min/frame using Optimal Airyscan mode. For simultaneous imaging of Col4 in head vs. tail of the VNC, multipoint acquisition consisting of 20 mm Z-stacks of Col4a2-GFP in head and tail regions of the same embryo were acquired with a 63X objective with 4x zoom, and a temporal resolution of 30 sec/frame using Super Resolution Airyscan mode. To visualize Col4 on the surface of the full VNC with a ventral view, tilescans (10 x 2 overlap 0%) of 20 mm Z-stacks were acquired on Super Resolution Airyscan mode with a zoom of 4. For volume measurements of the full VNC, two tiled images (overlap 10%) of 100 mm Z-stacks with a 20x air objective (Plan-Apochromat air objective, NA 0.8) were acquired with a temporal resolution of 10 min. Images were stitched using the Zen Black software and exported to the Imaris software (Bitplane) for further analysis.

#### Lattice light-sheet microscopy and patterned photobleaching

Lattice light-sheet imaging^53^^,^^54^ and photobleaching of the VNC was performed on the Multimodal Optical Scope with Adaptive Imaging Correction (MOSAIC) system developed at HHMI Janelia Research Campus (unpublished data). Experiments were performed at the HHMI Janelia Research Campus Advanced Imaging Center. For lattice light-sheet imaging, a Thorlabs 0.6 NA water dipping lens (TL20X-MPL) is used for excitation, and a Zeiss 1.0 NA water dipping objective (421452-9800) is used for detection. A square lattice pattern (Inner NA: 0.34; Outer NA: 0.4; Envelope: 3; Crop: 10) was used for generating the lattice light-sheet. Excitation was achieved via 488 nm and 560 nm laser lines, and emission was detected by two Hamamatsu Orca Flash 4.0 sCMOS cameras (dichroic: Semrock Di03-R561-t3-32x40; emissions filters: Camera 1 – Semrock 520/35 nm bandpass filter FF01-520/35-25, Semrock 561 nm notch filter NF03-561E-25; Camera 2 - Semrock 617/73 nm bandpass filter FF02-617/73-25). A system correction for the excitation and detection paths was performed as previously described.^54^ To image the entire depth of the VNC, a 2x2x2 tiled acquisition was performed using the “Z galvo & DO XZ stage” motion, wherein the sample stage is moved directly in line with the optical axis of the detection objective (Single tile size: 512 x 1500 x 201 voxels; voxel size: 108 x 108 x 250 nm; 50 ms per frame, total time between volumes: 5 minutes). To image the surface of the VNC, a 1x2x1 tiled acquisition was performed using the “X Stage” motion, wherein the sample stage is scanned laterally at an angle of 32.45⁰ relative to the optical axis of the detection objective (Single tile size: 512 x 1500 x 251 voxels; voxel size after deskewing: 108 x 108 x 268 nm; 50 ms per frame, total time between volumes: 2 minutes).

The 1x2x1 tiling was used for the photobleaching experiments. Prior to photobleaching, a single, tiled lattice light-sheet volume was collected. The MOSAIC configuration was then automatically switched to a custom point-scanning configuration in which a single Gaussian beam was passed through the lattice light-sheet excitation objective. This beam was dithered in both directions orthogonal to its propagation axis while simultaneously moving the sample stage in the direction of the optical axis of the excitation objective, resulting in a photobleached stripe of approximately 20 μm width perpendicular to the VNC midline. A total of 5 stripes were photobleached along the VNC midline, spaced at approximately 50 μm from each other. After the 5 stripes were photobleached, the MOSAIC configuration was automatically switched back to the lattice light-sheet imaging configuration for subsequent tiled, volumetric imaging as described above.

#### Atomic force microscopy (AFM)

Spherical tipped cantilevers were produced by gluing a 10 μm silica bead at the extremity of qp-CONT probes (Nanosensors). The cantilever spring constant was calibrated in liquid by thermal noise method (0.2 N/m). Measurements were performed using a custom-built system,^55^ based on a standard inverted microscope (Axio Observer.Z1, Zeiss) and equipped with an atomic force microscope (NanoWizard 3, JPK Instruments).

Force spectroscopy measurements using a 3 nN set point were performed on the VNC towards the head side of the tissue.

### Quantification and statistical analysis

#### Lattice light-sheet imaging pre-processing

For lattice light-sheet movies acquired with x stage motion, deskewing is required to reconstruct the stacks in the z direction by correcting the shearing of the data during acquisition.^53^ The pipeline employed to this aim can be found here: https://github.com/aicjanelia/LLSM.

Tilescan stitching used the stitching-spark pipeline developed by the Saalfeld group (https://github.com/saalfeldlab/stitching-spark). The first time point of each dataset was stitched using a Fourier Shift Transform to find the best possible overlap between tiles.^56^ The stitched tile positions from this initial time point were then used for all other time points in the series. The entire time series was exported as blended images for further visualization and analysis.

To run particle image velocimetry (PIV) analysis on lattice light-sheet movies encompassing the whole VNC tissue from the ventral view, 3D segmentation of the tissue was required to remove unwanted signal from imaging planes outside the VNC. To this aim, a segmentation pipeline was implemented, where segmentation is performed manually in MATLAB by drawing polygons around the tissue on sparse z-planes (1 every 20 z-slices) for each time point. By using the segmented planes, a triangulated volume is created and re-sliced to obtain segmentation of intermediate slices.

#### VNC condensation and Col4 levels quantification by fluorescent dissection microscope

For the quantification of VNC condensation, the last position of the tail of the VNC was manually tracked on every frame (2 min time resolution) by using Fiji.^57^ The x and y coordinates of each point were stored and compared to the initial position of the VNC tail to quantify displacement through time.

For the quantification of relative Col4a2-GFP-trap levels through time (2 min time resolution), the average raw fluorescence intensity of each embryo in every frame was measured with Fiji, the acquired data were smoothed by calculating a 10-frame moving average.

#### Volume and surface measurements

To visualise the VNC and measure its volume, the glial cells of the VNC were labelled with UAS-LifeAct-mScarlet under the expression of the glia driver repo-Gal4. To determine the 3 timepoints relevant to the condensation process we proceeded as follows: the beginning of the 1^st^ phase of condensation was determined as the first frame in which displacement of the VNC tail was observed; the end of the 1^st^ phase was determined as 3 hours from the beginning of the 1^st^ phase; and the end of the 2^nd^ phase as 9 hours from the end of the 1^st^ phase. After the frames were selected, each peripheral nerve sprouting from the VNC was manually deleted, by using the “Surface” function in Imaris and by turning the voxels from the magenta channel into zero in the selected areas. Subsequently, the volume of the VNC was obtained by generating a surface on the magenta channel with a voxel of size 2. The length, width, and height of the VNC were quantified with the Imaris function “Measurement Points”. For each embryo, the average height and width were measured at the central point of each neuromere of the VNC. We used the measured VNC dimensions to calculate the change in surface area, using an idealized shape resembling an elliptical cylinder.

#### Quantification of fluorescence intensity in the surface of the VNC

To quantify the Col4 gradient on the VNC surface from confocal images, we first removed the signal from the intra-cellular Col4a2-GFP inside hemocytes by discarding any signal higher than 70% of the total fluorescence intensity. We then calculated the average signal across the VNC from head to tail smoothed with a walking average over 200 μm.

#### Particle image velocimetry (PIV) analysis

PIV was performed with a custom MATLAB suite (https://github.com/stemarcotti/PIV) as in Davis et al.^58^ and Yolland et al.^59^ This methodology was applied on maximum intensity projected time series confocal and lattice light-sheet imaging of the VNC and was used to track the motion on the VNC surface over time. Briefly, the algorithm cross-correlates a small region of interest in a frame of reference (source area) to a larger portion of the subsequent time frame (search area), to find the best match. This operation is performed iteratively on all portions of the image at each time frame to allow for feature tracking in the entire field of view. The parameters were optimized as follows: source size 9 μm, search size 16 μm, grid size 5 μm, correlation threshold 0.5, when the entire VNC was imaged with confocal microscopy; source size 2 μm, search size 4 μm, grid size 1 μm, correlation threshold 0.3, when only a portion of the VNC was imaged with high spatiotemporal resolution with confocal microscopy; source size 5 μm, search size 10 μm, grid size 3 μm, correlation threshold 0.5, when the entire VNC was imaged on the MOSAIC system.

The obtained displacement vector field was then interpolated both spatially and temporally. The interpolation kernel parameters were set as follows: spatial kernel size 50 μm (sigma 10 μm), temporal kernel size 90 min (sigma 40 min), when the entire VNC was imaged with confocal microscopy and the MOSAIC system; spatial kernel size 10 μm (sigma 2 μm), temporal kernel size 5 frames (sigma 2 frames), when only a portion of the VNC was imaged with high spatiotemporal resolution with confocal microscopy. All colourmaps presented were generated to visualize the magnitude of feature velocity, and vectors from the interpolated field were overlaid to highlight flow direction. An average of the velocity magnitude or x component across the length of the VNC was calculated for each frame to plot kymographs.

#### PIV local alignment correlation

To evaluate the coherence of motion for collagen and glia, 256 random locations were selected on the non-interpolated PIV displacement vector field for each time frame. The displacement vector at each of these locations was considered as a reference, and its orientation was compared to all the neighbouring vectors over an 8 μm radius. This was done by calculating the norm of the cosine of the angle theta between the reference vector and each of the neighbouring vectors, and by subsequently obtaining an average across all these comparisons for each location. The computed cosine values were smoothed with a walking average over 5 frames (25 min). Values close to 1 indicate similar orientation between the reference and its neighbours, representing high motion coherence.

A similar approach was taken to measure the coherence of motion for collagen in the tail-to-head direction for two distinct locations at the head and tail of the VNC. For each frame, the direction of 11x11 vectors over a 3 μm-spaced grid of the interpolated PIV field was compared to the reference direction, i.e., tail-to-head direction. The cosine of the angle theta between each vector and the reference vector was calculated as a measure of coherence of motion in the tail-to-head direction, and an average calculated for each frame. Values close to 1 indicate motion in the tail-to-head direction.

#### Cell tracking

Confocal time-lapse movies of the glial cells forming the VNC containing labelled nuclei were tracked with the Imaris function “Spots”. The tracking was divided in two areas, anterior (head) and posterior (tail), which were defined as half of the VNC at the end of the initial 3 hours of VNC condensation. Two 3-hour periods were chosen in the first and second phase, respectively (0-3 hours and 7-10 hours from the start of VNC condensation).

The obtained tracks were analysed with custom software in MATLAB to obtain information on their directionality. The start and end (x,y) coordinates of each track within these time periods which was longer than 10 frames (100 min) were extracted. A vector was drawn between each start and end location and its angle computed to display the polar histograms (angles corresponding to zero were set on the horizontal axis from head to tail). 81 and 130 tracks were analysed for the first phase for head and tail, respectively; 219 and 206 tracks were analysed for the second phase for head and tail, respectively. The mean average velocity of all the tracks with a minimum length of 10 frames (100 min) in both areas was obtained from the Imaris function “Spots”.

#### Analysis of photobleaching

To analyse the anisotropic motion of the VNC when photobleaching stripes across the tissue, a line was drawn in Fiji with thickness 100 px along the midline of the VNC (perpendicular to the stripes). Intensity of the signal in the region highlighted by the linescan was obtained at two different timepoints (immediately and 30 min after photobleaching), normalised and averaged across a moving window of 150 datapoints. The five local minima corresponding to centre of the stripe were automatically located in MATLAB and used to visualized the anisotropic motion from tail to head of the tissue.

#### Eccentricity of VNC cross-sections

MOSAIC movies of the whole VNC with glial cells labelled were used to evaluate the eccentricity of the tissue cross-section. 3D reconstructions were obtained in Imaris and regions corresponding to 3 separate neuromers were selected for two time-points representing stage 15 and 17 of embryo development. Manual segmentation of the tissue was performed in Fiji, and the outlines were centred and aligned to the horizontal axis in MATLAB for visualisation purposes. The eccentricity was calculated in MATLAB as the ratio of the distance between the foci of the ellipse fitting the VNC outline and its major axis length. Values for eccentricity can vary between 0 (circle) and 1 (line).

#### AFM analysis

Analysis of the force spectroscopy curves to obtain the effective modulus was carried out in MATLAB by using custom-made algorithms as described in Marcotti et al.,^60^ considering a target indentation depth for the Hertz model fitting of 230 nm.

#### Statistics and reproducibility

Statistical tests employed are listed in the caption of relevant figure panels. Significance was indicated as follows: ‘^∗∗∗∗^’ for p-values lower than 0.0001, ‘^∗∗∗^’ lower than 0.001, ‘^∗∗^’ lower than 0.01, ‘^∗^’ lower than 0.05, ‘ns’ otherwise.

## Data Availability

Microscopy data reported in this paper will be shared by the lead contact upon reasonable request. The code is deposited in publicly available repositories as specified in the relevant sections below. Any additional information required to reanalyze the data reported in this paper is available from the lead contact upon request.

## Supplemental information

Document S1. Figures S1–S4, Table S1, and Methods S1

Document S2. Article plus supplemental information
