# Supplementary material for: Extracellular matrix assembly stress initiates Drosophila central nervous system morphogenesis
Source: Dev Cell. 2023 May 22;58(10):825–835.e6. doi: 10.1016/j.devcel.2023.03.019 (PMC10390342; doi:10.1016/j.devcel.2023.03.019)
Supplement: Document S1. Figures S1–S4, Table S1, and Methods S1 [file mmc1.pdf]

Developmental Cell, Volume *58*

Supplemental information

Extracellular matrix assembly stress initiates *Drosophila* central nervous system morphogenesis

Eduardo Serna-Morales, Besaiz J. Sánchez-Sánchez, Stefania Marcotti, Angus Nichols, Anushka Bhargava, Anca Dragu, Liisa M. Hirvonen, María-del-Carmen Díaz-de-la-Loza, Matyas Mink, Susan Cox, Emily Rayfield, Rachel M. Lee, Chad M. Hobson, Teng-Leong Chew, and Brian M. Stramer

Supplementary Figures

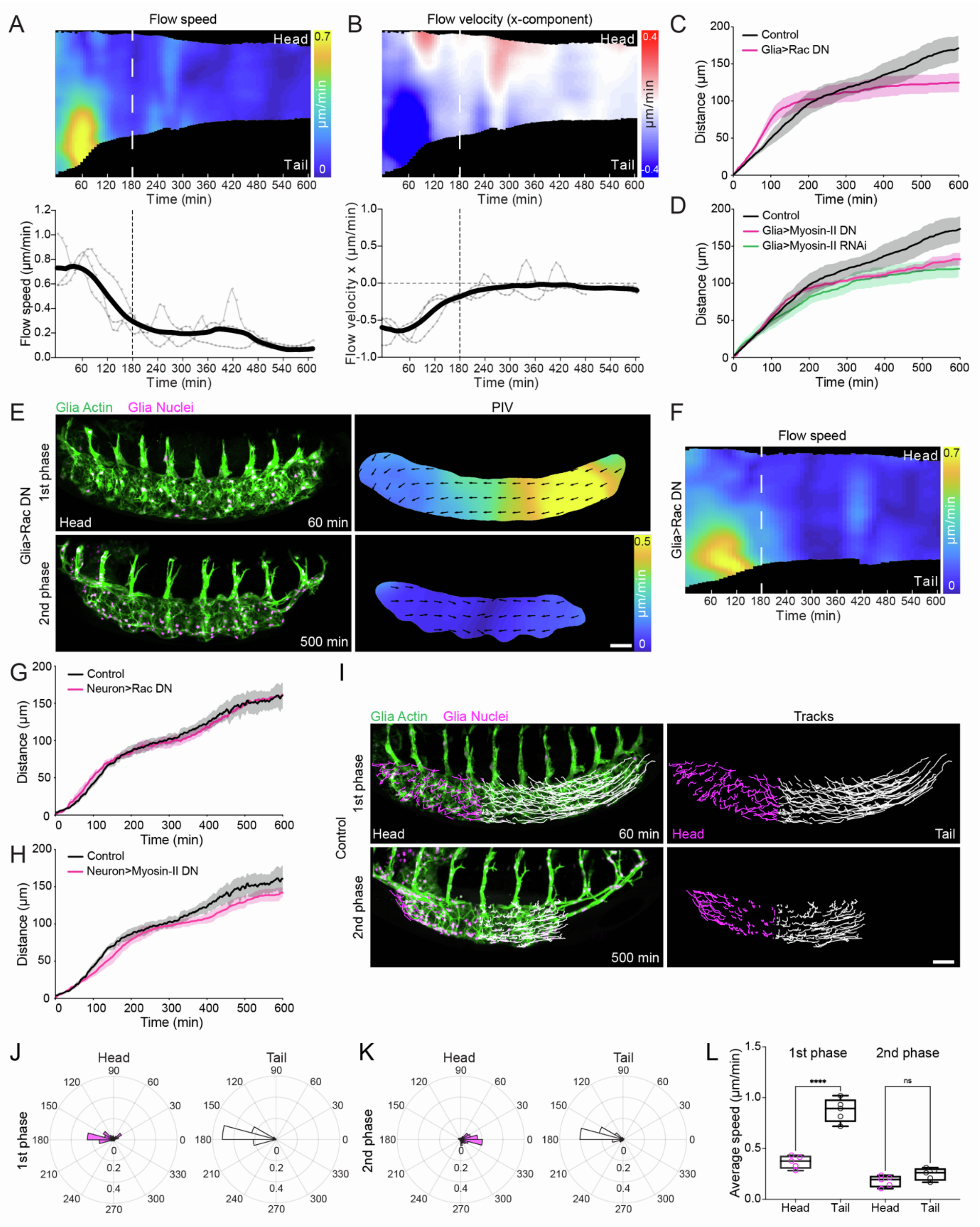

Fig. S1. *Drosophila* VNC condensation consists of two distinct temporal stages and initial anisotropic changes in tissue morphology are independent of VNC cellular activity, related to Figure 1. (A) (top panel) Kymograph of the average speed of VNC condensation from head to tail of the tissue from PIV analysis as shown in Figure 1A. Note the asymmetric increase in speed in the tail of the tissue during the first 3 hours of condensation. (bottom panel) Timecourse of the average speed of VNC condensation as measured above highlighting a transition from fast to slow phases around 3 hours. n = 3 embryos. **(B)** (top panel) Kymograph of the average x-component of the velocity along the length of the tissue from head to tail during VNC condensation. Note the predominant negative velocity in the first 3 hours of condensation, which highlights specific motion of the tail of the tissue towards the head. (bottom panel) Timecourse of the average velocity of the x-component as measured above highlighting that during the first 3 hours condensation is predominantly anisotropic from tail to head (*i.e.*, the x-component is negative). After 3 hours the average x-component approaches zero suggesting that this longer phase of condensation is symmetric (*i.e.*, equivalent from the head and tail of the tissue). n = 3 embryos. **(C,D)** Quantification of VNC condensation by tracking the tail of the tissue as in Figure 1D,E after expressing dominant negative (DN) Rac (C), or DN and RNAi Myosin-II (D) in glia reveals that the rate is only affected during the 2nd phase of condensation. Control data is the same in both panels (n = 4 embryos for control, n = 3 embryos for Glia>Rac DN and Glia>Myosin-II DN). **(E)** Live imaging of VNC morphogenesis as in Figure 1A while expressing Rac DN in glia reveals the presence of an anisotropic 1st phase of condensation. Scale bar = 30 μm. **(F)** Kymograph of the average speed of VNC condensation from head to tail of the tissue from PIV analysis in panel (E). Note the asymmetric increase in speed in the tail of the tissue during the first 3 hours of condensation despite the expression of Rac DN. **(G,H)** Quantification of VNC condensation by tracking the tail of the tissue as in Figure 1D,E after expressing dominant negative (DN) Rac (G) or DN Myosin-II (H) in neurons reveals little if any effect on VNC condensation (n = 3 embryos for each sample). **(I)** Glial cell motion in the head (magenta) vs tail (white) of the tissue tracked during the 1st and 2nd phases of condensation. Scale bar = 30 μm. **(J,K)** Quantification of the direction of tracks in panel (I) reveals that the cells predominantly move in a tail to head direction (towards 180°) during the first phase (J). In contrast, during the isotropic 2nd phase of condensation the cell tracks are predominantly moving symmetrically towards the centre of the VNC (K). n = 3 embryos. **(L)** Quantification of average cell speed during the 1st and 2nd phases of condensation reveals that the motion of cells is fastest within the tail of the tissue during the 1st phase of condensation, while showing no local difference in speed during the 2nd phase. Two-way ANOVA and Tukey's multiple comparisons test. n = 5 embryos. Each dot represents one embryo. Boxplots show medians, 25th and 75th percentiles as box limits, 10th and 90th percentiles as whiskers. ****$p<0.0001$, ns $p = 0.1196$.

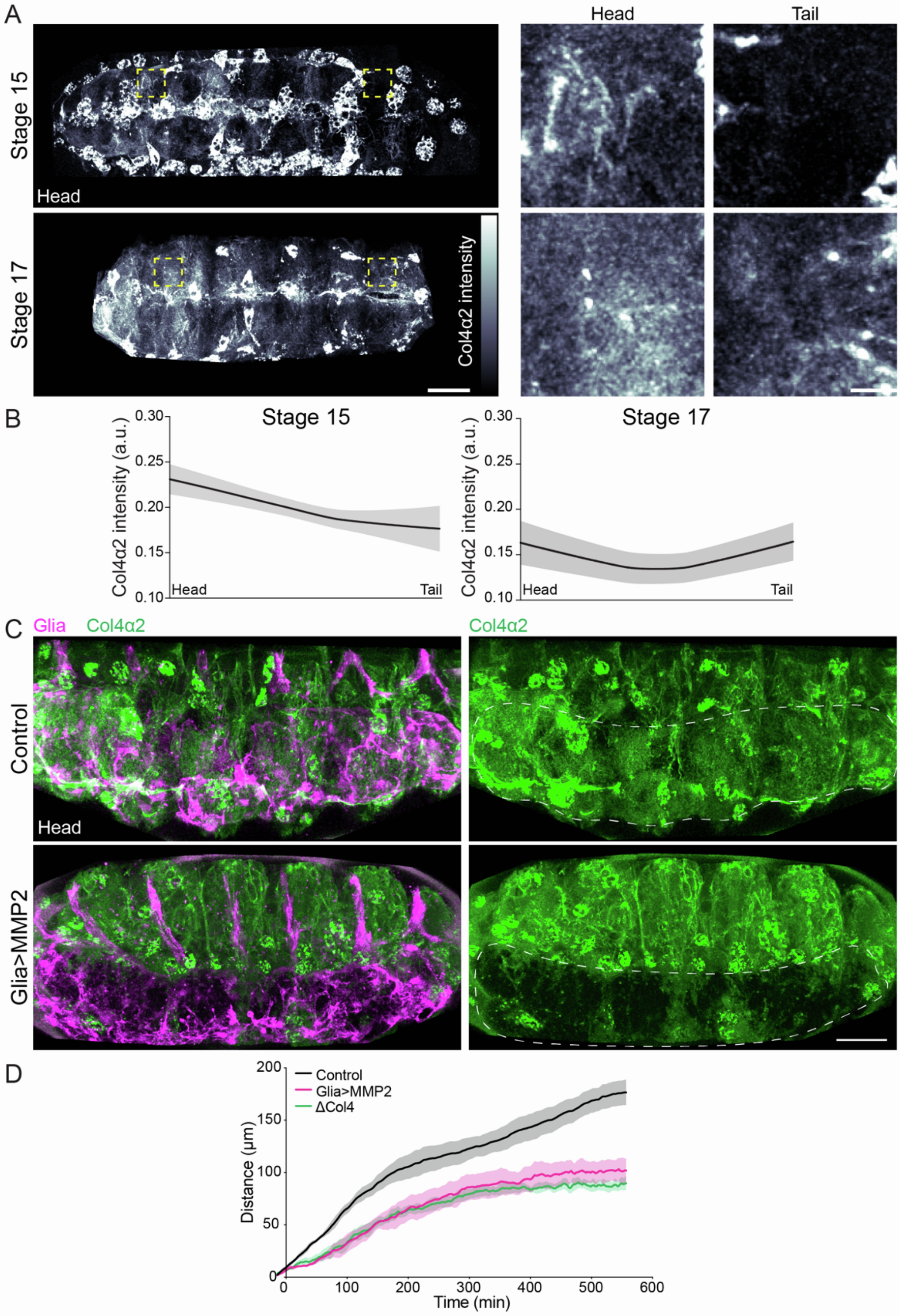
A
Head
Tail
Stage 15
Head
Stage 17
Col4α2 intensity
B
Stage 15
Col4α2 intensity (a.u.)
0.30
0.25
0.20
0.15
0.10
Head
Tail
Stage 17
Col4α2 intensity (a.u.)
0.30
0.25
0.20
0.15
0.10
Head
Tail
C
Glia
Col4α2
Col4α2
Control
Head
Glia>MMP2
D
Control
Glia>MMP2
ΔCol4
Distance (μm)
200
150
100
50
0
Time (min)
0
100
200
300
400
500
600

Fig. S2. Col4 accumulates along the surface of the VNC and forms a transient gradient during the 1st phase of condensation, and local disruption of the Col4 network around the VNC phenocopies defects observed in Col4 mutants, related to Figures 1 and 2. **(A)** Live imaging of Col4 accumulation on the surface of the VNC during the 1st phase (stage 15) and 2nd phases (stage 17) of condensation. Right panels are high magnification views of the regions highlighted by the yellow squares. Scale bars = 30 μm (left panels) or 5 μm (right panels). **(B)** Quantification of Col4 intensity from head to tail of the VNC reveals a transient gradient during the 1st phase of condensation. n = 3 embryos for each stage. **(C)** Live imaging of Col4 and glia in control embryos and embryos driving MMP2 specifically in glia. Note the local disruption of Col4 accumulation surrounding the VNC (white outline). Scale bar = 30 μm. **(D)** Quantification of VNC condensation by tracking the tail of the tissue as in Figure 1D,E in *col4* mutants or after expressing MMP2 in glia reveals similar effects on the rate of condensation. Control and *col4* mutant data reused from Figure 2B (n = 4 embryos for control, n = 3 embryos for Glia>MMP2 and ΔCol4).

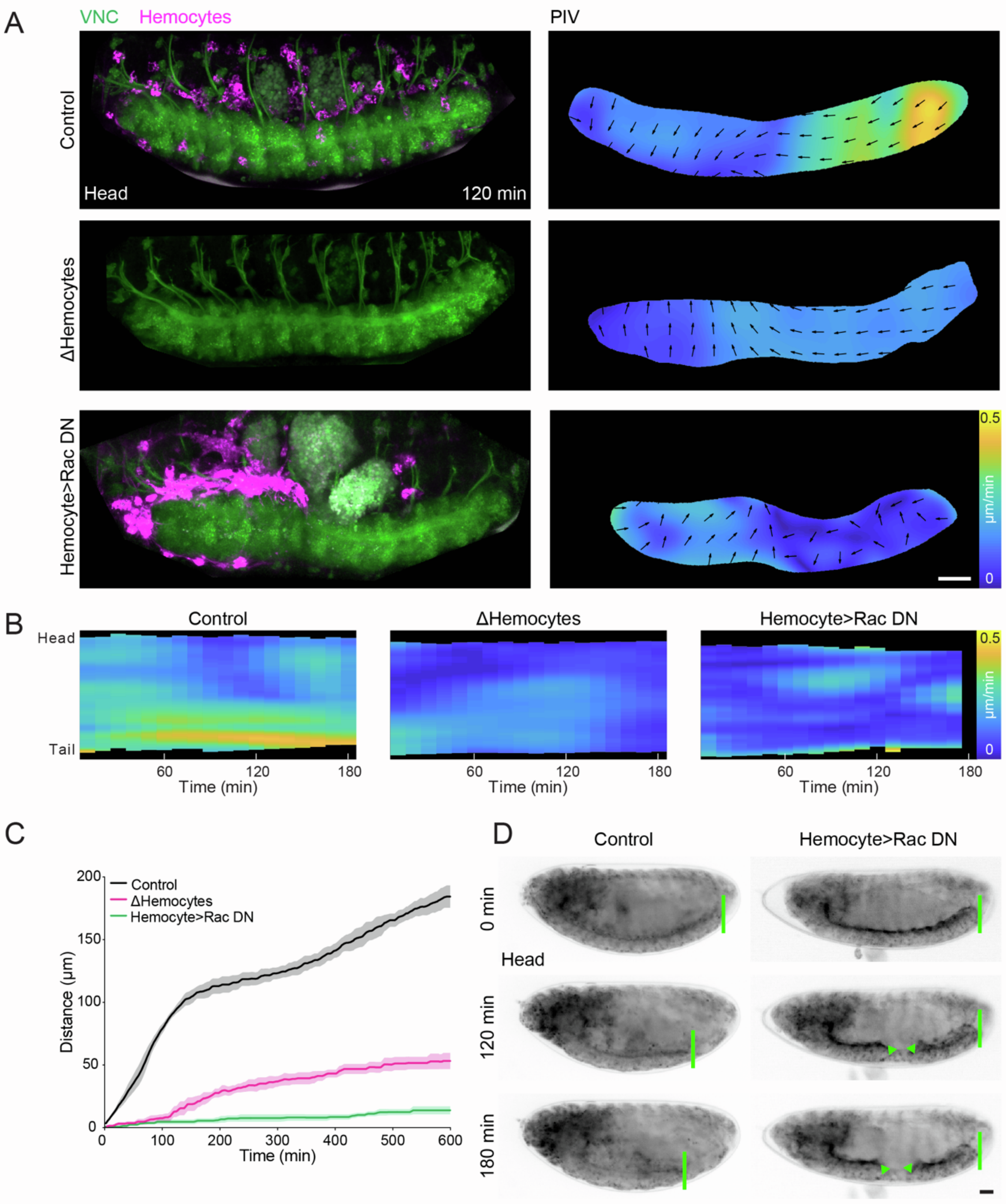
A
VNC
Hemocytes
PIV
Control
Head
120 min
ΔHemocytes
Hemocyte>Rac DN
0.5
µm/min
0
B
Control
ΔHemocytes
Hemocyte>Rac DN
Head
Tail
60
120
180
Time (min)
C
Control
ΔHemocytes
Hemocyte>Rac DN
Distance (µm)
Time (min)
D
Control
Hemocyte>Rac DN
0 min
Head
120 min
180 min

Fig. S3. Inhibiting hemocyte migration severely affects VNC condensation, related to Figure 2. (A) Live imaging of VNC morphogenesis (left panels) and PIV (right panels) during the 1st phase of condensation as in Figure 1A in control, deletion of hemocytes, and expression of Rac DN specifically in hemocytes, which leads to their accumulation in the head of the embryo. Scale bar = 30 μm. **(B)** Kymograph of the average speed of VNC condensation from PIV analysis in (A) highlighting the absence of an anisotropic phase of condensation when perturbing hemocytes. **(C)** Quantification of VNC condensation by tracking the tail of the tissue as in Figure 1D,E in the genotypes highlighted in panel (A) reveals that loss of hemocytes or inhibiting their migration leads to severe defects in the rate of VNC condensation. n = 3 embryos for each sample. **(D)** Time-lapse series of VNC condensation (green lines) in controls and embryos expressing Rac DN specifically in hemocytes. Note that VNC condensation is completely inhibited as the VNC is severely deformed and appears to sever in the center of the embryo (arrowheads). Scale bar = 30 μm.

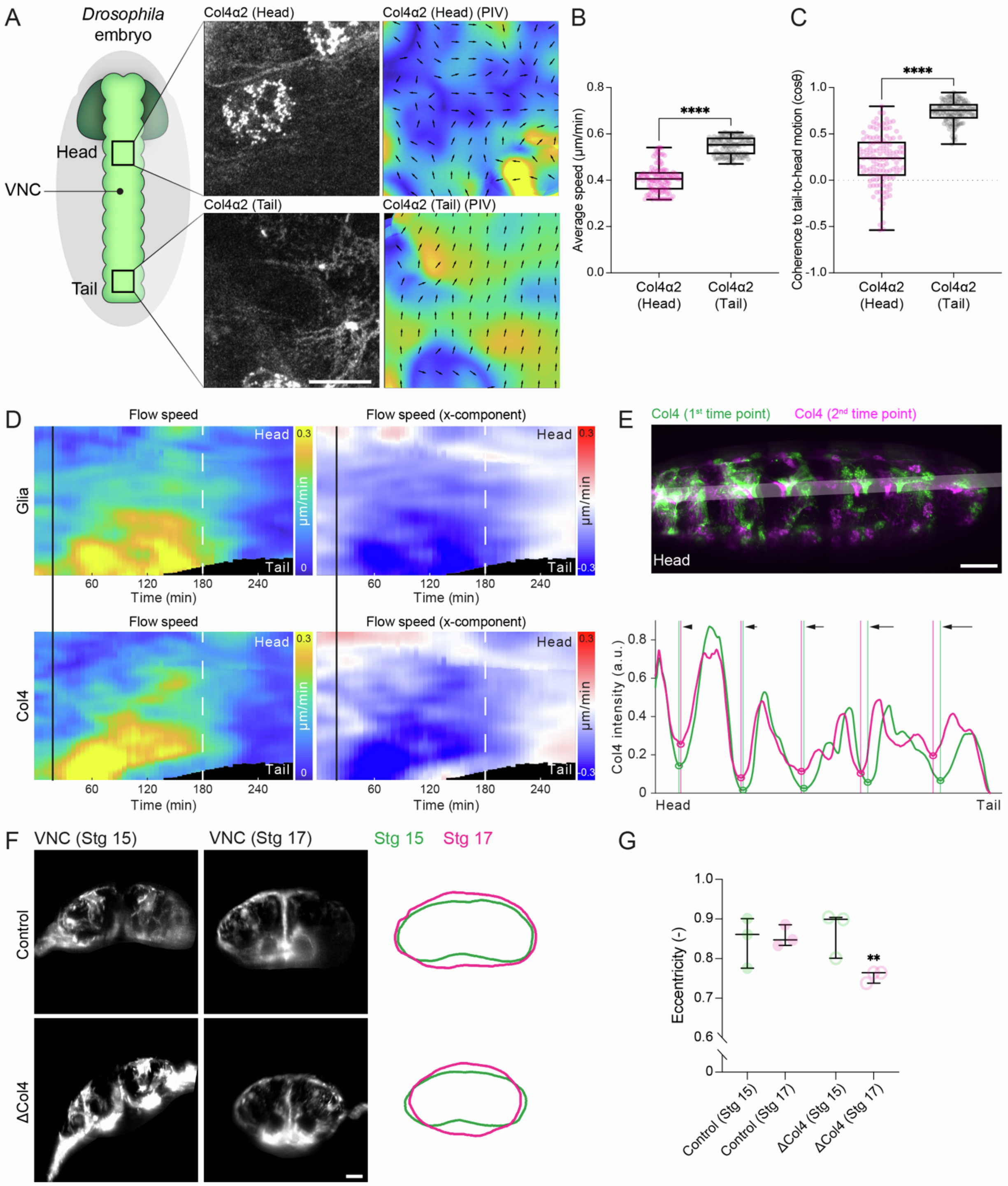

A
Drosophila embryo
Head
VNC
Tail
Col4α2 (Head)
Col4α2 (Head) (PIV)
Col4α2 (Tail)
Col4α2 (Tail) (PIV)
B
Average speed (µm/min)
****
Col4α2 (Head)
Col4α2 (Tail)
C
Coherence to tail-to-head motion (cosθ)
****
Col4α2 (Head)
Col4α2 (Tail)
D
Flow speed
Flow speed (x-component)
Glia
Col4
Head
Tail
µm/min
Time (min)
E
Col4 (1st time point)
Col4 (2nd time point)
Head
Col4 intensity (a.u.)
Head
Tail
F
VNC (Stg 15)
VNC (Stg 17)
Stg 15
Stg 17
Control
ΔCol4
G
Eccentricity (-)
**
Control (Stg 15)
Control (Stg 17)
ΔCol4 (Stg 15)
ΔCol4 (Stg 17)

Fig. S4. Coherent flow of Col4 along the surface of the VNC is towards the head of the tissue, and driving a G552D temperature sensitive Col4 point mutant transgene specifically in hemocytes severely inhibits VNC condensation, related to Figures 3 and 4. (**A**) (Left panels) Col4 was live imaged during the 1st phase of VNC condensation simultaneously in the head and tail regions of the tissue. (Right panels) Tracking of Col4 motion by PIV in head vs. tail regions of the VNC. Scale bar = 10 µm (**B**) Quantification of the average Col4 speed for each frame reveals increased motion in the tail of the VNC when compared with the head. Mann-Whitney test. n = 127 frames for each sample. Each dot represents one frame. Boxplots show medians, 25th and 75th percentiles. ****p<0.0001.
(**C**) Correlation of the global alignment and orientation of PIV vectors for each frame reveals that the motion of the Col4 network is more coherent in the tail of the tissue and oriented towards the head (cosθ of 1 represents a tail-to-head orientation). Mann-Whitney test. n = 127 frames for each sample. Each dot represents one frame. Boxplots show medians, 25th and 75th percentiles. ****p<0.0001. **(D)** Kymographs of the average speed and the average x-component of the velocity of glial and Col4 motion during condensation (as in Figure S1A,B) from PIV analysis of lattice light-sheet imaging. Black line and white dotted line highlight the approximate start of condensation and end of the first phase, respectively. Note that the rapid wave of Col4 motion from tail to head slightly precedes movement of glia. **(E)** (top panel) Equidistant dorsoventral stripes were bleached across the Col4 network, which allowed for direct tracking of BM movement over time from the start of VNC condensation. (bottom panel) Linescan analysis through bleached regions (white shaded stripe) allowed for identification of the bleached fiducial marks over time (green and magenta lines highlight the trough of the bleached stripe at the start of the imaging and after 30 min, respectively). Note that there is progressively greater motion towards the tail of the tissue. Scale bar = 30 µm **(F)** (left panels) Cross sections of control and Col4 mutant (ΔCol4) VNCs at stage 15 (Stg15) and stage 17 (Stg 17) of development, which represent the 1st and 2nd condensation phases, respectively. (right panels) Green and magenta lines highlighting the shape of the VNC in control and Col4 mutant embryos at Stg 15 and Stg 17, respectively. Scale bar = 10 µm **(G)** Quantification of the eccentricity in VNC cross sections in control and Col4 mutants as analysed in (F). Note the decrease in eccentricity in Col4 mutants by stage 17 revealing a more circular shape. One sample t-test against the mean of Control (Stg 15). n = 3 cross-sections for each sample and stage. Each dot represents one cross-section. Boxplots show medians, 25th and 75th percentiles. **p=0.0093.

Supplementary Table

Table S1. Genotypes of the embryos used for each experiment. Related to STAR Methods.

Figure 1	A	w; Mhc^{1}; Repo-Gal4, UAS-LifeActGFP, UAS-RedStinger /+
	B-C	w; Mhc^{1}; Repo-Gal4, UAS-LifeActGFP /+
	D-E	w; Col4α2-GFP; Repo-Gal4, UAS-LifeAct-mScarlet /+
Figure 2	A	"Control": w;; Repo-Gal4, UAS-LifeActGFP /+
		"ΔCol4": w; Df(2L)BSC172; Repo-Gal4, UAS-LifeActGFP /+
	B-D	"Control": w;; Repo-Gal4, UAS-LifeActGFP /+
		"ΔPerlecan": w; trol^{null}; Repo-Gal4, UAS-LifeActGFP /+
		"ΔCol4": w; Df(2L)BSC172; Repo-Gal4, UAS-LifeActGFP /+
		"ΔLaminin": w; Df(2L)LanB1; Repo-Gal4, UAS-LifeActGFP /+
Figure 3	B-E	w; Mhc^{1}, Col4α2-GFP; Repo-Gal4, UAS-LifeAct-mScarlet /+
Figure 4	B	"Control": w; PS7-Gal4 /+; faxGFP /+
		"PS7>RacDN": w; PS7-Gal4 /+; faxGFP / UAS-RacN^{17}
		"PS7>MMP2": w; PS7-Gal4 / UAS-MMP2; faxGFP /+
	C	"Control": w;; Sn-Gal4, UAS-Col4α2-GFP
		"G552D": w; Col4α1^{G552D}; Sn-Gal4, UAS-Col4α2-GFP
	D	"G552D": w; Col4α1^{G552D}; Sn-Gal4, UAS-Col4α2-GFP
	E	"Control": w;; repo-Gal4, UAS-LifeActGFP /+
		"G552D": w; Col4α1^{G552D}; Repo-Gal4, UAS-LifeActGFP /+
	F	"Hemocytes>wt Col4α1":<br>w; Mhc^{1}, Col4α2-GFP; Sn-Gal4, UAS- Col4α1^{wt}-mScarlet
		"Hemocytes>G552D Col4α1":<br>w; Mhc^{1}, Col4α2-GFP; Sn-Gal4, UAS- Col4α1^{G552D}-mScarlet
	G	"Hemocytes>wt Col4α1":<br>w; elav-mYFP; Sn-Gal4, UAS-Col4α1^{wt}-mScarlet
		"Hemocytes>G552D Col4α1":<br>w; elav-mYFP; Sn-Gal4,UAS-Col4α1^{G552D}-mScarlet
		"Hemocytes>RacDN":<br>w; elav-mYFP; Sn-Gal4,UAS-Col4α1^{wt}-mScarlet / UAS-RacN^{17}
		"Hemocytes>RacDN; G552D Col4α1":<br>w; elav-mYFP; Sn-Gal4, UAS- Col4α1^{G552D}-mScarlet / UAS-RacN^{17}
	J	"Hemocyte>wt Col4α1":<br>w; Mhc^{1}, Col4α2-GFP; Sn-Gal4,UAS- Col4α1^{wt}-mScarlet
		"Hemocyte>Δ7S Col4α1":<br>w; Mhc^{1}, Col4α2-GFP; Sn-Gal4, UAS- Col4α1^{Δ7S}-mScarlet
	K	"Hemocyte>wt Col4α1":<br>w; elav-mYFP; Sn-Gal4, UAS-Col4α1^{wt}-mScarlet
		"Hemocyte>Δ7S Col4α1":<br>w; elav-mYFP; Sn-Gal4,UAS-Col4α1^{Δ7S}-mScarlet

Supplementary Figure 1	A-B	w; Mhc^{1}; Repo-Gal4, UAS-LifeActGFP, UAS-RedStinger /+
	C	“Control”: w;; Repo-Gal4, UAS-LifeActGFP /+
		“Glia>RacDN”: w;; Repo-Gal4, UAS-LifeActGFP / UAS-RacN^{17}
	D	“Control”: w;; Repo-Gal4, UAS-LifeActGFP /+
		“Glia>Myosin II DN”: w; UAS-Zipper DN-GFP /+; Repo-Gal4, UAS-LifeActGFP /+
		“Glia>Myosin II RNAi”: w; UAS-Zipper RNAi /+; Repo-Gal4,UAS-LifeActGFP /+
	E-F	w;; Repo-Gal4, UAS-LifeActGFP, UAS-RedStinger / UAS-RacN^{17}
	G	“Control”: Elav-Gal4 /+;;Repo-mCD8::Cherry /+
		“Neuron>RacDN”: Elav-Gal4 /+;;Repo-mCD8::Cherry / UAS-RacN^{17}
	H	“Control”: Elav-Gal4 /+;;Repo-mCD8::Cherry /+
		“Neuron>Myosin II DN”: Elav-Gal4 /+; UAS-Myosin II DN /+; Repo-mCD8::Cherry /+
	I-L	Mhc^{1}; w;; Repo-Gal4, UAS-LifeActGFP, UAS-RedStinger /+
Supplementary Figure 2	A-B	w; Mhc^{1}, Col4α2-GFP
	C	“Control”: w; Col4α2-GFP; Repo-Gal4, UAS-LifeAct-mScarlet /+
		“Glia>MMP2”: w; Col4α2-GFP, UAS-MMP2 / Col4α2-GFP; Repo-Gal4, UAS-LifeAct-mScarlet /+
	D	“Control”: w; Repo-Gal4, UAS-LifeAct-mScarlet /+
		“Glia>MMP2”: w; UAS-MMP2 /+; Repo-Gal4, UAS-LifeAct-mScarlet /+
		“ΔCol4”: w; Df(2L)BSC172; Repo-Gal4, UAS-LifeActGFP /+
Supplementary Figure 3	A-D	“Control”: w; elav-mYFP; Sn-Gal4, UAS- Col4α1^{wt}-mScarlet
		“ΔHemocytes”: w; Srp^{AS}; elav-mYFP
		“Hemocytes>RacDN”: w; elav-mYFP; Sn-Gal4, UAS-Col4α1^{wt}-mScarlet/ UAS-RacN^{17}
Supplementary Figure 4	A-E	w; Mhc^{1}, Col4α2-GFP; Repo-Gal4, UAS-LifeAct-mScarlet /+
	F-G	“Control”: w; Mhc^{1}, Col4α2-GFP; Repo-Gal4, UAS-LifeAct-mScarlet /+
		“ΔCol4”: w; Df(2L)BSC172 / Cg25C^{k13420}; Repo-Gal4, UAS-LifeActGFP /+

Movie 1	Part 1-2	*w; Col4α2-GFP; Repo-Gal4, UAS-LifeAct-mScarlet /+*
	Part 3-4	*w; Mhc*^{1}*; Repo-Gal4, UAS-LifeActGFP, UAS-RedStinger /+*
	Part 5-6	*w; Col4α2-GFP; Repo-Gal4, UAS-LifeAct-mScarlet /+*
	Part 7	*w; elav-mYFP; Sn-Gal4, UAS-RedStinger*
Movie 2	Part 1	“ΔLaminin”:<br>*w; Df(2L)LanB1; Repo-Gal4, UAS-LifeActGFP /+*
		“ΔPerlecan”:<br>*w; trol*^{null}*; Repo-Gal4, UAS-LifeActGFP /+*
		“ΔCol4”:<br>*w; Df(2L)BSC172 / Cg25C*^{k13420}*; Repo-Gal4, UAS-LifeActGFP /+*
	Part 2	“Control”:<br>*w; elav-mYFP; Sn-Gal4, UAS- Col4α1*^{wt}*-mScarlet*
		“ΔHemocytes”:<br>*w; Srp*^{AS}*; elav-mYFP*
		“Hemocytes>RacDN”:<br>*w; elav-mYFP; Sn-Gal4, UAS-Col4α1*^{wt}*-mScarlet / UAS-RacN*^{17}
	Part 3	“Control”: *w; elav-mYFP; Sn-Gal4, UAS-Col4α1*^{wt}*-mScarlet*
		“Hemocytes>RacDN”:<br>*w; elav-mYFP; Sn-Gal4, UAS-Col4α1*^{wt}*-mScarlet / UAS-RacN*^{17}
		“Hemocytes>RacDN”:<br>*w; elav-mYFP; Sn-Gal4, UAS-Col4α1*^{G552D}*-mScarlet/ UAS-RacN*^{17}
Movie 3	Part 1-3	*w; Mhc*^{1}*, Col4α2-GFP; Repo-Gal4, UAS-LifeAct-mScarlet /+*

Supplementary Methods

Methods S1. Supplementary methods for FE modelling. Related to STAR Methods.

1 Introduction

In this document, we illustrate in further detail the Finite Element (FE) model simulations of ventral nerve cord (VNC) condensation presented in the main text. Information on how the model was constructed, on how simulations were run and on parameters choice are given.

VNC morphogenesis involves two distinct phases, a first rapid isovolumetric phase lasting about 3 hours which shows an anisotropic 25% reduction in the length of the tissue from tail to head, and a second slower phase which shows a more isotropic condensation and a reduction in volume. As most of the morphological changes happen in the first phase of condensation, the modelling approach presented here focuses on this part of the process, with the aim of explaining the loading conditions necessary to initiate the anisotropic motion.

All FE simulations were performed in Abaqus (Simulia, Dassault Systèmes) by using static modelling. An idealised geometry was employed with dimensions acquired through experimental observations (Figure 1C in the main text) and different loading conditions were simulated.

2 FE model

An idealised shape representing the approximate VNC morphology was designed as an elliptical cylinder (Fig. S5). The head side of the VNC is physically connected to the embryonic brain, while the tail of the tissue is free to move and has a semi-spherical end. Relevant dimensions were chosen according to experimental observations (Figure 1C in the main text), with the elliptical cylinder measuring 400 μm in length, and displaying a cross section of major and minor axes measuring 83 μm and 38 μm, respectively.

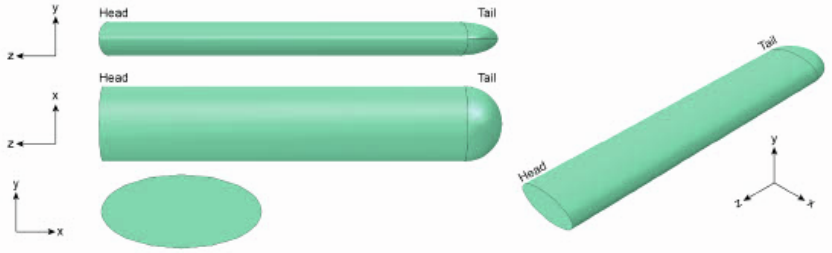

Fig. S5: Geometry of the FE model, related to Figure 3. Orthogonal views of the FE model of the VNC. The model is constituted by an elliptical cylindrical shape which dimensions were selected to match the experimental observations. The head side is connected to the tissue forming the brain, while the tail end is free to move.

The model was constituted by two materials, a tissue core which represents glia and neurons, and a surrounding thin shell representing the basement membrane. This surrounding layer was modelled as a 0.1 μm-thick skin shell. Both materials were defined as linear elastic with Poisson ratio of 0.48. Default Young's moduli were set to 70 Pa for the tissue and 30 Pa for the basement membrane, if not otherwise specified in certain simulation cases. These values were chosen to match the experimental observations performed by atomic force microscopy (Figure 2A in the main text). A shrinking coefficient was assigned to the shell layer when simulating surface tension.

To perform FE analysis, the geometry has to be meshed into a finite number of elements of known dimension. The geometry was therefore meshed with a total of 79818 nodes and 61689 elements for analysis, corresponding to elements of about 5 μm in size (Fig. S6). Two different types of elements were used: 7436 linear triangular elements for the shell (type S3) and 54253 quadratic tetrahedral elements for the tissue (type C3D10). Convergence tests were run for a range of different element dimensions (from 2.5 to 20 μm), each analysis showed little variation in the simulated reduction in length when loading the model with uniform pressure.

The model was constrained at the head side, where the VNC is attached to the brain tissue in the *Drosophila* embryo. As the brain tissue is of considerable dimensions compared to the VNC, it was hypothesised that this link could be simulated with an encastre, constraining all translational and rotational degrees of motion (Fig. S7).

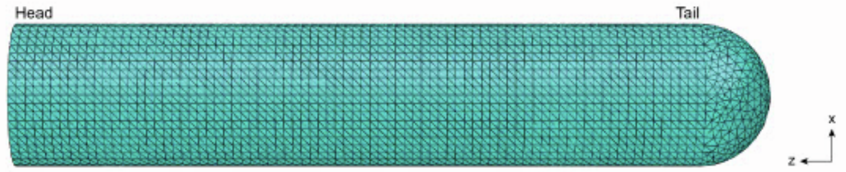

Fig. S6: Mesh of the FE model, related to Figure 3. Top-down view of the FE model showing the mesh used, with elements of about 5 μm in size. Linear triangular elements or quadratic tetrahedral elements were employed for the shell and tissue, respectively.

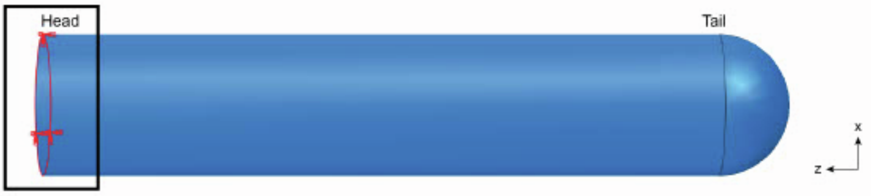

Fig. S7: Boundary conditions for the FE model, related to Figure 3. The model was constrained by imposing an encastre at the head side (black square) simulating its attachment to the brain tissue in the *Drosophila* embryo.

3 Loading scenarios

Different loading scenarios were simulated with the goal of reproducing the experimentally observed change in shape of the VNC during the first phase of morphogenesis. This phase of the process is characterised by an anisotropic reduction in length from the tail to the head of the tissue. The first phase does not display a change in the volume of the VNC, and the cross-section of the tissue expands along both axes to compensate for the reduction in length (Figure 1C in the main text).

The different simulations performed and their rationales are outlined in the following sections. The simulated magnitude of the displacement along the length of the VNC was used as output, as a proxy for the experimentally observed motion of the tissue.

3.1 Uniform pressure

We first loaded the model with a uniform pressure, normal to each element. This would correspond to a cell-driven active compression applied on the tissue, as the cells would locally contract resulting in a perpendicular force on the VNC. This loading scenario caused a reduction in length from tail to head as experimentally observed; however, it was accompanied by a reduction in the size of both axes in the cross-section, suggesting a change in volume which diverged from experimental data (Fig. S8). Increasing the magnitude of the applied pressure resulted in a more marked shrinking in all axes (Fig. S9). To obtain a reduction in length of 25%, a uniform pressure of 0.38 kPa was applied.

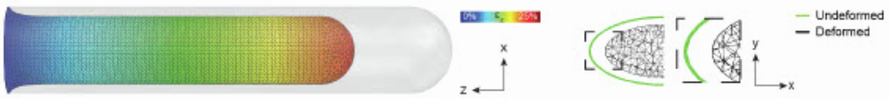

Fig. S8: Loading the FE model with a uniform pressure, related to Figure 3. Simulating loading with a perpendicular uniform pressure on the VNC resulted in its anisotropic reduction in length from tail to head (left panel). However, reduction in both the height and width of the cross-section was also observed (right panel), diverging from experimental evidence. Colours represent the magnitude of displacement along the length of the tissue (z-axis) with values ranging from 0% to 25% of strain.

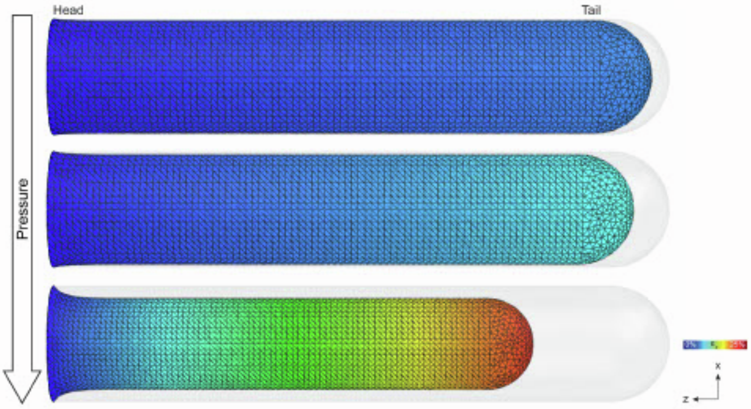

Fig. S9: Loading the FE model with a uniform pressure - effect of increasing the pressure magnitude, related to Figure 3. Increasing the magnitude of the simulated normal pressure exacerbated the amount of length reduction (top panel: pressure magnitude $0.05\ kPa$, middle: $0.1\ kPa$, bottom: $0.38\ kPa$). Colours represent the magnitude of displacement along the length of the tissue (z-axis) with values ranging from 0% to 25% of strain.

3.2 Isotropic surface tension

We then simulated a loading scenario involving surface tension, as we hypothesised a role for the basement membrane layer surrounding the tissue in driving its condensation. To this aim, we assigned a temperature-controlled expansion coefficient to the shell material: by simulating a drop in temperature, we could cause the material of the shell to shrink. Similarly to loading the model with uniform pressure, this simulation resulted in an anisotropic reduction in length of the VNC. When looking at the cross-section of the tissue, we observed the expected increase in size along the y-axis, but also a decrease along the x-axis, with a tendency towards a more circular shape, which did not match with the experimental observations (Fig. S10).

We could modulate the ability of the shell to shrink by keeping the temperature drop constant (i.e., 100 to 0 degrees) and subsequently changing the expansion coefficient (Fig. S11). This resulted in a progressively larger reduction in the VNC length. To simulate a 25% reduction in length, an expansion coefficient of 0.65 was required.

The magnitude of the surface tension could also be adjusted by changing the material properties of the shell. In fact, by increasing the stiffness of the shell, it was possible to increase the reduction in length of the tissue, while keeping constant both the temperature drop (100 to 0 degrees) and expansion coefficient (0.65).

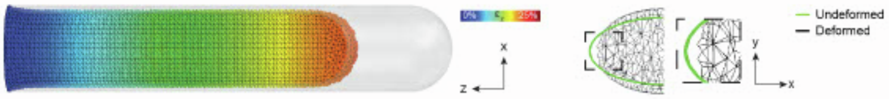

Fig. S10: Loading the FE model with an isotropic surface tension, related to Figure 3. Simulating surface tension by allowing the shell material to shrink resulted in an anisotropic reduction in length towards the head (left panel). The change in shape in the cross-section showed increased dimensions in height and decreased dimensions in width (right panel). Colours represent the magnitude of displacement along the length of the tissue (z-axis) with values ranging from 0% to 25% of strain.

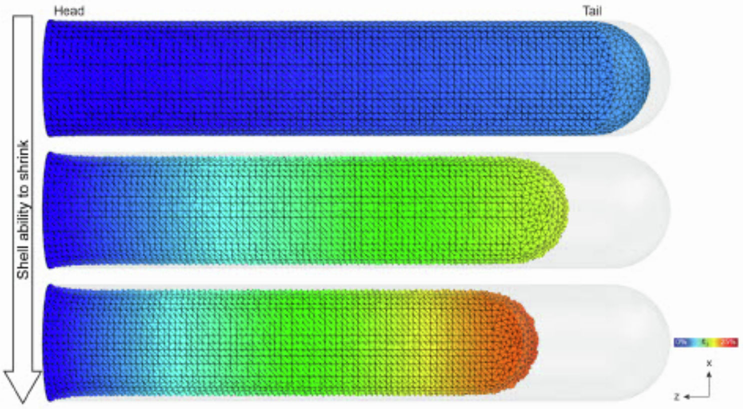

Fig. S11: Loading the FE model with an isotropic surface tension - effect of increasing the shell ability to shrink, related to Figure 3. Simulating surface tension by allowing the shell material to shrink resulted in an anisotropic reduction in length towards the head, proportional to the assigned expansion coefficient (top panel: expansion coefficient 0.1, middle: 0.5, bottom: 0.65). Colours represent the magnitude of displacement along the length of the tissue (z-axis) with values ranging from 0% to 25% of strain.

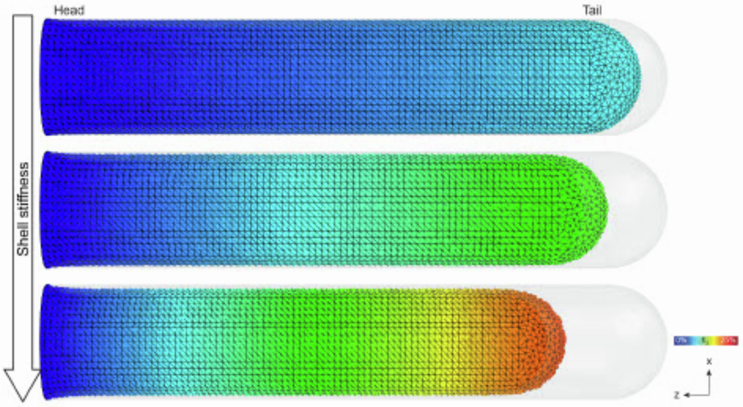

Fig. S12: Loading the FE model with an isotropic surface tension - effect of increasing the shell stiffness, related to Figure 3. Simulating surface tension by allowing the shell material to shrink resulted in an anisotropic reduction in length towards the head, proportional to the assigned shell stiffness (top panel: shell/tissue Young's moduli $10/90\ kPa$, middle: $20/80\ kPa$, bottom: $30/70\ kPa$). Colours represent the magnitude of displacement along the length of the tissue (z-axis) with values ranging from 0% to 25% of strain.

3.3 Longitudinal surface tension

Finally, we simulated a longitudinal surface tension along the length of the tissue (z-axis), as we hypothesised that the observed gradient of collagen from head to tail may lead to a preferential directionality of surface tension (Supplementary Figure 2AB). This was achieved by defining an anisotropic shell shrinking behaviour, which was described by an expansion coefficient for each of the three material orientations (Fig. S13). We set to zero the expansion coefficients for the normal and radial orientations, and only kept active the longitudinal component for each element (0.65), which prevented the tissue from shrinking in the normal and radial axes. This showed an anisotropic reduction in VNC length from tail to head, which correlated with an expansion in both axes of the cross-section (Fig. S14). This result was analogous to what was observed experimentally, suggesting that a longitudinal surface tension dictated by the collagen layer surrounding the tissue might be the driving force of the first phase of the condensation process.

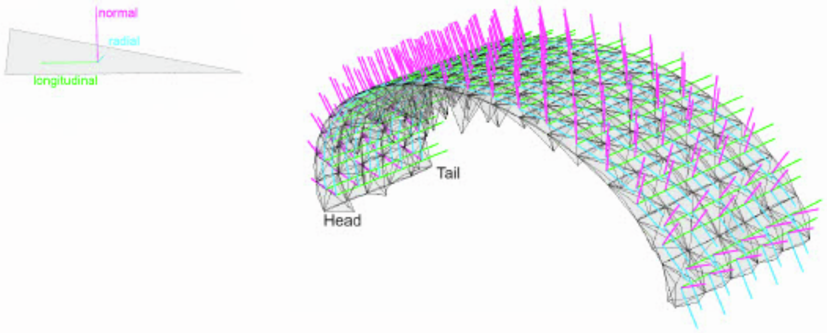

Fig. S13: Loading the FE model with an anisotropic surface tension - evaluation of material orientation, related to Figure 3. Simulating an anisotropic surface tension required assigning an expansion coefficient for each material orientation. Each element is assigned its own reference, with a normal (magenta), radial (cyan), and longitudinal (green) orientation.

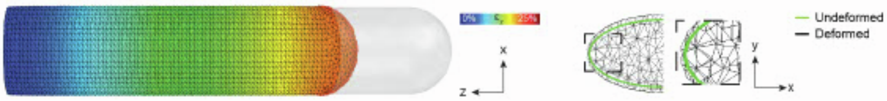

Fig. S14: Loading the FE model with an anisotropic surface tension, related to Figure 3. Simulating a longitudinal surface tension by allowing the shell material to shrink only along the length of the tissue resulted in an anisotropic reduction in length towards the head (left panel) and an increase in both height and width of the tissue (right panel). This result mimicked the experimental observations in the *Drosophila* embryo. Colours represent the magnitude of displacement along the length of the tissue (z-axis) with values ranging from 0% to 25% of strain.

3.4 Comparison

While all three simulations (uniform pressure, isotropic surface tension, and longitudinal surface tension) showed an anisotropic reduction in the length of the tissue from tail to head (Fig. S15), the results when looking at the tissue cross-section varied, with only the longitudinal surface tension scenario predicting the expansion in both height and width of the tissue which was observed experimentally in the *Drosophila* embryo (Fig. S14).

This difference is further highlighted by the vectorial representation of the displacement (Fig. S16): it can be noted how, in the case of the uniform pressure loading, the vectors point towards a central node at the head of the tissue

suggesting a deformation along all axes. For the isotropic surface tension, this behaviour is less pronounced, but can still be observed towards the head showing additional deformation along the cross-section of the tissue. Finally, in the longitudinal surface tension case, the vectors have a tendency to point outwards from the tissue centre which explains the expansion in both x- and y-axis. The FE simulations, therefore, suggested that VNC condensation can be explained by a preferentially directed surface tension.

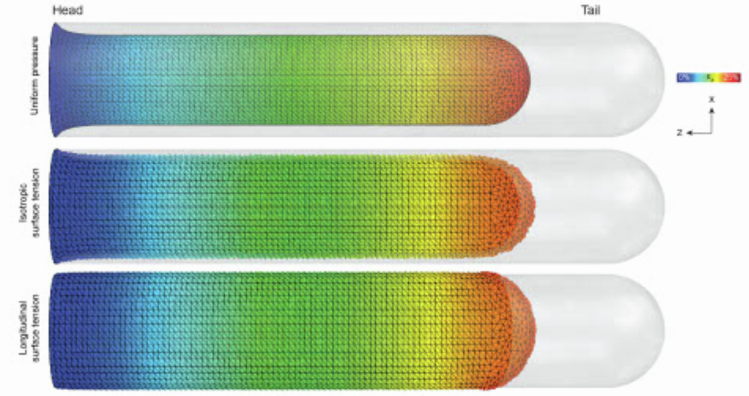

Fig. S15: Comparing the displacement along the length of the tissue in the different loading conditions, related to Figure 3. Loading the model by uniform pressure, isotropic, and longitudinal surface tension resulted in an anisotropic reduction in the VNC length from tail to head. Colours represent the magnitude of displacement along the length of the tissue (z-axis) with values ranging from 0% to 25% of strain.

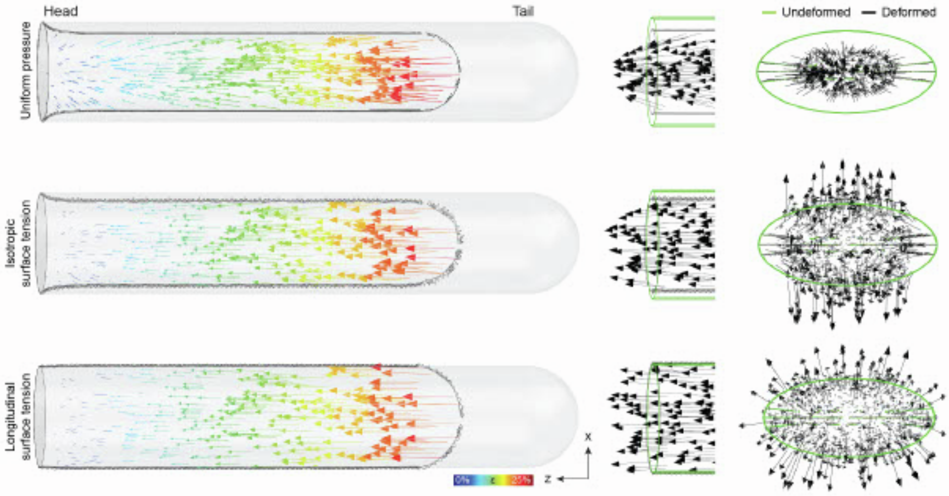

Fig. S16: Comparing the displacement vectors in the different loading conditions, related to Figure 3. Vectorial representation of the displacement of the VNC when comparing loading the model by uniform pressure, isotropic, and longitudinal surface tension (left panels). The representation with unit vectors on a vertical cut along the VNC length highlights the directionality of the displacement (right panels). Colours represent the magnitude of overall displacement with values ranging from 0% to 25% of strain.

4 Disrupting the longitudinal surface tension

We hypothesised that an increase in longitudinal surface tension would drive the tissue reduction in length by global transmission of ECM-generated stresses through the polymer network. We therefore tested the effects of a local disruption in the surface tension on VNC condensation.

To achieve this, we defined the initial temperature field controlling the shell shrinking as a function of the tissue length (z-axis), before turning the temperature to 0 degrees to enable the shell shrinking. If no disruption in surface tension was to be simulated, the temperature value was kept constant along z (top panel Fig. S17, and Fig. S14). Otherwise, we analytically defined the shape of the initial temperature field along z with a Gaussian-shaped drop in temperature around the centre of the VNC (middle and bottom panels Fig. S17). The drop could be modulated to simulate a partial or null surface tension in a stripe of the tissue, by setting the target temperature to 50% (middle panel Fig. S17) or 0% (bottom panel Fig. S17) of the temperature away from the stripe.

Simulations revealed that a local disruption of the surface tension had an effect on the reduction in length of the tissue, proportional to the severity of the disruption (Fig. S17). Similar results were obtained when experimentally causing a local disruption of the collagen network, by expressing surface-bound matrix metalloproteases in a stripe along the VNC (Figure 4B in the main text). These results taken together suggest that surface tension-driven change in tissue shape should involve long-range and coordinated morphological remodelling.

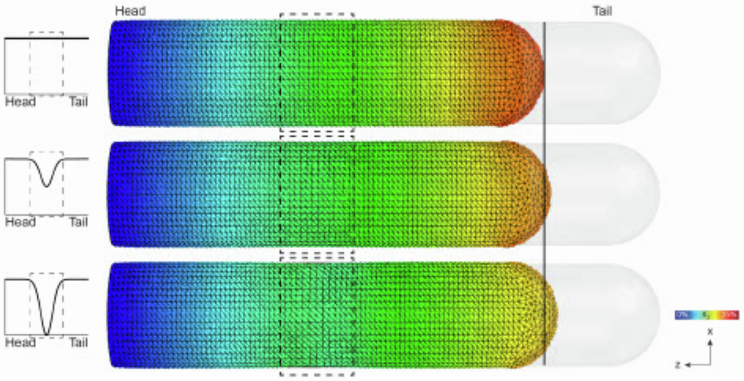

Fig. S17: Modelling the disruption of surface tension, related to Figure 4. The reduction in VNC length was affected by locally disrupting the surface tension (black vertical line). This was achieved by locally modifying the initial temperature field of the simulation with a drop around the middle of the tissue (black squares) of either 50% (middle panel) or 100% (bottom panel), corresponding to halving or deleting the surface tension in a stripe of the VNC, respectively. Colours represent the magnitude of displacement along the length of the tissue (z-axis) with values ranging from 0% to 25% of strain.
