# Supplementary material for: Extracellular matrix assembly stress initiates Drosophila central nervous system morphogenesis
Source: Dev Cell. 2023 May 22;58(10):825–835.e6. doi: 10.1016/j.devcel.2023.03.019 (PMC10390342; doi:10.1016/j.devcel.2023.03.019)
Supplement: Document S2. Article plus supplemental information [file mmc5.pdf]

Short article

Developmental Cell

Extracellular matrix assembly stress initiates *Drosophila* central nervous system morphogenesis

Graphical abstract

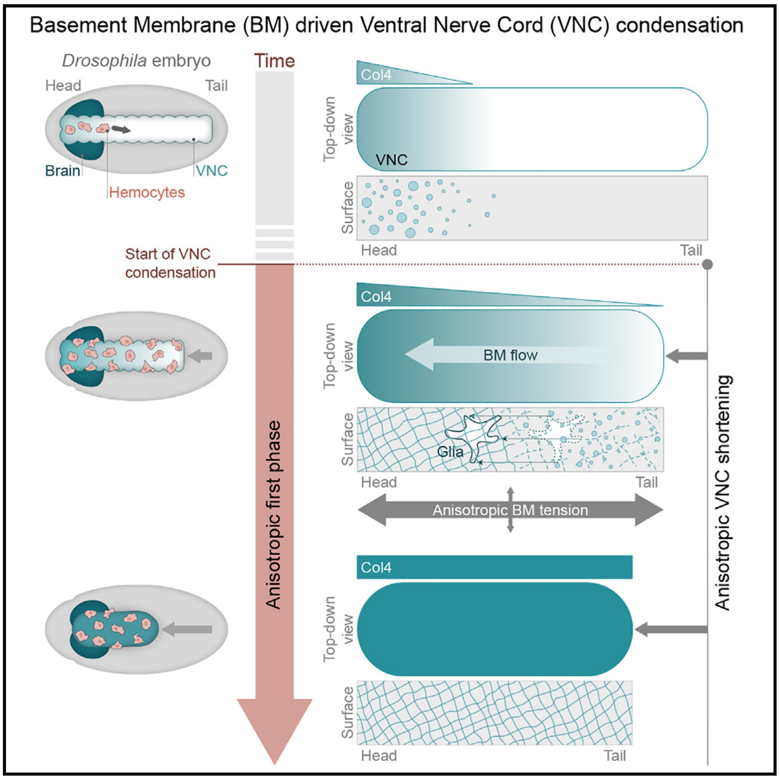

Highlights

- Sudden uneven Col4 assembly surrounding the fly VNC initiates tissue condensation
- Col4 flows coherently on the tissue surface independently of cellular contractility
- Modeling indicates that a BM-dependent increase in surface tension drives morphogenesis
- Perturbations of Col4 assembly affect the rate of VNC condensation

Authors

Eduardo Serna-Morales, Besaiz J. Sánchez-Sánchez, Stefania Marcotti, ..., Chad M. Hobson, Teng-Leong Chew, Brian M. Stramer

Correspondence

brian.m.stramer@kcl.ac.uk

In brief

Serna-Morales et al. demonstrate that the initiation of *Drosophila* VNC condensation is triggered by sudden basement membrane assembly around the tissue, which increases the surface tension independently of cellular contractile activity. Their work suggests that intrinsic stresses in an assembling basement membrane network can actively contribute forces that drive tissue morphogenesis.

Serna-Morales et al., 2023, Developmental Cell *58*, 825–835
May 22, 2023 © 2023 The Author(s). Published by Elsevier Inc.
https://doi.org/10.1016/j.devcel.2023.03.019

CellPress

Developmental Cell

CellPress
OPEN ACCESS

Short article

Extracellular matrix assembly stress initiates *Drosophila* central nervous system morphogenesis

Eduardo Serna-Morales,[1,5] Besaiz J. Sánchez-Sánchez,[1,5] Stefania Marcotti,[1,5] Angus Nichols,[1] Anushka Bhargava,[1] Anca Dragu,[1] Liisa M. Hirvonen,[1] María-del-Carmen Díaz-de-la-Loza,[1] Matyas Mink,[2] Susan Cox,[1] Emily Rayfield,[3] Rachel M. Lee,[4] Chad M. Hobson,[4] Teng-Leong Chew,[4] and Brian M. Stramer[1,6,*]
[1]Randall Centre for Cell and Molecular Biophysics, King's College London, SE1 1UL London, UK
[2]Institute of Medical Biology, University of Szeged, 6720 Szeged, Hungary
[3]School of Earth Sciences, University of Bristol, BS8 1QU Bristol, UK
[4]Advanced Imaging Center, Howard Hughes Medical Institute Janelia Research Campus, Ashburn, VA 20147, USA
[5]These authors contributed equally
[6]Lead contact
*Correspondence: brian.m.stramer@kcl.ac.uk
https://doi.org/10.1016/j.devcel.2023.03.019

SUMMARY

Forces controlling tissue morphogenesis are attributed to cellular-driven activities, and any role for extracellular matrix (ECM) is assumed to be passive. However, all polymer networks, including ECM, can develop autonomous stresses during their assembly. Here, we examine the morphogenetic function of an ECM before reaching homeostatic equilibrium by analyzing *de novo* ECM assembly during *Drosophila* ventral nerve cord (VNC) condensation. Asymmetric VNC shortening and a rapid decrease in surface area correlate with the exponential assembly of collagen IV (Col4) surrounding the tissue. Concomitantly, a transient developmentally induced Col4 gradient leads to coherent long-range flow of ECM, which equilibrates the Col4 network. Finite element analysis and perturbation of Col4 network formation through the generation of dominant Col4 mutations that affect assembly reveal that VNC morphodynamics is partially driven by a sudden increase in ECM-driven surface tension. These data suggest that ECM assembly stress and associated network instabilities can actively participate in tissue morphogenesis.

INTRODUCTION

The forces controlling tissue development are attributed to cellular-driven activities.[1] Any role for extracellular matrix (ECM) in modulating overall tissue shape is largely assumed to be passive by providing a substrate that allows cells to actively remodel their environment.[2] However, recent work on developing zebrafish semicircular canals has revealed that ECM accumulation can have a more instructive role in shaping tissues through swelling and modulation of osmotic pressure.[3] In addition, all polymer networks—including ECM—can theoretically develop autonomous stresses during their initial polymerization, and there has been speculation that out-of-equilibrium ECM behaviors may be playing a more active role during tissue morphogenesis than many would assume.[4–7] Investigating these active mechanisms requires analyzing the ECM network during embryonic assembly before reaching a crosslinked, homeostatic state. Here, we exploit *Drosophila* embryogenesis, which involves a *de novo* burst of ECM polymerization midway through development,[8,9] to examine the role of an out-of-equilibrium ECM network during central nervous system development. Although altering cellular activity failed to stop the initiation of ventral nerve cord (VNC) morphogenesis, we found that perturbing ECM assembly or delivery to the tissue surface severely affected the process. Live imaging of ECM polymerization during morphogenetic initiation identified a sudden coherent flow of matrix on the tissue surface—independent of cellular dynamics—in a posterior to anterior direction, which correlates with the overall motion of the tissue. In addition, finite element modeling identified that an anisotropic surface tension along the axis of ECM motion was sufficient to explain the complex shape changes associated with VNC morphogenesis. These findings show that ECM assembly can actively contribute forces that shape a developing tissue.

RESULTS

Drosophila VNC condensation consists of two distinct temporal phases, and initial anisotropic changes in tissue morphology are independent of VNC cellular activity

During stage 15 of *Drosophila* development, the embryonic nerve cord undergoes a sudden reduction in length in a process called VNC condensation. Time-lapse imaging of the entire ~12 h of condensation revealed two distinct phases in VNC morphodynamics: a rapid 1st phase when most of the morphological remodeling occurs in which the tissue shortens asymmetrically

© 2023 The Author(s). Published by Elsevier Inc.
This is an open access article under the CC BY license (http://creativecommons.org/licenses/by/4.0/).

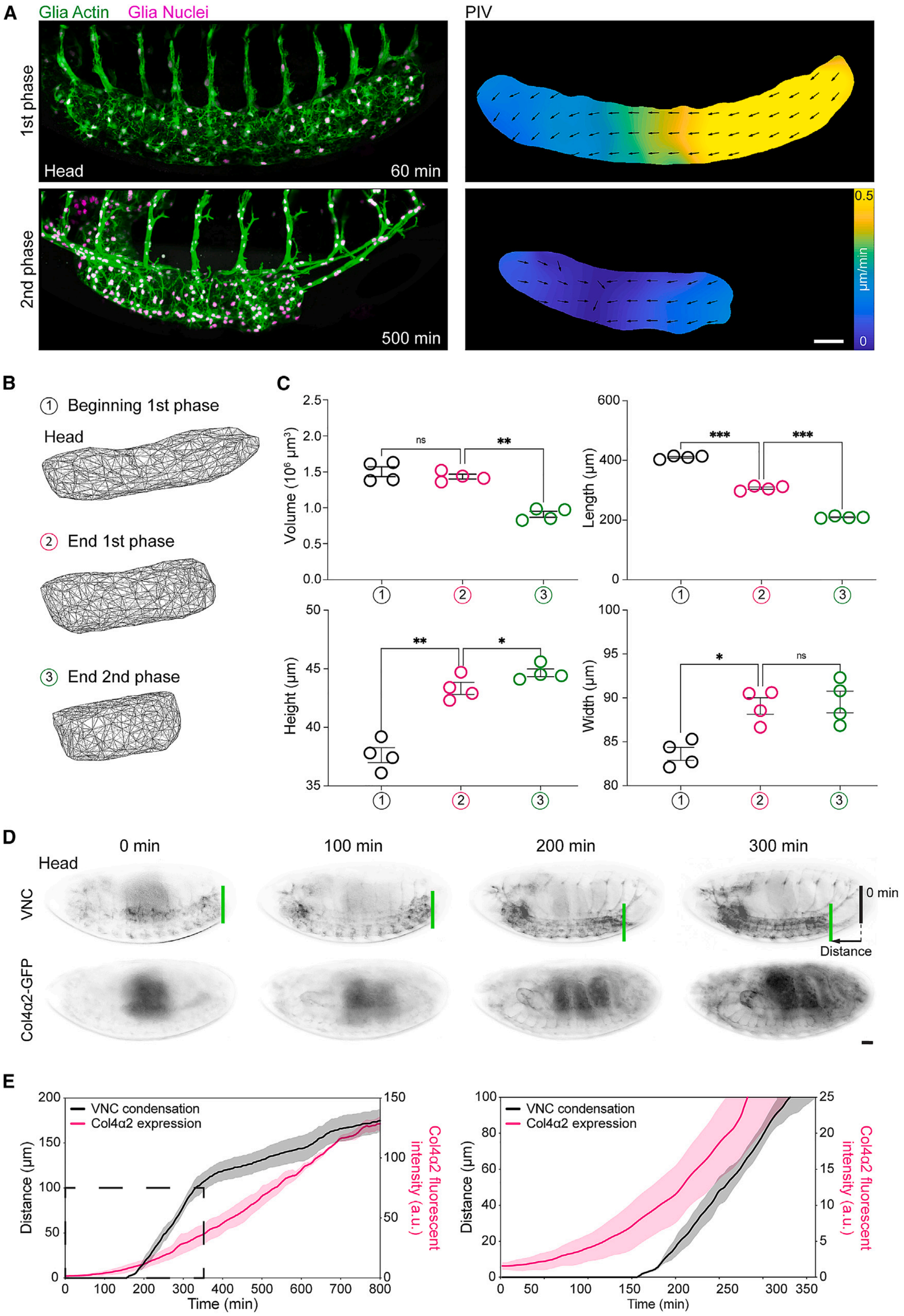

(legend on next page)

from tail to head over ~3 h and a slower 2nd phase resulting in a symmetric reduction in length over the remaining ~9 h of development (Figures 1A, S1A, and S1B; Video S1, parts 1–3). Analyses of tissue geometry revealed that although the 2nd phase involved a reduction in tissue volume, the change in shape during the 1st phase was isovolumetric, suggesting that at least for the 1st phase, the process is not a simple "condensation" (Figures 1B and 1C). Interestingly, it was recently reported that the scaling of VNC length with embryo length is suddenly lost after the start of condensation,[10] which is likely explained by the increase in thickness of the tissue (Figures 1B and 1C). These data suggest that VNC morphogenesis involves temporally controlled and mechanistically distinct processes.

The VNC is composed of a central nerve bundle surrounded by supporting glial cells, and previous work revealed that affecting glial or neuronal activity perturbs condensation.[11] However, inhibiting cell activity by driving a dominant negative (DN) Myosin-II or Rac GTPase in glia or neurons led to relatively minor effects on condensation, whereby the 1st phase was left intact (Figures S1C–S1H). In addition, tracking glial movement during the 1st phase revealed a coherent flow of cells without any cellular rearrangements that could explain the VNC morphodynamics (Figures S1I–S1L; Video S1, part 4). Consistent with these results, recent work revealed that the genetic ablation of glia or neurons and RNAi knockdown of Myosin-II in glia left the initial fast phase of VNC condensation intact[12] (Figure S1D). However, unlike what was previously reported, we did not observe any oscillatory behavior during the 1st phase of condensation.[12] Instead, we observed a highly coordinated anisotropic movement of VNC segments across the tissue rather than independently contracting elements. These data suggest that there are VNC cell-extrinsic forces driving the 1st phase of condensation, and we therefore set out to determine what was initiating the process.

Initiation of VNC condensation is correlated with hemocyte deposition of Col4 on the tissue surface and a sudden increase in tissue stiffness

There is an underexplored function of the ECM during *Drosophila* VNC morphogenesis. The VNC is ensheathed by a basement membrane (BM) composed of collagen type IV (Col4) and Laminin, and mutations in BM components perturb condensation.[13,14] Correlation of VNC condensation with Col4 levels using a GFP-trap in the Col4α2 subunit revealed that the initiation of the process coincided with the embryonic induction of Col4 expression and an exponential increase in production (Figures 1D and 1E; Video S1, parts 5 and 6). By contrast, Laminin is produced at much earlier stages of embryogenesis,[8,9] which led us to hypothesize that Col4 assembly was specifically involved in triggering the start of VNC morphogenesis.

Drosophila hemocytes (macrophages), which are also hypothesized to be involved in VNC condensation,[9,11] are the major producers of Col4 during embryogenesis[9] and their developmental dispersal from their birth in the head mesoderm coincides with the start of condensation (Video S1, part 7). Hemocytes deposit Col4 around the VNC as they disperse evenly throughout the embryo,[9] which consequently results in a transient gradient of Col4 from head to tail along the tissue (Figures S2A and S2B). Interestingly, this is consistent with a recently observed head-to-tail gradient in VNC stiffness[12] along with data in *Drosophila* egg chambers revealing that graded Col4 assembly translates into graded BM stiffness.[15] This suggests that local differences in Col4 assembly can indeed lead to a mechanical gradient across the VNC surface. Subsequently, as condensation proceeds, the Col4 gradient equilibrated such that by the start of the 2nd phase, there was an even distribution of Col4 around the VNC (Figures S2A and S2B). Col4 induction also correlated with an increase in VNC stiffness from the 1st to the 2nd phase of condensation, consistent with data revealing that BM stiffness mainly depends on Col4[16] (Figure 2A). These data suggest that Col4 assembly was suddenly altering the mechanical properties of the tissue.

Inhibiting Col4 deposition or altering its distribution along the VNC surface prevents the initial anisotropic change in VNC morphology

Analyses of BM mutants revealed that in the absence of Laminin, which is required for subsequent BM component assembly,[9] condensation is severely affected and the VNC falls apart due to cell clumping (Figures 2B–2D; Video S2, part 1). By contrast, in the absence of Perlecan, which is expressed slightly later during embryogenesis than Col4,[9] condensation was slower and the 2nd phase was severely affected; however, there was a clear asymmetric 1st phase of VNC morphogenesis (Figures 2B–2D; Video S2, part 1). However, Col4 mutants had a distinct phenotype: Col4 mutant VNCs remained intact, yet deformed, and both phases of VNC condensation were severely inhibited (Figures 2B–2D; Video S2, part 1). Importantly, the 1st phase of condensation was lost in the absence of Col4 and what little condensation was present was isotropic rather than asymmetric (Figures 2C and 2D; Video S2, part 1). In addition, Col4 mutants

Figure 1. VNC morphogenesis involves distinct stages and correlates with the initiation of Col4 assembly

(A) Live imaging of VNC morphogenesis (left panels) and quantification of tissue motion by PIV (right panels) revealing anisotropic (1st phase) and isotropic (2nd phase) phases of condensation. Scale bar, 30 μm.

(B) 3D reconstruction of VNC shape during the phases of condensation.

(C) Quantification of VNC shape at the start (1), end of the 1st phase (2) and end of the 2nd phase (3) of condensation. Repeated measures one-way ANOVA with Geisser-Greenhouse correction and Holm-Šídák's multiple comparisons tests. Each dot represents one embryo, n= 4 embryos. Error bars show standard error of the mean. Volume: ns p = 0.4130, **p = 0.0073; Length: ***p = 0.0004; Height: **p = 0.0045, *p = 0.0155; Width: *p = 0.0278, ns p = 0.6803.

(D) Live imaging of VNC condensation (highlighted by the difference between the green and black lines) and induction of Col4 production by quantifying fluorescence intensity of Col4α2-GFP. Scale bar, 30 μm.

(E) Correlation of Col4 fluorescence intensity with the rate of VNC condensation as measured by tracking the motion of the tail of the tissue. Right panel focuses on the data within the dashed square. n = 3 embryos.

See also Video S1.

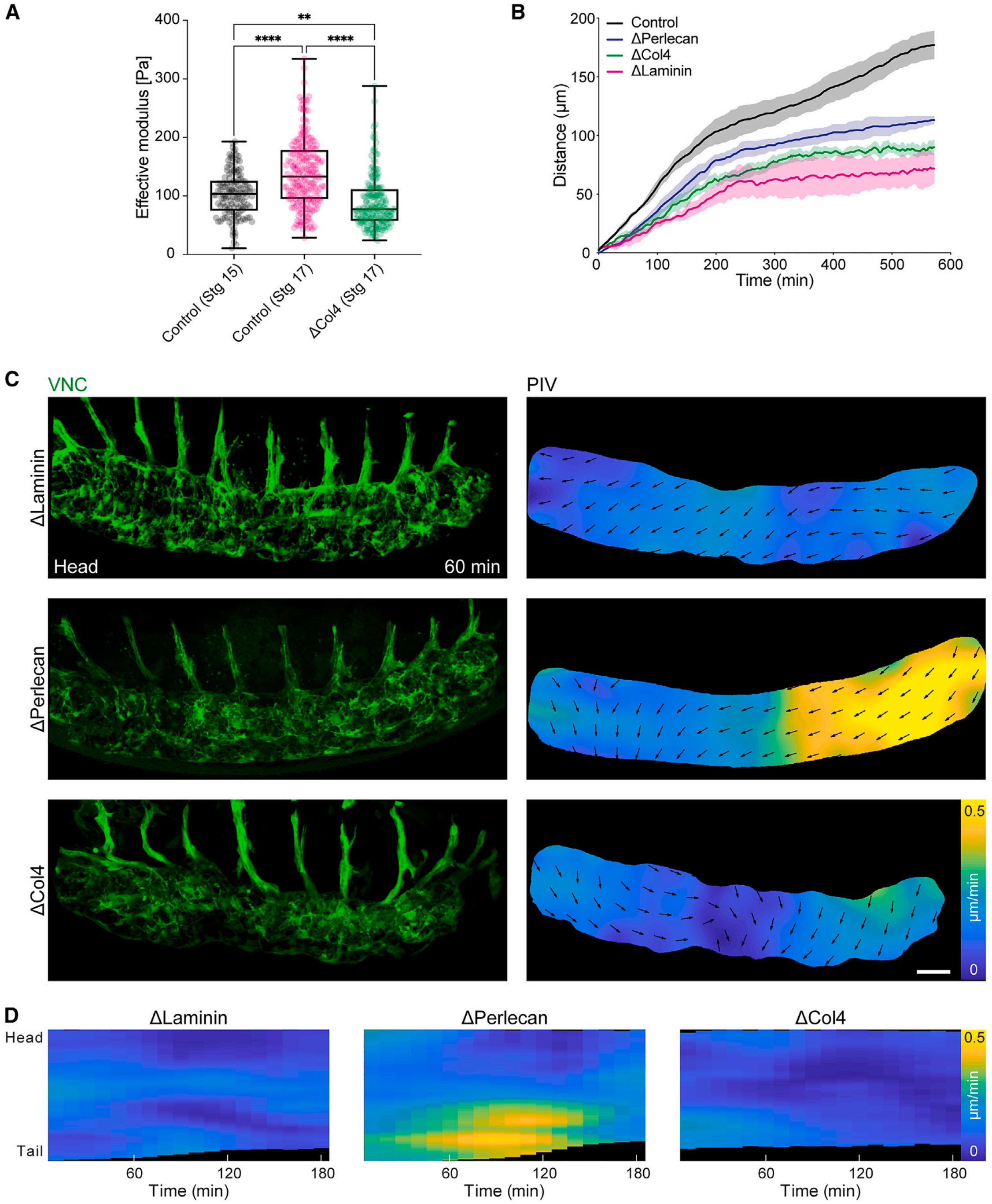

Figure 2. Col4 induction correlates with an increase in VNC stiffness, and the loss of Col4 inhibits the anisotropic phase of VNC condensation
(A) Atomic force microscopy of the VNC revealing an increase in stiffness from stage 15 to 17 (1st and 2nd phases, respectively), which is lost in the absence of Col4. Kruskal-Wallis tests and Dunn's multiple comparisons tests. n = 137 indentations (one control embryo, stg 15), n = 207 (two control embryos, stg 17), n = 181 (two ΔCol4 embryos, stg 17). Each dot represents one indentation. Boxplots show medians, 25th and 75th percentiles as box limits, 10th and 90th percentiles as whiskers. ****$p < 0.0001$, **$p = 0.0018$.
(B) Quantification of VNC condensation rate by tracking tail motion as in Figures 1D and 1E in control (n = 4 embryos) and *laminin*, *perlecan*, and *col4* mutant embryos (n = 3 embryos each).
(C) Live imaging of the 1st phase of VNC condensation as in Figure 1A in *laminin*, *perlecan*, and *col4* mutants. Scale bar, 30 μm.
(D) Kymograph of the average speed of VNC condensation from PIV analysis in (C) highlighting an absence of an anisotropic phase of condensation in *laminin* and *col4* mutants.
See also Video S2.

CellPress
OPEN ACCESS

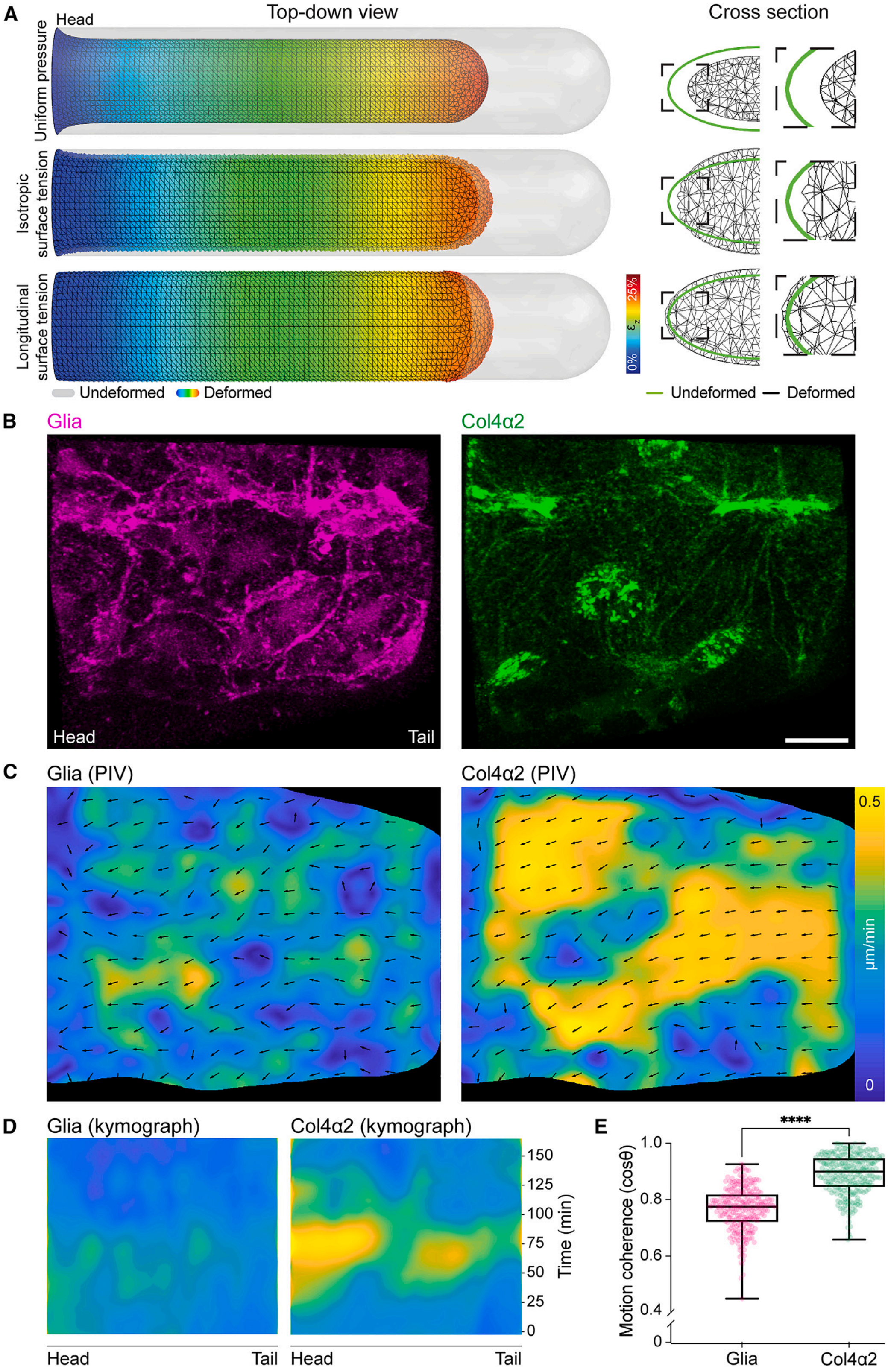

(legend on next page)

failed to show an increase in tissue stiffness (Figure 2A). The Col4 mutant VNC phenotype was specific to Col4 assembly around the tissue as local disruption of Col4 by expression of a surface-bound matrix metalloprotease (*Drosophila* MMP2)[17,18] with the same glial-Gal4 driver used to inhibit glial cell activity, phenocopied the Col4 mutant (Figures S2C and S2D). Genetically deleting hemocytes, a major source of embryonic Col4, also reduced VNC shortening (Figure S3). Furthermore, preventing hemocyte release from the head of the embryo by expression of Rac DN, which causes a relative overabundance of Col4 in the head and an exacerbation of the Col4 gradient[9] led to an almost complete cessation of VNC condensation and abnormal deformation of the tissue (Figure S3; Video S2, parts 2 and 3). Therefore, similar to what was previously observed,[11,19] disrupting hemocytes and Col4 distribution leads to a far more severe effect on VNC condensation than inhibiting VNC cellular activity.

Finite element analysis reveals that the initial anisotropic change in VNC morphology can be explained by a sudden increase in asymmetric surface tension

Polymer networks, especially in thin film organization, are known to generate stresses during assembly, which can lead to polymer motion as stresses equilibrate.[20] Indeed, inhomogeneities in mixtures of ECM components in a test tube can generate forces that directly drive long-range translocation of inert particles.[5,6] To determine whether BM-derived surface stress could contribute to the observed changes in VNC shape on the initiation of condensation, we developed a finite element model consisting of two materials: an internal core representing the VNC attached to the brain, which is surrounded by a thin BM shell. Simulations revealed that a uniform pressure perpendicular to the surface was sufficient to decrease the length of the tissue from tail to head. However, this also led to the constriction of the diameter of the VNC, which was not observed experimentally during the 1st phase of condensation (Figure 3A; Methods S1). In a second approach, we modeled the shell such that it instead exerted a surface tension, which can be modulated by altering its capacity to shrink and its stiffness. Consistent with the Col4-dependent increase in tissue stiffness during condensation, a simulated isotropic increase in surface tension asymmetrically reduced the tissue length; however, this also led to a reduction in tissue width and rounding of nerve cords, which was also not observed in real specimens (Figures 1B, 1C, and 3A; Methods S1). However, further simulations suggested that an anisotropic surface tension, which is predominantly along the length of the tissue, could explain the increase in width and height of the VNC during the 1st phase of condensation (Figures 1B, 1C, and 3A; Methods S1). These data suggest that a BM-dependent increase in tissue surface tension predominantly along the axis of the previously observed stiffness gradient[12] (and Col4 gradient, this work) can explain the rapid anisotropic change in tissue shape during the initiation of VNC morphogenesis.

Live imaging of Col4 assembly on the VNC surface reveals a coherent viscous-like flow along the axis of the predicted increase in tissue surface tension

We subsequently live-imaged surface glial dynamics and Col4 accumulation surrounding the VNC at a high spatiotemporal resolution to determine the possible interplay between cell activity and ECM remodeling. As VNC condensation initiated, a rapid and coherent long-range flow of Col4 was observed—preceding glial motion—in a direction antiparallel to the Col4 gradient (i.e., tail to head direction), which was along the predicted predominant axis of surface tension (Figures 3B–3D and S4A–S4E; Video S3, parts 1–3). Subsequently, there was a drift in the surface glia, which are in direct contact with the overlying ECM enveloping the tissue (Figures 3B and 3C; Video S3, parts 2 and 3). At the start of condensation, the surface glia are initially spaced out on the VNC surface and not in contact with each other and eventually undergo a mesenchymal to epithelial transition to form an epithelial monolayer by the end of the 1st phase.[21,22] Despite the approximate 16% decrease in VNC surface area during the 1st phase (determined from our measured change in tissue dimensions, Figures 1B and 1C), surface glial cells paradoxically increase in area as they spread themselves around the tissue,[21,22] highlighting that glial cell contraction is not involved in this change in tissue geometry. We observed no obvious cell migration or cell rearrangements of glial cells that would explain the overall change in VNC morphology nor did we observe any local ECM remodeling by the underlying glial cells (Video S3, parts 2 and 3). Indeed, correlating the local alignment of particle image velocimetry (PIV) vectors of ECM and glial motion revealed that the Col4 network was moving more coherently over the tissue surface than the cells themselves (Figures 3D and 3E). In addition, the Col4 network appeared to self-assemble with polymers stretching tens of microns linking the dispersed population of glial cells (Figure 3B). Therefore, as predicted of non-uniform thin film growth with a strain or structural gradient,[20] these data reveal that BM-autonomous assembly dynamics lead to long-range viscous flow and equilibration of the network.

Figure 3. Anisotropy in surface tension and coherent long-range flow of Col4 is sufficient to explain the sudden isovolumetric change in VNC shape

(A) Finite element analysis (FEA) of VNC morphogenesis. Simulations of VNC deformation assuming (top panel) uniform normal surface pressure, (middle panel) uniform increase in surface tension, or (bottom panel) anisotropic increase in surface tension along the length of the tissue. Only anisotropic surface tension leads to reduction in length and increase in height and width of the tissue.

(B) Live imaging of glia and Col4 motion on the VNC surface. Scale bar, 10 μm.

(C) Simultaneous tracking of glia and Col4 motion by PIV.

(D) Kymograph of the PIV analysis in (C) highlighting a sudden increase in Col4 speed during VNC condensation which is not observed by tracking glia.

(E) Correlation of local alignment of PIV vectors reveals that motion of the Col4 network is more coherent than glial motion. Mann-Whitney test. n = 256 vectors each. Each dot represents one PIV vector. Boxplots show medians, 25th and 75th percentiles as box limits, 10th and 90th percentiles as whiskers. ****$p < 0.0001$. See also Video S3.

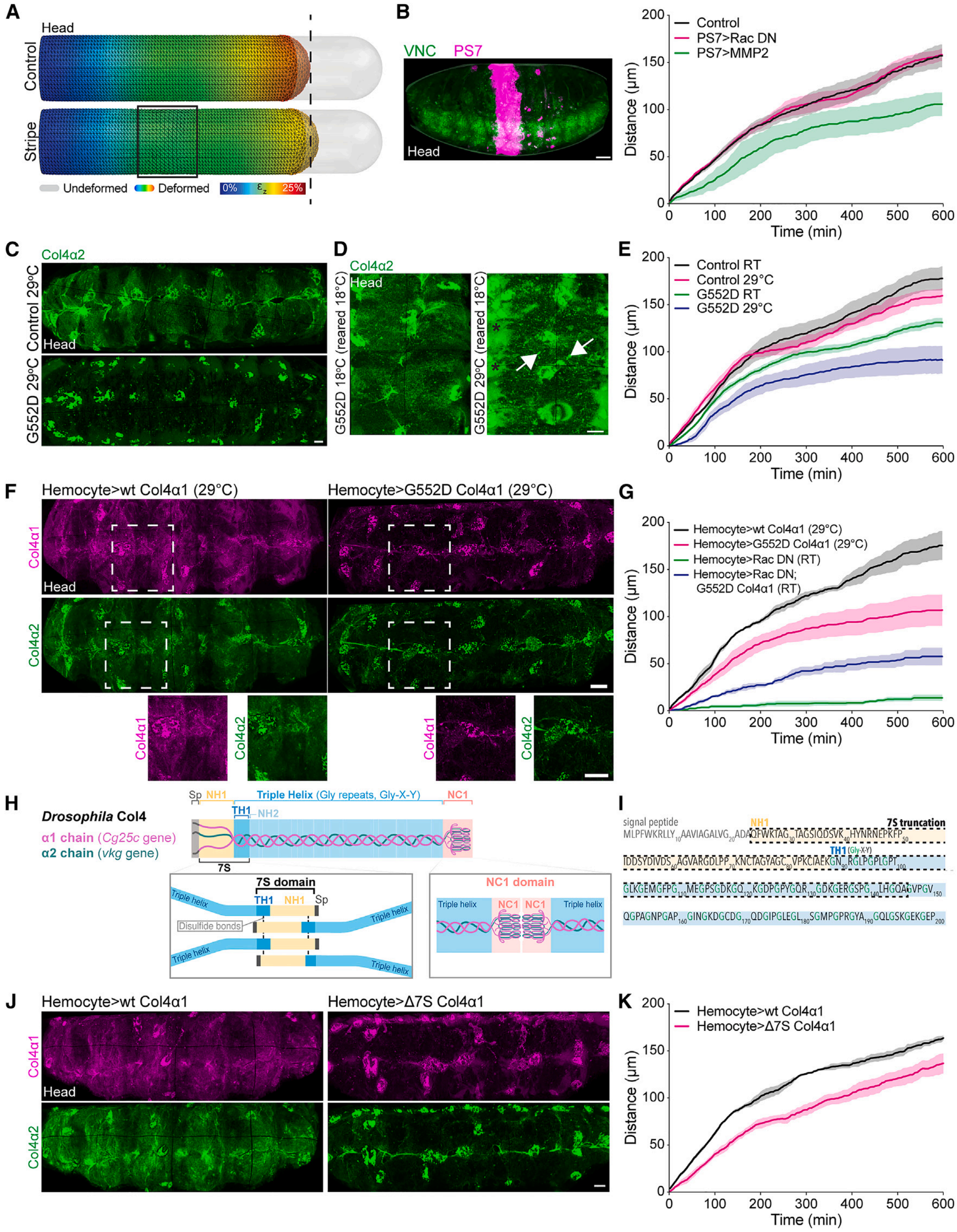

Figure 4. Direct perturbations of Col4 assembly slow the rate of VNC condensation

(A) FEA of VNC condensation in which surface tension is locally reduced in a stripe in the middle of the tissue. Simulations suggest that local perturbation of surface tension leads to long-range reduction in deformation of the tail of the tissue.

(B) Local disruption of the ECM network by MMP2 expression in a central parasegment (PS7, n = 4), in contrast to control (n = 3) and expression of Rac DN (n = 3), is sufficient to affect the rate of VNC condensation as measured by tracking the tail of the tissue as in Figures 1D and 1E. Scale bar, 30 μm.

(C) Live imaging of Col4α2-GFP reveals that rearing embryos containing a temperature-sensitive (TS) point mutation in Col4α1 (G552D) at the non-permissive temperature (29°C) inhibits BM network assembly. Scale bar, 10 μm.

(legend continued on next page)

Local disruption of the BM network or perturbation of Col4 assembly alters VNC morphodynamics

We next examined perturbations that affect ECM polymerization or network organization on VNC morphodynamics. First, modeling predicted that an increase in uniform surface tension should lead to rounder nerve cords when viewed in cross-section, which was observed in Col4 mutants (Figures S4F and S4G). This suggests that in the absence of Col4 assembly, ensheathment of the VNC by surface glia, which are hypothesized to act as a "compression sock" around the tissue,[12] results in isotropic surface tension that alters VNC morphology. Simulations further suggested that local changes in surface tension should lead to global effects on condensation rate (Figure 4A; Methods S1). To confirm the presence of long-range network effects, we locally cleaved the ECM network in a narrow stripe along the VNC using a parasegmental driver[23] with MMP2 expression[17,18] and examined VNC motion by tracking the tail of the tissue. Cleaving the ECM network in a stripe in the middle of the VNC slowed the motion of the tail of the tissue, which was over a hundred microns distant to the site of cleavage (Figure 4B). This long-range effect suggests that a coherent and interconnected ECM network is required for VNC morphogenesis. We subsequently examined a temperature-sensitive (TS) point mutant Col4 allele (G552D)[24,25] that resulted in the ECM network disintegrating when shifted to 29°C (Figures 4C and 4D). This revealed that subtle alteration of the temperature in this TS mutant was sufficient to affect the overall rate of VNC motion (Figure 4E). In addition, driving a G552D Col4 transgene specifically in hemocytes severely inhibited VNC condensation, phenocopying the Col4 mutant, confirming that hemocyte-derived Col4 is indeed essential for initiating the process (Figures 4F and 4G). In addition, expressing this same DN Col4 transgene (G552D) while also trapping hemocytes in the head partially rescued the severe condensation defect, suggesting that uneven Col4 assembly was causing this severe phenotype (Figure 4G; Video S2, part 3). We also generated an N-terminal truncation in the Col4α1 protein that removed part of the putative 7S domain (Figures 4H and 4I), which is hypothesized to be involved in multivalent interactions important for Col4 network assembly.[26,27] Driving this transgene specifically in hemocytes was sufficient to perturb Col4 network formation and subtly affect condensation rate, which we speculate is due to a retardation of Col4 polymerization (Figures 4J and 4K).

DISCUSSION

The textbook view of morphogenesis has assumed that the changes in tissue shape are driven by a limited repertoire of cell-autonomous activities (e.g., cell rearrangements, shape changes, migrations, and divisions).[1] However, the supramolecular nature of ECM networks, such as BMs, that rely on underexplored covalent and noncovalent interactions for their self-assembly[8,28–31] should not be neglected as sources of stress that shape an embryo. In addition, surface tension has been predicted to be a dominant force in shaping developing tissues,[32] and although alterations in surface tension are assumed to be driven by epithelial activity,[33] BM networks are also likely to be involved. Indeed, in the case of VNC condensation, the sudden exponential increase in Col4 formation around the VNC is behaving as a compression sleeve, which actively envelops the tissue to shrink its surface area, resulting in an initial isovolumetric shape change as surface stresses equilibrate. By contrast, in the 2nd phase of condensation, once the asymmetric Col4 network stresses have dissipated, the cells within the VNC likely take a more active part in the process with the ECM possibly playing a more stereotypical passive role.

There are many reasons why a polymer network such as the BM could generate forces during development. The polymerization process on its own, especially if there are instabilities such as concentration gradients during assembly, will lead to stress development and ECM-autonomous motion. Indeed, *in vitro* polymerization assays have revealed that non-uniform distributions of collagen self-assembly can generate surface tension gradients that lead to a Marangoni flow within the network.[34] In addition, a mechanical mismatch between a polymer thin film

(D) Rearing the TS mutant at the permissive temperature (18°C) to allow some ECM assembly and switching to the non-permissive temperature (29°C) leads to aggregation of extracellular Col4 (arrows) and accumulation of soluble Col4 in the hemocoel (asterisks) showing that the TS mutant affects the Col4 network. Scale bar, 10 μm.

(E) Quantification of VNC condensation in the TS mutant reveals that the condensation rate can be affected by temperature titration (RT, room temperature). Control data reused from Figure 2B. n = 4 control RT and 29°C, n = 5 G552D RT and 29°C.

(F) Expression of wild-type or G552D point mutant Col4α1 in hemocytes during VNC condensation in the background of endogenously GFP-tagged Col4α2. Bottom panels are high-magnification views of the highlighted regions revealing that the G552D transgene fails to incorporate into the ECM network and inhibits incorporation of Col4α2. Scale bars, 10 μm.

(G) Quantification of VNC condensation in the genotypes highlighted in (F) reveals that hemocyte-specific expression of the G552D transgene slows VNC condensation. Furthermore, expression of the G552D mutant in hemocytes that also express Rac DN is sufficient to rescue the severe phenotype. n = 3 Hemocyte>wt Col4α1, Hemocyte>Rac DN; G552D Col4α1, n = 5 Hemocyte>G552D Col4α1; Hemocyte>Rac DN is from Figure S3C.

(H) Schematic highlighting Col4 domains and interactions hypothesized to drive network assembly. In the C terminus of Col4 are interactions between NC1 domains, which lead to dimerization of Col4 trimers. At the N terminus are interactions of a putative 7S domain, which consists of the first non-helical (NH1) and triple-helical (TH1) regions of the protein. Disulfide bonds and noncovalent interactions between 7S domains are hypothesized to lead to tetramerization of Col4 trimers. Sp, signal peptide.

(I) Amino acid sequence of the N terminus of *Drosophila* Col4. The dashed line highlights the amino acid sequence truncated from the putative 7S domain of Col4α1 to generate a dominant negative transgene.

(J) Expression of wild-type (WT) Col4α1 or a truncation of the 7S domain in hemocytes shows that deletion of the 7S domain decreases its incorporation and reduces incorporation of Col4α2. Scale bar, 10 μm.

(K) Quantification of VNC condensation after expression of the transgenes in (H) reveals that removal of the 7S domain slows the rate of motion of the tail of the tissue. n = 4 embryos each.

See also Video S2.

CellPress
OPEN ACCESS

(i.e., BM), and the underlying substrate can also lead to material deformations.[20,35] As long-range coherent ECM motion has been observed during several developmental processes,[4,7,36] nonequilibrium phenomena in an immature ECM network in flux may be playing unappreciated active roles in tissue morphogenesis.

Limitations of the study

Although our data suggest an active role for ECM assembly in generating VNC stresses that initiate morphogenesis, it is difficult to completely rule out any role for cellular activity. Nevertheless, perturbations directly affecting cells within the VNC lead to relatively minor effects and normal initiation of condensation.[11,12] Conversely, BM defects are far more severe. Understanding how intrinsic stresses develop in the embryonic BM is non-trivial. It requires a biophysical dissection of nucleation/growth of the polymer network and polymer-substrate interactions, which is currently beyond the resolution limit of *in vivo* imaging. Our work has also left out the remainder of VNC condensation after the initial fast phase, which likely involves glia working in conjunction with the ECM, and it will be interesting to understand their interplay during this cell-dependent phase of the process.

STAR★METHODS

Detailed methods are provided in the online version of this paper and include the following:

- KEY RESOURCES TABLE
- RESOURCE AVAILABILITY
  - Lead contact
  - Materials availability
  - Data and code availability
- EXPERIMENTAL MODEL AND SUBJECT DETAILS
  - Fly stocks and preparations
- METHOD DETAILS
  - Construction of UAS-LifeAct-mScarlet, UAS- Col4a1^{wt}-mScarlet, UAS- Col4a1^{D7s}-mScarlet and UAS-Col4a1^{G552D}-mScarlet
  - Sample preparation and mounting for imaging
  - Widefield and confocal microscopy
  - Lattice light-sheet microscopy and patterned photobleaching
  - Atomic force microscopy (AFM)
- QUANTIFICATION AND STATISTICAL ANALYSIS
  - Lattice light-sheet imaging pre-processing
  - VNC condensation and Col4 levels quantification by fluorescent dissection microscope
  - Volume and surface measurements
  - Quantification of fluorescence intensity in the surface of the VNC
  - Particle image velocimetry (PIV) analysis
  - PIV local alignment correlation
  - Cell tracking
  - Analysis of photobleaching
  - Eccentricity of VNC cross-sections
  - AFM analysis
  - Statistics and reproducibility

SUPPLEMENTAL INFORMATION

Supplemental information can be found online at https://doi.org/10.1016/j.devcel.2023.03.019.

ACKNOWLEDGMENTS

We would like to thank Nir Gov, Rachel Lennon, Roberto Mayor, Patrick Mesquida, and David Sherwood for advice on the manuscript and Welcome Bender for the parasegmental driver. This research was funded by the Wellcome Trust (grant number 107859/Z/15/Z) (B.M.S. and B.J.S.-S.), the European Research Council (ERC) under the European Union's Horizon 2020 research and innovation program (grant agreement no. 681808) (B.M.S. and S.M.), and the Consejo Nacional de Ciencia y Tecnología (CONACYT), México (E.S.-M.). The Advanced Imaging Center at Janelia Research Campus is supported by the Howard Hughes Medical Institute and the Gordon and Betty Moore Foundation. For the purpose of open access, the author has applied a CC BY public copyright license to any Author Accepted Manuscript version arising from this submission.

AUTHOR CONTRIBUTIONS

Conceptualization, E.S.-M., B.J.S.-S., S.M., and B.M.S.; methodology, E.S.-M., B.J.S.-S., S.M., M.M., B.M.S., C.M.H., R.M.L., and T.-L.C.; investigation, E.S.-M., B.J.S.-S., S.M., A.B., A.D., L.M.H., M.-d.-C.D.-d.-l.-L., M.M., S.C., E.R., B.M.S., C.M.H., and R.M.L.; funding acquisition, B.M.S. and T.-L.C.; project administration, E.S.-M. and B.M.S.; writing – original draft, B.M.S.; writing – review & editing, E.S.-M., B.J.S.-S., S.M., and B.M.S.

DECLARATION OF INTERESTS

The authors declare no competing interests.

Received: March 3, 2022
Revised: December 12, 2022
Accepted: March 5, 2023
Published: April 21, 2023

REFERENCES

1. Gilbert, S.F. (2000). Developmental Biology, Sixth Edition (Sinauer Associates).
2. Walma, D.A.C., and Yamada, K.M. (2020). The extracellular matrix in development. Development *147*, dev175596. https://doi.org/10.1242/dev.175596.
3. Munjal, A., Hannezo, E., Tsai, T.Y., Mitchison, T.J., and Megason, S.G. (2021). Extracellular hyaluronate pressure shaped by cellular tethers drives tissue morphogenesis. Cell *184*, 6313–6325.e18. https://doi.org/10.1016/j.cell.2021.11.025.
4. Loganathan, R., Rongish, B.J., Smith, C.M., Filla, M.B., Czirok, A., Bénazéraf, B., and Little, C.D. (2016). Extracellular matrix motion and early morphogenesis. Development *143*, 2056–2065. https://doi.org/10.1242/dev.127886.
5. Newman, S.A., Frenz, D.A., Tomasek, J.J., and Rabuzzi, D.D. (1985). Matrix-driven translocation of cells and nonliving particles. Science *228*, 885–889. https://doi.org/10.1126/science.4001925.
6. Newman, S.A., Frenz, D.A., Hasegawa, E., and Akiyama, S.K. (1987). Matrix-driven translocation: dependence on interaction of amino-terminal domain of fibronectin with heparin-like surface components of cells or particles. Proc. Natl. Acad. Sci. USA *84*, 4791–4795. https://doi.org/10.1073/pnas.84.14.4791.
7. Zamir, E.A., Rongish, B.J., and Little, C.D. (2008). The ECM moves during primitive streak formation–computation of ECM versus cellular motion. PLoS Biol. *6*, e247. https://doi.org/10.1371/journal.pbio.0060247.
8. Matsubayashi, Y., Sánchez-Sánchez, B.J., Marcotti, S., Serna-Morales, E., Dragu, A., Díaz-de-la-Loza, M.D., Vizcay-Barrena, G., Fleck, R.A., and Stramer, B.M. (2020). Rapid homeostatic turnover of embryonic

ECM during tissue morphogenesis. Dev. Cell *54*, 33–42.e9. https://doi.org/10.1016/j.devcel.2020.06.005.

9. Matsubayashi, Y., Louani, A., Dragu, A., Sánchez-Sánchez, B.J., Serna-Morales, E., Yolland, L., Gyoergy, A., Vizcay, G., Fleck, R.A., Heddleston, J.M., et al. (2017). A moving source of matrix components is essential for de novo basement membrane formation. Curr. Biol. *27*, 3526–3534.e4. https://doi.org/10.1016/j.cub.2017.10.001.
10. Tiwari, P., Rengarajan, H., and Saunders, T.E. (2021). Scaling of internal organs during drosophila embryonic development. Biophys. J. *120*, 4264–4276. https://doi.org/10.1016/j.bpj.2021.05.023.
11. Olofsson, B., and Page, D.T. (2005). Condensation of the central nervous system in embryonic Drosophila is inhibited by blocking hemocyte migration or neural activity. Dev. Biol. *279*, 233–243. https://doi.org/10.1016/j.ydbio.2004.12.020.
12. Karkali, K., Tiwari, P., Singh, A., Tlili, S., Jorba, I., Navajas, D., Muñoz, J.J., Saunders, T.E., and Martin-Blanco, E. (2022). Condensation of the Drosophila nerve cord is oscillatory and depends on coordinated mechanical interactions. Dev. Cell *57*, 867–882.e5. https://doi.org/10.1016/j.devcel.2022.03.007.
13. Borchiellini, C., Coulon, J., and Le Parco, Y. (1996). The function of type IV collagen during Drosophila muscle development. Mech. Dev. *58*, 179–191. https://doi.org/10.1016/s0925-4773(96)00574-6.
14. Urbano, J.M., Torgler, C.N., Molnar, C., Tepass, U., López-Varea, A., Brown, N.H., de Celis, J.F., and Martín-Bermudo, M.D. (2009). Drosophila laminins act as key regulators of basement membrane assembly and morphogenesis. Development *136*, 4165–4176. https://doi.org/10.1242/dev.044263.
15. Crest, J., Diz-Muñoz, A., Chen, D.Y., Fletcher, D.A., and Bilder, D. (2017). Organ sculpting by patterned extracellular matrix stiffness. eLife *6*, e24958. https://doi.org/10.7554/eLife.24958.
16. Töpfer, U., Guerra Santillán, K.Y., Fischer-Friedrich, E., and Dahmann, C. (2022). Distinct contributions of ECM proteins to basement membrane mechanical properties in Drosophila. Development *149*, dev200456. https://doi.org/10.1242/dev.200456.
17. Pastor-Pareja, J.C., Wu, M., and Xu, T. (2008). An innate immune response of blood cells to tumors and tissue damage in Drosophila. discussion 153. Dis. Model. Mech. *1*, 144–154. https://doi.org/10.1242/dmm.000950.
18. Sui, L., Alt, S., Weigert, M., Dye, N., Eaton, S., Jug, F., Myers, E.W., Jülicher, F., Salbreux, G., and Dahmann, C. (2018). Differential lateral and basal tension drive folding of Drosophila wing discs through two distinct mechanisms. Nat. Commun. *9*, 4620. https://doi.org/10.1038/s41467-018-06497-3.
19. Page, D.T., and Olofsson, B. (2008). Multiple roles for apoptosis facilitating condensation of the Drosophila ventral nerve cord. Genesis *46*, 61–68. https://doi.org/10.1002/dvg.20365.
20. Freund, L.B., and Suresh, S. (2009). Thin Film Materials: Stress, Defect Formation, and Surface Evolution, 1st Pbk. Edition (Cambridge University Press).
21. Schwabe, T., Bainton, R.J., Fetter, R.D., Heberlein, U., and Gaul, U. (2005). GPCR signaling is required for blood-brain barrier formation in drosophila. Cell *123*, 133–144. https://doi.org/10.1016/j.cell.2005.08.037.
22. Schwabe, T., Li, X., and Gaul, U. (2017). Dynamic analysis of the mesenchymal-epithelial transition of blood-brain barrier forming glia in Drosophila. Biol. Open *6*, 232–243. https://doi.org/10.1242/bio.020669.
23. Bowman, S.K., Deaton, A.M., Domingues, H., Wang, P.I., Sadreyev, R.I., Kingston, R.E., and Bender, W. (2014). H3K27 modifications define segmental regulatory domains in the Drosophila bithorax complex. eLife *3*, e02833. https://doi.org/10.7554/eLife.02833.
24. Kelemen-Valkony, I., Kiss, M., Csiha, J., Kiss, A., Bircher, U., Szidonya, J., Maróy, P., Juhász, G., Komonyi, O., Csiszár, K., et al. (2012). Drosophila basement membrane collagen col4a1 mutations cause severe myopathy. Matrix Biol. *31*, 29–37. https://doi.org/10.1016/j.matbio.2011.09.004.
25. Kiss, M., Kiss, A.A., Radics, M., Popovics, N., Hermesz, E., Csiszár, K., and Mink, M. (2016). Drosophila type IV collagen mutation associates with immune system activation and intestinal dysfunction. Matrix Biol. *49*, 120–131. https://doi.org/10.1016/j.matbio.2015.09.002.
26. Duncan, K.G., Fessler, L.I., Bächinger, H.P., and Fessler, J.H. (1983). Procollagen IV. Association to tetramers. J. Biol. Chem. *258*, 5869–5877.
27. Siebold, B., Qian, R.A., Glanville, R.W., Hofmann, H., Deutzmann, R., and Kühn, K. (1987). Construction of a model for the aggregation and cross-linking region (7S domain) of type IV collagen based upon an evaluation of the primary structure of the alpha 1 and alpha 2 chains in this region. Eur. J. Biochem. *168*, 569–575. https://doi.org/10.1111/j.1432-1033.1987.tb13455.x.
28. Horne-Badovinac, S. (2020). Mobilizing the matrix for organ morphogenesis. Dev. Cell *54*, 1–2. https://doi.org/10.1016/j.devcel.2020.06.014.
29. Keeley, D.P., Hastie, E., Jayadev, R., Kelley, L.C., Chi, Q., Payne, S.G., Jeger, J.L., Hoffman, B.D., and Sherwood, D.R. (2020). Comprehensive endogenous tagging of basement membrane components reveals dynamic movement within the matrix scaffolding. Dev. Cell *54*, 60–74.e7. https://doi.org/10.1016/j.devcel.2020.05.022.
30. Yurchenco, P.D., and Furthmayr, H. (1984). Self-assembly of basement membrane collagen. Biochemistry *23*, 1839–1850. https://doi.org/10.1021/bi00303a040.
31. Yurchenco, P.D., Tsilibary, E.C., Charonis, A.S., and Furthmayr, H. (1986). Models for the self-assembly of basement membrane. J. Histochem. Cytochem. *34*, 93–102. https://doi.org/10.1177/34.1.3510247.
32. Thompson, D.W. (1961). On Growth and Form (Cambridge University Press).
33. Lecuit, T., and Lenne, P.F. (2007). Cell surface mechanics and the control of cell shape, tissue patterns and morphogenesis. Nat. Rev. Mol. Cell Biol. *8*, 633–644. https://doi.org/10.1038/nrm2222.
34. Nerger, B.A., Brun, P.T., and Nelson, C.M. (2020). Marangoni flows drive the alignment of fibrillar cell-laden hydrogels. Sci. Adv. *6*, eaaz7748. https://doi.org/10.1126/sciadv.aaz7748.
35. Francis, L.F., McCormick, A.V., Vaessen, D.M., and Payne, J.A. (2002). Development and measurement of stress in polymer coatings. J. Mater. Sci. *37*, 4717–4731. https://doi.org/10.1023/A:1020886802632.
36. Szabó, A., Rupp, P.A., Rongish, B.J., Little, C.D., and Czirók, A. (2011). Extracellular matrix fluctuations during early embryogenesis. Phys. Biol. *8*, 045006. https://doi.org/10.1088/1478-3975/8/4/045006.
37. Luo, L., Liao, Y.J., Jan, L.Y., and Jan, Y.N. (1994). Distinct morphogenetic functions of similar small GTPases: drosophila Drac1 is involved in axonal outgrowth and myoblast fusion. Genes Dev. *8*, 1787–1802. https://doi.org/10.1101/gad.8.15.1787.
38. Zanet, J., Jayo, A., Plaza, S., Millard, T., Parsons, M., and Stramer, B. (2012). Fascin promotes filopodia formation independent of its role in actin bundling. J. Cell Biol. *197*, 477–486. https://doi.org/10.1083/jcb.201110135.
39. Page-McCaw, A., Serano, J., Santé, J.M., and Rubin, G.M. (2003). Drosophila matrix metalloproteinases are required for tissue remodeling, but not embryonic development. Dev. Cell *4*, 95–106.
40. Franke, J.D., Montague, R.A., and Kiehart, D.P. (2005). Nonmuscle myosin II generates forces that transmit tension and drive contraction in multiple tissues during dorsal closure. Curr. Biol. *15*, 2208–2221. https://doi.org/10.1016/j.cub.2005.11.064.
41. Van de Bor, V., Zimniak, G., Papone, L., Cerezo, D., Malbouyres, M., Juan, T., Ruggiero, F., and Noselli, S. (2015). Companion blood cells control ovarian stem cell niche microenvironment and homeostasis. Cell Rep. *13*, 546–560. https://doi.org/10.1016/j.celrep.2015.09.008.
42. Buszczak, M., Paterno, S., Lighthouse, D., Bachman, J., Planck, J., Owen, S., Skora, A.D., Nystul, T.G., Ohlstein, B., Allen, A., et al. (2007). The carnegie protein trap library: a versatile tool for Drosophila developmental studies. Genetics *175*, 1505–1531. https://doi.org/10.1534/genetics.106.065961.
43. Kohsaka, H., Takasu, E., and Nose, A. (2007). In vivo induction of postsynaptic molecular assembly by the cell adhesion molecule Fasciclin2. J. Cell Biol. *179*, 1289–1300. https://doi.org/10.1083/jcb.200705154.
44. Gyoergy, A., Roblek, M., Ratheesh, A., Valoskova, K., Belyaeva, V., Wachner, S., Matsubayashi, Y., Sánchez-Sánchez, B.J., Stramer, B., and Siekhaus, D.E. (2018). Tools allowing independent visualization and

CellPress
OPEN ACCESS

genetic manipulation of Drosophila melanogaster macrophages and surrounding tissues. G3 (Bethesda) *8*, 845–857. https://doi.org/10.1534/g3.117.300452.

45. Morin, X., Daneman, R., Zavortink, M., and Chia, W. (2001). A protein trap strategy to detect GFP-tagged proteins expressed from their endogenous loci in Drosophila. Proc. Natl. Acad. Sci. USA *98*, 15050–15055. https://doi.org/10.1073/pnas.261408198.

46. Voigt, A., Pflanz, R., Schäfer, U., and Jäckle, H. (2002). Perlecan participates in proliferation activation of quiescent Drosophila neuroblasts. Dev. Dyn. *224*, 403–412. https://doi.org/10.1002/dvdy.10120.

47. Rehorn, K.P., Thelen, H., Michelson, A.M., and Reuter, R. (1996). A molecular aspect of hematopoiesis and endoderm development common to vertebrates and Drosophila. Development *122*, 4023–4031.

48. Sepp, K.J., and Auld, V.J. (1999). Conversion of lacZ enhancer trap lines to GAL4 lines using targeted transposition in Drosophila melanogaster. Genetics *151*, 1093–1101. https://doi.org/10.1093/genetics/151.3.1093.

49. Bindels, D.S., Haarbosch, L., van Weeren, L., Postma, M., Wiese, K.E., Mastop, M., Aumonier, S., Gotthard, G., Royant, A., Hink, M.A., et al. (2017). mScarlet: a bright monomeric red fluorescent protein for cellular imaging. Nat. Methods *14*, 53–56. https://doi.org/10.1038/nmeth.4074.

50. Davis, J.R., Huang, C.Y., Zanet, J., Harrison, S., Rosten, E., Cox, S., Soong, D.Y., Dunn, G.A., and Stramer, B.M. (2012). Emergence of embryonic pattern through contact inhibition of locomotion. Development *139*, 4555–4560.

51. Lee, H.K., Wright, A.P., and Zinn, K. (2009). Live dissection of Drosophila embryos: streamlined methods for screening mutant collections by antibody staining. J. Vis. Exp. *34*, 1647.

52. Sánchez-Sánchez, B.J., Urbano, J.M., Comber, K., Dragu, A., Wood, W., Stramer, B., and Martín-Bermudo, M.D. (2017). Drosophila embryonic hemocytes produce laminins to strengthen migratory response. Cell Rep. *21*, 1461–1470. https://doi.org/10.1016/j.celrep.2017.10.047.

53. Chen, B.C., Legant, W.R., Wang, K., Shao, L., Milkie, D.E., Davidson, M.W., Janetopoulos, C., Wu, X.S., Hammer, J.A., 3rd, Liu, Z., et al. (2014). Lattice light-sheet microscopy: imaging molecules to embryos at high spatiotemporal resolution. Science *346*, 1257998. https://doi.org/10.1126/science.1257998.

54. Liu, T.L., Upadhyayula, S., Milkie, D.E., Singh, V., Wang, K., Swinburne, I.A., Mosaliganti, K.R., Collins, Z.M., Hiscock, T.W., Shea, J., et al. (2018). Observing the cell in its native state: imaging subcellular dynamics in multicellular organisms. Science *360*, eaaq1392. https://doi.org/10.1126/science.aaq1392.

55. Hirvonen, L.M., Marsh, R.J., Jones, G.E., and Cox, S. (2020). Combined AFM and super-resolution localisation microscopy: investigating the structure and dynamics of podosomes. Eur. J. Cell Biol. *99*, 151106. https://doi.org/10.1016/j.ejcb.2020.151106.

56. Preibisch, S., Saalfeld, S., and Tomancak, P. (2009). Globally optimal stitching of tiled 3D microscopic image acquisitions. Bioinformatics *25*, 1463–1465. https://doi.org/10.1093/bioinformatics/btp184.

57. Schindelin, J., Arganda-Carreras, I., Frise, E., Kaynig, V., Longair, M., Pietzsch, T., Preibisch, S., Rueden, C., Saalfeld, S., Schmid, B., et al. (2012). Fiji: an open-source platform for biological-image analysis. Nat. Methods *9*, 676–682. https://doi.org/10.1038/nmeth.2019.

58. Davis, J.R., Luchici, A., Mosis, F., Thackery, J., Salazar, J.A., Mao, Y., Dunn, G.A., Betz, T., Miodownik, M., and Stramer, B.M. (2015). Intercellular forces orchestrate contact inhibition of locomotion. Cell *161*, 361–373. https://doi.org/10.1016/j.cell.2015.02.015.

59. Yolland, L., Burki, M., Marcotti, S., Luchici, A., Kenny, F.N., Davis, J.R., Serna-Morales, E., Müller, J., Sixt, M., Davidson, A., et al. (2019). Persistent and polarized global actin flow is essential for directionality during cell migration. Nat. Cell Biol. *21*, 1370–1381. https://doi.org/10.1038/s41556-019-0411-5.

60. Marcotti, S., Reilly, G.C., and Lacroix, D. (2019). Effect of cell sample size in atomic force microscopy nanoindentation. J. Mech. Behav. Biomed. Mater. *94*, 259–266. https://doi.org/10.1016/j.jmbbm.2019.03.018.

STAR★METHODS

KEY RESOURCES TABLE

REAGENT or RESOURCE	SOURCE	IDENTIFIER
Experimental models: Organisms/strains		
D. melanogaster: w1118	Bloomington Drosophila Stock Center	RRID: BDSC_3605
D. melanogaster: repo-Gal4	Bloomington Drosophila Stock Center	RRID: BDSC_7415
D. melanogaster: elav-Gal4	see Luo et al.[37]	N/A
D. melanogaster: Sn-Gal4	see Zanet et al.[38]	N/A
D. melanogaster: PS7-Gal4	see Bowman et al.[23]	N/A
D. melanogaster: UAS-LifeActGFP	see Zamet et al.[38]	N/A
D. melanogaster: UAS-RedStinger	Bloomington Drosophila Stock Center	RRID: BDSC_8547
D. melanogaster: UAS-MMP2	see Page-McCaw et al.[39]	N/A
D. melanogaster: UAS-RacN17	Bloomington Drosophila Stock Center	RRID: BDSC_6292
D. melanogaster: UAS- ZipDN-GFP	see Franke et al.[40]	N/A
D. melanogaster: UAS-Zip RNAi	Vienna *Drosophila* Resource Center	RRID: Flybase_FBst0470845
D. melanogaster: UAS-Col4α2-GFP	see Van de Bor et al.[41]	N/A
D. melanogaster: UAS-LifeAct-mScarlet	This paper	N/A
D. melanogaster: UAS-Col4α1^{wt}-mScarlet	This paper	N/A
D. melanogaster: UAS-Col4α1^{D7S}-mScarlet	This paper	N/A
D. melanogaster: UAS-Col4α1^{G552D}-mScarlet	This paper	N/A
D. melanogaster: fax::GFP	see Buszczak et al.[42]	N/A
D. melanogaster: repo-Cherry	Gift from C. Gabernard	N/A
D. melanogaster: elav-mYFP	see Kohsaka et al.[43]	N/A
D. melanogaster: srpHemo-H2A::3xmCherry	see Gyoergy et al.[44]	N/A
D. melanogaster: CollVα2 (Vkg)-GFP	see Morin et al.[45]	N/A
D. melanogaster: Df(2L)LanB1 (ΔLaminin)	see Urbano et al.[14]	N/A
D. melanogaster: Df(2L)BSC172 (ΔCol4)	Bloomington Drosophila Stock Center	RRID: BDSC_9605
D. melanogaster: Cg25C^{k13420}	Kyoto Stock Center	RRID: DGGR_102873
D. melanogaster: trol^{null} (ΔPerlecan)	see Voigt et al.[46]	N/A
D. melanogaster: srp^{AS}	see Rehorn et al.[47]	N/A
D. melanogaster: Col4α1^{G552D}	see Kelemen-Valkony et al.[24]	N/A
D. melanogaster: Mhc^{1}	Gift from F. Schnorrer	N/A
Oligonucleotides		
Primer: 5' GGGAATTGGCCGGCCA ATTAATTAAATGGGTGTCGCAGATT TGATCAAG 3'	This paper	N/A
Primer: 5' CGCAGACCTAGGAAAGCTA GCTTACTTGTACAGCTCGTCCATGCC 3'	This paper	N/A
Primer: 5' CCCCGTAATGCAGAAGAAGA 3'	This paper	N/A
Primer: 5' GACACTGGACTCGATGGACA 3'	This paper	N/A
Primer: 5' GACACTGGACTCGATGGACA 3'	This paper	N/A
Primer: 5' GCTGGTATTCCCGGAGTTTC 3'	This paper	N/A
Recombinant DNA		
Plasmid: pUASt-5C	General Fly Transformation Vectors	RRID: DGRC_1261
Plasmid: pUASt-Col4α1^{wt}-mScarlet	see Matsubayashi et al.[8]	N/A

(Continued on next page)

Continued

REAGENT or RESOURCE	SOURCE	IDENTIFIER
Plasmid: pUASt-attB	General Fly Transformation Vectors	RRID: DGRC_1419
Plasmid: pUASt-LifeAct-mScarlet	This paper	N/A
Plasmid: pUASt-attB-Col4α1^{wt}-mScarlet	This paper	N/A
Plasmid: pUASt-attB-Col4α1^{D7s}-mScarlet	This paper	N/A
Plasmid: pUASt-attB-Col4α1^{G552D}-mScarlet	This paper	N/A
**Software and algorithms**		
LAS AF	Leica	http://leica-microsystems.com/home/
Zen	Carl Zeiss	https://www.zeiss.com/microscopy/int/products/microscope-software/zen-lite.html
Zen Black	Carl Zeiss	https://www.zeiss.com/microscopy/int/products/microscope-software/zen.html#downloads
ImageJ/Fiji	Fiji	http://fiji.sc/
Imaris	Bitplane	https://imaris.oxinst.com
Abaqus	Dassaults Systèmes	https://www.3ds.com/products-services/simulia/products/abaqus/
MATLAB	MathWorks	https://uk.mathworks.com/products/matlab.html
Photoshop	Adobe	https://www.adobe.com/uk/products/photoshop.html
Illustrator	Adobe	https://www.adobe.com/uk/products/illustrator.html
Prism	GraphPad	https://www.graphpad.com
Excel	Microsoft	https://www.microsoft.com/en-gb/microsoft-365/excel
**Other**		
In-Fusion Cloning	Takara Bio USA	638920
Drosophila injection service	BestGene	https://thebestgene.com/
10S Voltalef oil	VWR	24627.188
Glass-bottom dishes	WPI	https://www.wpi-europe.com/products/cell-and-tissue/fluorodish-cell-culture/fd35-100.aspx
M205 fluorescent dissection microscope	Leica	http://leica-microsystems.com/home/
PLANAPO 2.0x objective for M205	Leica	10450030
LSM 880 confocal microscope	Carl Zeiss	https://www.zeiss.co.uk/microscopy/dynamic-content/news/2014/news-lsm-880.html
63x NA 1.4 Plan-Apochromat oil objective for LSM 880	Carl Zeiss	https://zeiss.com/corporate/int/home.html
Cooling/Heating Plate TP-CHS-C	TOKAI HIT	https://www.tokaihit.com/products/TP-CHS-C/

RESOURCE AVAILABILITY

Lead contact

Further information and requests for resources and reagents should be directed to and will be fulfilled by the lead contact, Brian Stramer (brian.m.stramer@kcl.ac.uk).

Materials availability

Drosophila lines generated in this study are available from the lead contact upon reasonable request.

Data and code availability

Microscopy data reported in this paper will be shared by the lead contact upon reasonable request. The code is deposited in publicly available repositories as specified in the relevant sections below. Any additional information required to reanalyze the data reported in this paper is available from the lead contact upon request.

EXPERIMENTAL MODEL AND SUBJECT DETAILS

Fly stocks and preparations

Repo-Gal4[48] and elav-Gal4[37] were used to target transgene expression in the glial cells and neurons forming the VNC. Sn-Gal4[38] was used to express transgenes specifically in hemocytes. The parasegmental driver PS7-Gal4[23] was used to express transgenes in a narrow stripe along the VNC. The following UAS lines were used: UAS-LifeActGFP,[38] UAS-RedStinger, UAS-MMP2,[39] UAS-Rac DN,[37] UAS-Myosin II DN,[40] UAS-Col4a2-GFP,[41] UAS-LifeAct-mScarlet, UAS-Col4a1^{wt}-mScarlet, UAS- Col4a1^{D7S}-mScarlet, and UAS- Col4a1^{G552D}-mScarlet (see following sections). Fax::GFP,[42] repo-Cherry (gift from Clemens Cabernard, University of Basel), and elav-mYFP[43] were used to label the VNC independently of Gal4. SrpHemo-H2A::3xmCherry[44] was used to label the nuclei of hemocytes independently of Gal4. The homozygous viable Col4a2-GFP[45] protein trap was used to visualize Col4. The following mutant alleles and deficiencies were used: Df(2L)LanB1 (referred to as DLaminin, removing lanB1),[14] Df(2L) BSC172 (referred to as DCol4, removing a chromosomal region including the two *Drosophila* Col IV genes vkg and Cg25C), Cg25C^{k13420} (Col4a1 mutant), trol^{null} (referred to as DPerlecan),[46] srp^{AS} (lacking hemocytes),[47] Col4a1^{G552D} (referred as G552D and also known as *DTS-L2*, containing an aspartic acid substitution in Col4a1 at G552),[24] *Mhc*[1] (gift from Frank Schnorrer and characterized for its use in imaging the *Drosophila* embryo in Matsubayashi et al.[8]). Unless used for temperature switch experiments, the flies were left to lay eggs on grape juice agar plates overnight at 25°C. Embryos were dechorionated in bleach. The appropriate genotype of the embryos was identified based on the presence of fluorescent probes and/or the absence of balancer chromosome expressing fluorescent markers. The genotypes of the embryos used in each experiment are listed in the supplemental information (Table S1).

METHOD DETAILS

Construction of UAS-LifeAct-mScarlet, UAS- Col4a1^{wt}-mScarlet, UAS- Col4a1^{D7s}-mScarlet and UAS-Col4a1^{G552D}-mScarlet

pUASt-LifeAct-mScarlet was generated by inserting an 802 bp fragment (synthesized by eurofins Genomics) containing LifeAct followed by mScarlet-I sequences,[49] and an extra 15 bp at the 3' and 5' ends allowing their insertion into the linearized NheI-PacI pUASt-5C plasmid using In-Fusion cloning strategy (Takara Bio USA, Inc.).

The construct was sequenced (eurofins Genomics) using the following sequencing primers:

5' GGGAATTGGCCGGCCAATTAATTAAATGGGTGTCGCAGATTTGATCAAG 3'
5′ CGCAGACCTAGGAAAGCTAGCTTACTTGTACAGCTCGTCCATGCC 3′

pUASt-attB-Col4a1^{wt}-mScarlet was generated inserting a 15 kb fragment containing the Col4a1^{wt}-mScarlet sequence from pUASt-Col4a1^{wt}-mScarlet,[8] into the linearized PacI-AvrII pUASt-attB plasmid using ligation T4 strategy (New England Biolabs, Inc.).

pUASt-attB-Col4a1^{D7s}-mScarlet was generated by replacing a region of the 7s domain sequence of the pUASt-attB-Col4a1^{wt}-mScarlet with a truncated version (Figure S4). A 950 bp fragment containing the 7s region was excised from the plasmid using XhoI and AsiSI sites and substituted by a 588 bp fragment (synthesized by Bio Basic USA, Inc.) containing the 7s truncated region, into the linearised XhoI-AsiSI pUASt-attB-Col4a1^{wt}-mScarlet plasmid using ligation T4 strategy (New England Biolabs, Inc.).

The constructs were sequenced using the following sequencing primers:

5' CCCCGTAATGCAGAAGAAGA 3'
5' GACACTGGACTCGATGGACA 3'

pUASt-attB-Col4a1^{G552D}-mScarlet was generated by replacing the sequence for the 552 Glycine of Col4a1^{wt} sequence (GGC) of the pUASt-attB-Col4a1^{wt}-mScarlet for a new sequence which produces an Aspartic acid (GAC). A 2003 bp region containing the wild-type version of 552 Glycine was excised from the plasmid using AsiSI and EcoNI sites and substituted by a 2003 bp fragment (synthesised by Bio Basic USA, Inc.) containing the sequence for 552 Aspartic, into the linearized AsiSI-EcoNI pUASt-attB-Col4a1^{wt}-mScarlet plasmid using ligation T4 strategy (New England Biolabs, Inc.).

The constructs were sequenced using the following sequencing primers:

5' GACACTGGACTCGATGGACA 3'
5' GCTGGTATTCCCGGAGTTTC 3'

The plasmids obtained were injected into flies (BDSC 9744) which contain an attP-9A insertion at 3R chromosome (89E11) by BestGene.

Sample preparation and mounting for imaging

For all the confocal imaging, dechorionated embryos were mounted in a drop of Voltalef oil (VWR) between a glass coverslip covered with heptane glue and a gas-permeable Lumox culture dish (Sarstedt) as described previously.[50] To image the embryos during stage 17 of embryogenesis on the confocal microscope, muscle myosin heavy chain (*Mhc*[1]) mutant embryos that do not twitch but have normal VNC condensation and expression of BM components were used as previously described.[8] For the widefield imaging, the embryos were mounted in the same way but without heptane glue. For lattice light-sheet imaging, the dechorionated embryos were mounted on a 25 mm glass coverslip covered with heptane glue; the embryos were then covered with a drop of PBS and transferred to the microscope chamber, where imaging was performed in PBS. Temperature controlled experiments were performed by mounting the gas-permeable Lumox culture dish on a TOKAI HIT TP-CHS-C heating/cooling plate.

For the AFM experiments, embryos were prepared as previously described.[9,51,52] After dechorionation with bleach, embryos were transferred to the heptane glue covered glass bottom of a 35 mm cell culture dish (FD35-100) which was then filled with 1x PBS. A cut was made along the dorsal midline of the embryos using an insect pin/needle, exposing the dorsal surface of the VNC.

Widefield and confocal microscopy

Widefield images were acquired every 2 min with an M205 fluorescent dissection microscope (Leica) equipped with a PLANAPO 2.0x objective. All the confocal imaging was performed using an LSM880 confocal microscope (Carl Zeiss) equipped with a 63x NA 1.4 Plan-Apochromat oil objective unless stated otherwise. Tilescans (8 x 2 overlap 10%) of 40 mm Z-stacks with a zoom of 1.2 were used for PIV dynamics of the full VNC, with lateral view and temporal resolution of 10 min. For live imaging of Col4 and glia in the tail of the VNC, 20 mm Z-stacks of Col4a2-GFP and repo>LifeAct-mScarlet were acquired with a 63X objective with 2x zoom, and a temporal resolution of 5 min/frame using Optimal Airyscan mode. For simultaneous imaging of Col4 in head vs. tail of the VNC, multipoint acquisition consisting of 20 mm Z-stacks of Col4a2-GFP in head and tail regions of the same embryo were acquired with a 63X objective with 4x zoom, and a temporal resolution of 30 sec/frame using Super Resolution Airyscan mode. To visualize Col4 on the surface of the full VNC with a ventral view, tilescans (10 x 2 overlap 0%) of 20 mm Z-stacks were acquired on Super Resolution Airyscan mode with a zoom of 4. For volume measurements of the full VNC, two tiled images (overlap 10%) of 100 mm Z-stacks with a 20x air objective (Plan-Apochromat air objective, NA 0.8) were acquired with a temporal resolution of 10 min. Images were stitched using the Zen Black software and exported to the Imaris software (Bitplane) for further analysis.

Lattice light-sheet microscopy and patterned photobleaching

Lattice light-sheet imaging[53,54] and photobleaching of the VNC was performed on the Multimodal Optical Scope with Adaptive Imaging Correction (MOSAIC) system developed at HHMI Janelia Research Campus (unpublished data). Experiments were performed at the HHMI Janelia Research Campus Advanced Imaging Center. For lattice light-sheet imaging, a Thorlabs 0.6 NA water dipping lens (TL20X-MPL) is used for excitation, and a Zeiss 1.0 NA water dipping objective (421452-9800) is used for detection. A square lattice pattern (Inner NA: 0.34; Outer NA: 0.4; Envelope: 3; Crop: 10) was used for generating the lattice light-sheet. Excitation was achieved via 488 nm and 560 nm laser lines, and emission was detected by two Hamamatsu Orca Flash 4.0 sCMOS cameras (dichroic: Semrock Di03-R561-t3-32x40; emissions filters: Camera 1 – Semrock 520/35 nm bandpass filter FF01-520/35-25, Semrock 561 nm notch filter NF03-561E-25; Camera 2 - Semrock 617/73 nm bandpass filter FF02-617/73-25). A system correction for the excitation and detection paths was performed as previously described.[54] To image the entire depth of the VNC, a 2x2x2 tiled acquisition was performed using the "Z galvo & DO XZ stage" motion, wherein the sample stage is moved directly in line with the optical axis of the detection objective (Single tile size: 512 x 1500 x 201 voxels; voxel size: 108 x 108 x 250 nm; 50 ms per frame, total time between volumes: 5 minutes). To image the surface of the VNC, a 1x2x1 tiled acquisition was performed using the "X Stage" motion, wherein the sample stage is scanned laterally at an angle of 32.45º relative to the optical axis of the detection objective (Single tile size: 512 x 1500 x 251 voxels; voxel size after deskewing: 108 x 108 x 268 nm; 50 ms per frame, total time between volumes: 2 minutes).

The 1x2x1 tiling was used for the photobleaching experiments. Prior to photobleaching, a single, tiled lattice light-sheet volume was collected. The MOSAIC configuration was then automatically switched to a custom point-scanning configuration in which a single Gaussian beam was passed through the lattice light-sheet excitation objective. This beam was dithered in both directions orthogonal to its propagation axis while simultaneously moving the sample stage in the direction of the optical axis of the excitation objective, resulting in a photobleached stripe of approximately 20 μm width perpendicular to the VNC midline. A total of 5 stripes were photobleached along the VNC midline, spaced at approximately 50 μm from each other. After the 5 stripes were photobleached, the MOSAIC configuration was automatically switched back to the lattice light-sheet imaging configuration for subsequent tiled, volumetric imaging as described above.

Atomic force microscopy (AFM)

Spherical tipped cantilevers were produced by gluing a 10 μm silica bead at the extremity of qp-CONT probes (Nanosensors). The cantilever spring constant was calibrated in liquid by thermal noise method (0.2 N/m). Measurements were performed using a custom-built system,[55] based on a standard inverted microscope (Axio Observer.Z1, Zeiss) and equipped with an atomic force microscope (NanoWizard 3, JPK Instruments).

Force spectroscopy measurements using a 3 nN set point were performed on the VNC towards the head side of the tissue.

QUANTIFICATION AND STATISTICAL ANALYSIS

Lattice light-sheet imaging pre-processing

For lattice light-sheet movies acquired with x stage motion, deskewing is required to reconstruct the stacks in the z direction by correcting the shearing of the data during acquisition.[53] The pipeline employed to this aim can be found here: https://github.com/aicjanelia/LLSM.

Tilescan stitching used the stitching-spark pipeline developed by the Saalfeld group (https://github.com/saalfeldlab/stitching-spark). The first time point of each dataset was stitched using a Fourier Shift Transform to find the best possible overlap between tiles.[56] The stitched tile positions from this initial time point were then used for all other time points in the series. The entire time series was exported as blended images for further visualization and analysis.

To run particle image velocimetry (PIV) analysis on lattice light-sheet movies encompassing the whole VNC tissue from the ventral view, 3D segmentation of the tissue was required to remove unwanted signal from imaging planes outside the VNC. To this aim, a segmentation pipeline was implemented, where segmentation is performed manually in MATLAB by drawing polygons around the tissue on sparse z-planes (1 every 20 z-slices) for each time point. By using the segmented planes, a triangulated volume is created and re-sliced to obtain segmentation of intermediate slices.

VNC condensation and Col4 levels quantification by fluorescent dissection microscope

For the quantification of VNC condensation, the last position of the tail of the VNC was manually tracked on every frame (2 min time resolution) by using Fiji.[57] The x and y coordinates of each point were stored and compared to the initial position of the VNC tail to quantify displacement through time.

For the quantification of relative Col4a2-GFP-trap levels through time (2 min time resolution), the average raw fluorescence intensity of each embryo in every frame was measured with Fiji, the acquired data were smoothed by calculating a 10-frame moving average.

Volume and surface measurements

To visualise the VNC and measure its volume, the glial cells of the VNC were labelled with UAS-LifeAct-mScarlet under the expression of the glia driver repo-Gal4. To determine the 3 timepoints relevant to the condensation process we proceeded as follows: the beginning of the 1st phase of condensation was determined as the first frame in which displacement of the VNC tail was observed; the end of the 1st phase was determined as 3 hours from the beginning of the 1st phase; and the end of the 2nd phase as 9 hours from the end of the 1st phase. After the frames were selected, each peripheral nerve sprouting from the VNC was manually deleted, by using the "Surface" function in Imaris and by turning the voxels from the magenta channel into zero in the selected areas. Subsequently, the volume of the VNC was obtained by generating a surface on the magenta channel with a voxel of size 2. The length, width, and height of the VNC were quantified with the Imaris function "Measurement Points". For each embryo, the average height and width were measured at the central point of each neuromere of the VNC. We used the measured VNC dimensions to calculate the change in surface area, using an idealized shape resembling an elliptical cylinder.

Quantification of fluorescence intensity in the surface of the VNC

To quantify the Col4 gradient on the VNC surface from confocal images, we first removed the signal from the intra-cellular Col4a2-GFP inside hemocytes by discarding any signal higher than 70% of the total fluorescence intensity. We then calculated the average signal across the VNC from head to tail smoothed with a walking average over 200 μm.

Particle image velocimetry (PIV) analysis

PIV was performed with a custom MATLAB suite (https://github.com/stemarcotti/PIV) as in Davis et al.[58] and Yolland et al.[59] This methodology was applied on maximum intensity projected time series confocal and lattice light-sheet imaging of the VNC and was used to track the motion on the VNC surface over time. Briefly, the algorithm cross-correlates a small region of interest in a frame of reference (source area) to a larger portion of the subsequent time frame (search area), to find the best match. This operation is performed iteratively on all portions of the image at each time frame to allow for feature tracking in the entire field of view. The parameters were optimized as follows: source size 9 μm, search size 16 μm, grid size 5 μm, correlation threshold 0.5, when the entire VNC was imaged with confocal microscopy; source size 2 μm, search size 4 μm, grid size 1 μm, correlation threshold 0.3, when only a portion of the VNC was imaged with high spatiotemporal resolution with confocal microscopy; source size 5 μm, search size 10 μm, grid size 3 μm, correlation threshold 0.5, when the entire VNC was imaged on the MOSAIC system.

The obtained displacement vector field was then interpolated both spatially and temporally. The interpolation kernel parameters were set as follows: spatial kernel size 50 μm (sigma 10 μm), temporal kernel size 90 min (sigma 40 min), when the entire VNC was imaged with confocal microscopy and the MOSAIC system; spatial kernel size 10 μm (sigma 2 μm), temporal kernel size 5 frames (sigma 2 frames), when only a portion of the VNC was imaged with high spatiotemporal resolution with confocal microscopy. All colourmaps presented were generated to visualize the magnitude of feature velocity, and vectors from the interpolated field were overlaid to highlight flow direction. An average of the velocity magnitude or x component across the length of the VNC was calculated for each frame to plot kymographs.

PIV local alignment correlation

To evaluate the coherence of motion for collagen and glia, 256 random locations were selected on the non-interpolated PIV displacement vector field for each time frame. The displacement vector at each of these locations was considered as a reference, and its orientation was compared to all the neighbouring vectors over an 8 μm radius. This was done by calculating the norm of the cosine of the angle theta between the reference vector and each of the neighbouring vectors, and by subsequently obtaining an average across all these comparisons for each location. The computed cosine values were smoothed with a walking average over 5 frames (25 min). Values close to 1 indicate similar orientation between the reference and its neighbours, representing high motion coherence.

A similar approach was taken to measure the coherence of motion for collagen in the tail-to-head direction for two distinct locations at the head and tail of the VNC. For each frame, the direction of 11x11 vectors over a 3 μm-spaced grid of the interpolated PIV field was compared to the reference direction, i.e., tail-to-head direction. The cosine of the angle theta between each vector and the reference vector was calculated as a measure of coherence of motion in the tail-to-head direction, and an average calculated for each frame. Values close to 1 indicate motion in the tail-to-head direction.

Cell tracking

Confocal time-lapse movies of the glial cells forming the VNC containing labelled nuclei were tracked with the Imaris function "Spots". The tracking was divided in two areas, anterior (head) and posterior (tail), which were defined as half of the VNC at the end of the initial 3 hours of VNC condensation. Two 3-hour periods were chosen in the first and second phase, respectively (0-3 hours and 7-10 hours from the start of VNC condensation).

The obtained tracks were analysed with custom software in MATLAB to obtain information on their directionality. The start and end (x,y) coordinates of each track within these time periods which was longer than 10 frames (100 min) were extracted. A vector was drawn between each start and end location and its angle computed to display the polar histograms (angles corresponding to zero were set on the horizontal axis from head to tail). 81 and 130 tracks were analysed for the first phase for head and tail, respectively; 219 and 206 tracks were analysed for the second phase for head and tail, respectively. The mean average velocity of all the tracks with a minimum length of 10 frames (100 min) in both areas was obtained from the Imaris function "Spots".

Analysis of photobleaching

To analyse the anisotropic motion of the VNC when photobleaching stripes across the tissue, a line was drawn in Fiji with thickness 100 px along the midline of the VNC (perpendicular to the stripes). Intensity of the signal in the region highlighted by the linescan was obtained at two different timepoints (immediately and 30 min after photobleaching), normalised and averaged across a moving window of 150 datapoints. The five local minima corresponding to centre of the stripe were automatically located in MATLAB and used to visualized the anisotropic motion from tail to head of the tissue.

Eccentricity of VNC cross-sections

MOSAIC movies of the whole VNC with glial cells labelled were used to evaluate the eccentricity of the tissue cross-section. 3D reconstructions were obtained in Imaris and regions corresponding to 3 separate neuromers were selected for two time-points representing stage 15 and 17 of embryo development. Manual segmentation of the tissue was performed in Fiji, and the outlines were centred and aligned to the horizontal axis in MATLAB for visualisation purposes. The eccentricity was calculated in MATLAB as the ratio of the distance between the foci of the ellipse fitting the VNC outline and its major axis length. Values for eccentricity can vary between 0 (circle) and 1 (line).

AFM analysis

Analysis of the force spectroscopy curves to obtain the effective modulus was carried out in MATLAB by using custom-made algorithms as described in Marcotti et al.,[60] considering a target indentation depth for the Hertz model fitting of 230 nm.

Statistics and reproducibility

Statistical tests employed are listed in the caption of relevant figure panels. Significance was indicated as follows: '****' for p-values lower than 0.0001, '***' lower than 0.001, '**' lower than 0.01, '*' lower than 0.05, 'ns' otherwise.

Developmental Cell, Volume *58*

Supplemental information

Extracellular matrix assembly stress initiates *Drosophila* central nervous system morphogenesis

Eduardo Serna-Morales, Besaiz J. Sánchez-Sánchez, Stefania Marcotti, Angus Nichols, Anushka Bhargava, Anca Dragu, Liisa M. Hirvonen, María-del-Carmen Díaz-de-la-Loza, Matyas Mink, Susan Cox, Emily Rayfield, Rachel M. Lee, Chad M. Hobson, Teng-Leong Chew, and Brian M. Stramer

Supplementary Figures

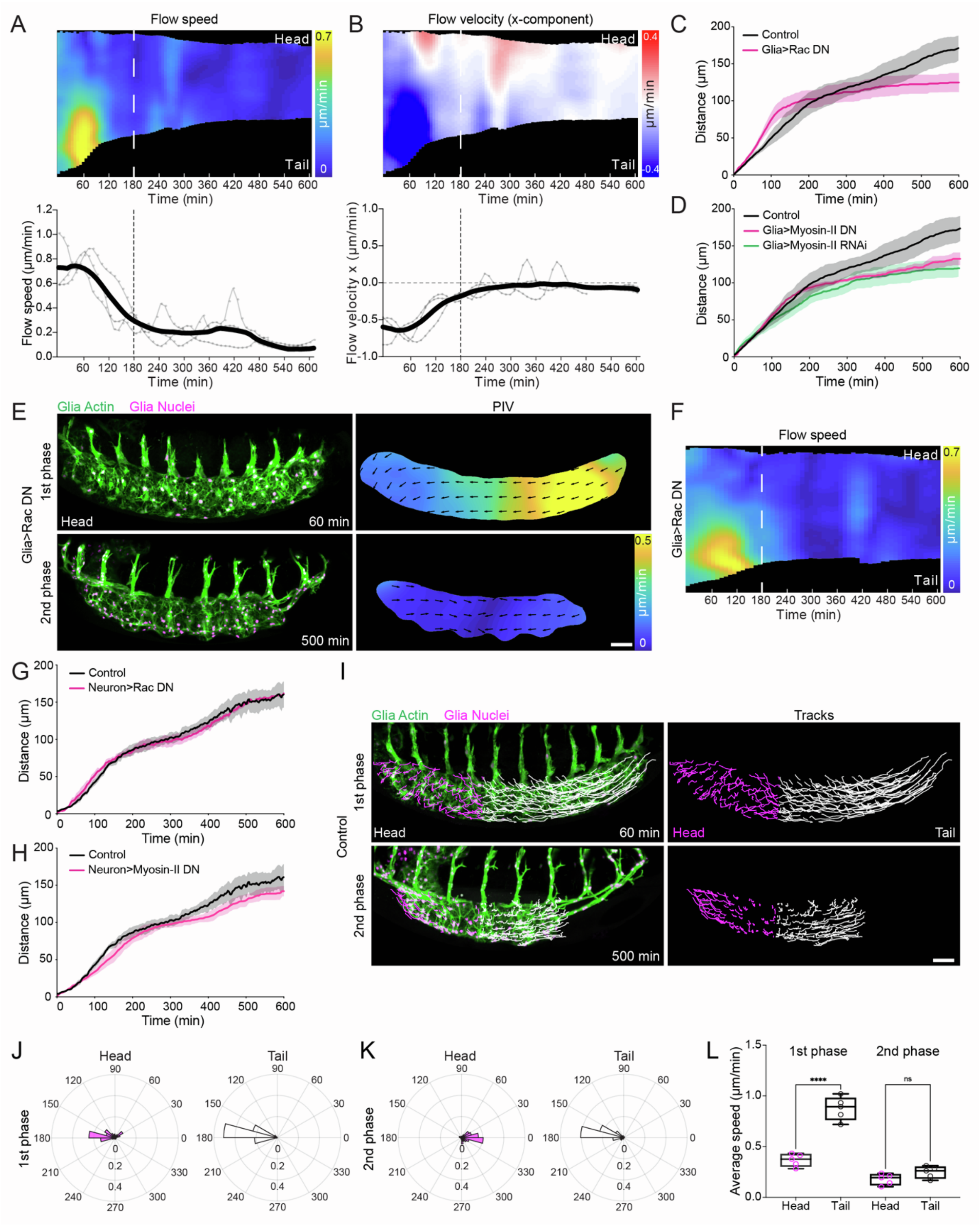

Fig. S1. *Drosophila* VNC condensation consists of two distinct temporal stages and initial anisotropic changes in tissue morphology are independent of VNC cellular activity, related to Figure 1. (A) (top panel) Kymograph of the average speed of VNC condensation from head to tail of the tissue from PIV analysis as shown in Figure 1A. Note the asymmetric increase in speed in the tail of the tissue during the first 3 hours of condensation. (bottom panel) Timecourse of the average speed of VNC condensation as measured above highlighting a transition from fast to slow phases around 3 hours. n = 3 embryos. **(B)** (top panel) Kymograph of the average x-component of the velocity along the length of the tissue from head to tail during VNC condensation. Note the predominant negative velocity in the first 3 hours of condensation, which highlights specific motion of the tail of the tissue towards the head. (bottom panel) Timecourse of the average velocity of the x-component as measured above highlighting that during the first 3 hours condensation is predominantly anisotropic from tail to head (*i.e.*, the x-component is negative). After 3 hours the average x-component approaches zero suggesting that this longer phase of condensation is symmetric (*i.e.*, equivalent from the head and tail of the tissue). n = 3 embryos. **(C,D)** Quantification of VNC condensation by tracking the tail of the tissue as in Figure 1D,E after expressing dominant negative (DN) Rac (C), or DN and RNAi Myosin-II (D) in glia reveals that the rate is only affected during the 2nd phase of condensation. Control data is the same in both panels (n = 4 embryos for control, n = 3 embryos for Glia>Rac DN and Glia>Myosin-II DN). **(E)** Live imaging of VNC morphogenesis as in Figure 1A while expressing Rac DN in glia reveals the presence of an anisotropic 1st phase of condensation. Scale bar = 30 µm. **(F)** Kymograph of the average speed of VNC condensation from head to tail of the tissue from PIV analysis in panel (E). Note the asymmetric increase in speed in the tail of the tissue during the first 3 hours of condensation despite the expression of Rac DN. **(G,H)** Quantification of VNC condensation by tracking the tail of the tissue as in Figure 1D,E after expressing dominant negative (DN) Rac (G) or DN Myosin-II (H) in neurons reveals little if any effect on VNC condensation (n = 3 embryos for each sample). **(I)** Glial cell motion in the head (magenta) vs tail (white) of the tissue tracked during the 1st and 2nd phases of condensation. Scale bar = 30 µm. **(J,K)** Quantification of the direction of tracks in panel (I) reveals that the cells predominantly move in a tail to head direction (towards 180°) during the first phase (J). In contrast, during the isotropic 2nd phase of condensation the cell tracks are predominantly moving symmetrically towards the centre of the VNC (K). n = 3 embryos. **(L)** Quantification of average cell speed during the 1st and 2nd phases of condensation reveals that the motion of cells is fastest within the tail of the tissue during the 1st phase of condensation, while showing no local difference in speed during the 2nd phase. Two-way ANOVA and Tukey's multiple comparisons test. n = 5 embryos. Each dot represents one embryo. Boxplots show medians, 25th and 75th percentiles as box limits, 10th and 90th percentiles as whiskers. ****p<0.0001, ns p = 0.1196.

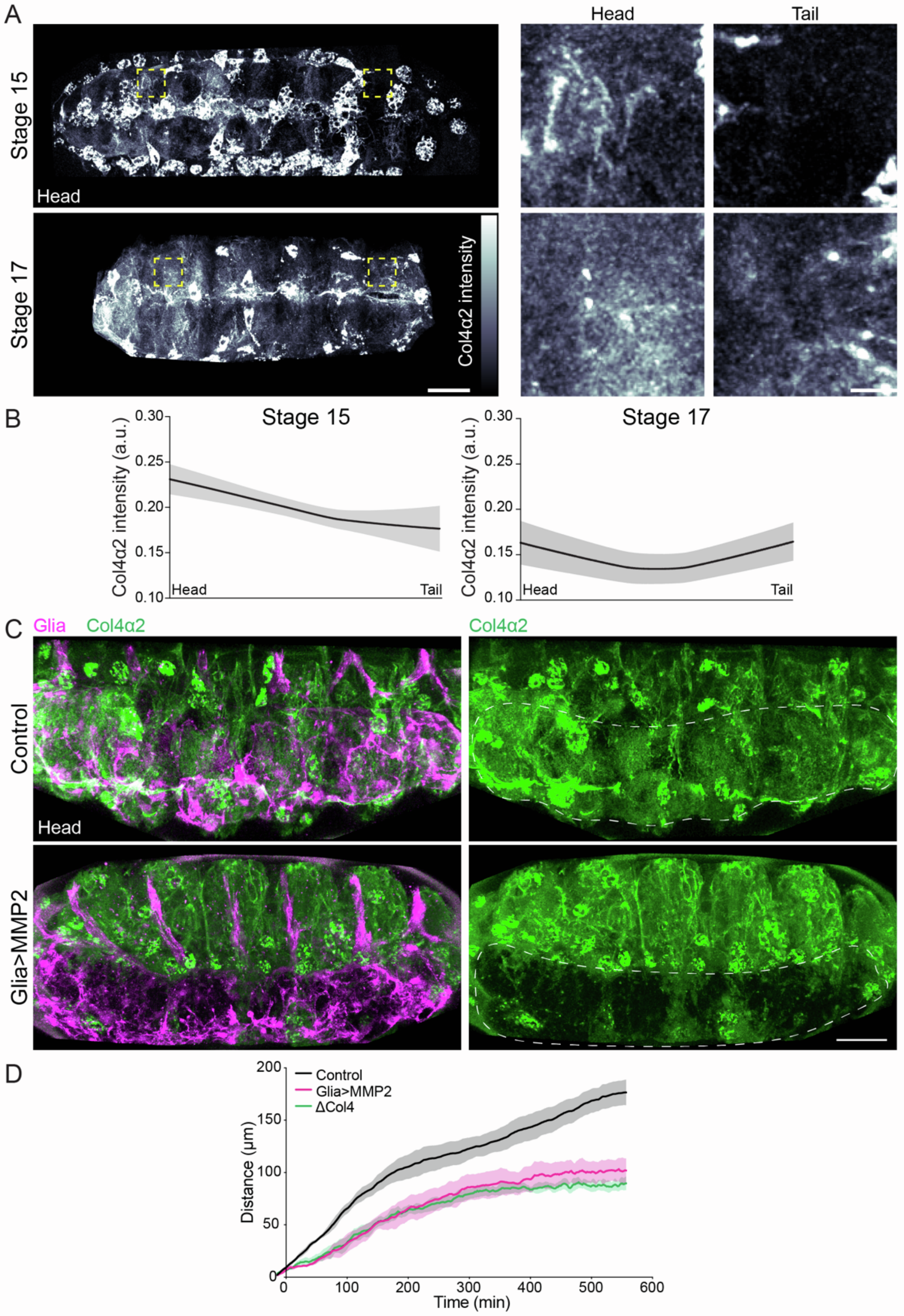

A
Head
Tail
Stage 15
Head
Stage 17
Col4α2 intensity
B
Stage 15
Stage 17
Col4α2 intensity (a.u.)
0.30
0.25
0.20
0.15
0.10
Head
Tail
C
Glia
Col4α2
Control
Head
Glia>MMP2
D
Control
Glia>MMP2
ΔCol4
Distance (μm)
200
150
100
50
0
Time (min)
0
100
200
300
400
500
600

Fig. S2. Col4 accumulates along the surface of the VNC and forms a transient gradient during the 1st phase of condensation, and local disruption of the Col4 network around the VNC phenocopies defects observed in Col4 mutants, related to Figures 1 and 2. **(A)** Live imaging of Col4 accumulation on the surface of the VNC during the 1st phase (stage 15) and 2nd phases (stage 17) of condensation. Right panels are high magnification views of the regions highlighted by the yellow squares. Scale bars = 30 µm (left panels) or 5 µm (right panels). **(B)** Quantification of Col4 intensity from head to tail of the VNC reveals a transient gradient during the 1st phase of condensation. n = 3 embryos for each stage. **(C)** Live imaging of Col4 and glia in control embryos and embryos driving MMP2 specifically in glia. Note the local disruption of Col4 accumulation surrounding the VNC (white outline). Scale bar = 30 µm. **(D)** Quantification of VNC condensation by tracking the tail of the tissue as in Figure 1D,E in *col4* mutants or after expressing MMP2 in glia reveals similar effects on the rate of condensation. Control and *col4* mutant data reused from Figure 2B (n = 4 embryos for control, n = 3 embryos for Glia>MMP2 and ΔCol4).

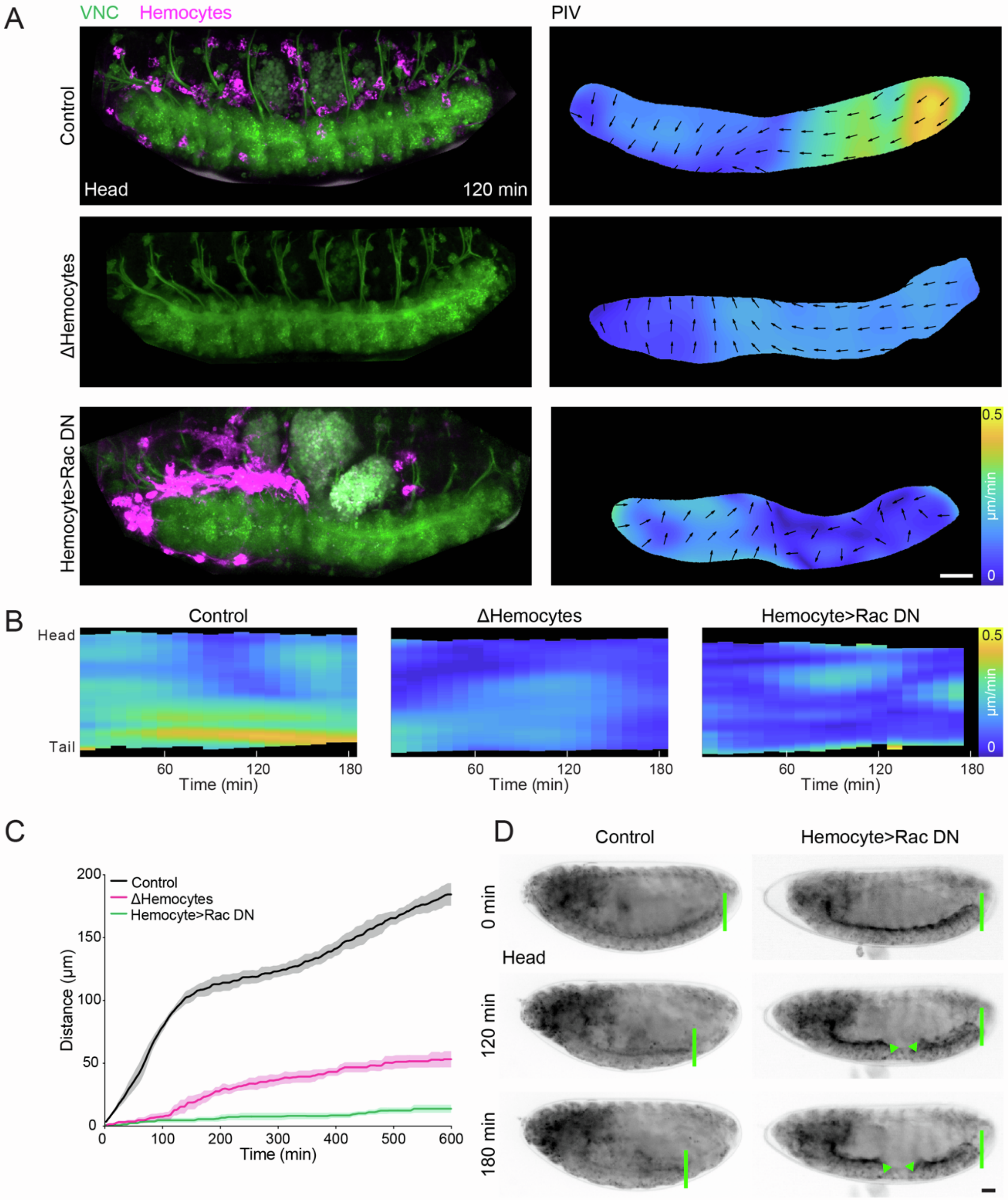
A
VNC Hemocytes
PIV
Control
Head
120 min
ΔHemocytes
Hemocyte>Rac DN
0.5
µm/min
0
B
Control
ΔHemocytes
Hemocyte>Rac DN
Head
Tail
60
120
180
Time (min)
C
Control
ΔHemocytes
Hemocyte>Rac DN
Distance (µm)
200
150
100
50
0
0
100
200
300
400
500
600
Time (min)
D
Control
Hemocyte>Rac DN
0 min
Head
120 min
180 min

Fig. S3. Inhibiting hemocyte migration severely affects VNC condensation, related to Figure 2. (A) Live imaging of VNC morphogenesis (left panels) and PIV (right panels) during the 1st phase of condensation as in Figure 1A in control, deletion of hemocytes, and expression of Rac DN specifically in hemocytes, which leads to their accumulation in the head of the embryo. Scale bar = 30 µm. **(B)** Kymograph of the average speed of VNC condensation from PIV analysis in (A) highlighting the absence of an anisotropic phase of condensation when perturbing hemocytes. **(C)** Quantification of VNC condensation by tracking the tail of the tissue as in Figure 1D,E in the genotypes highlighted in panel (A) reveals that loss of hemocytes or inhibiting their migration leads to severe defects in the rate of VNC condensation. n = 3 embryos for each sample. **(D)** Time-lapse series of VNC condensation (green lines) in controls and embryos expressing Rac DN specifically in hemocytes. Note that VNC condensation is completely inhibited as the VNC is severely deformed and appears to sever in the center of the embryo (arrowheads). Scale bar = 30 µm.

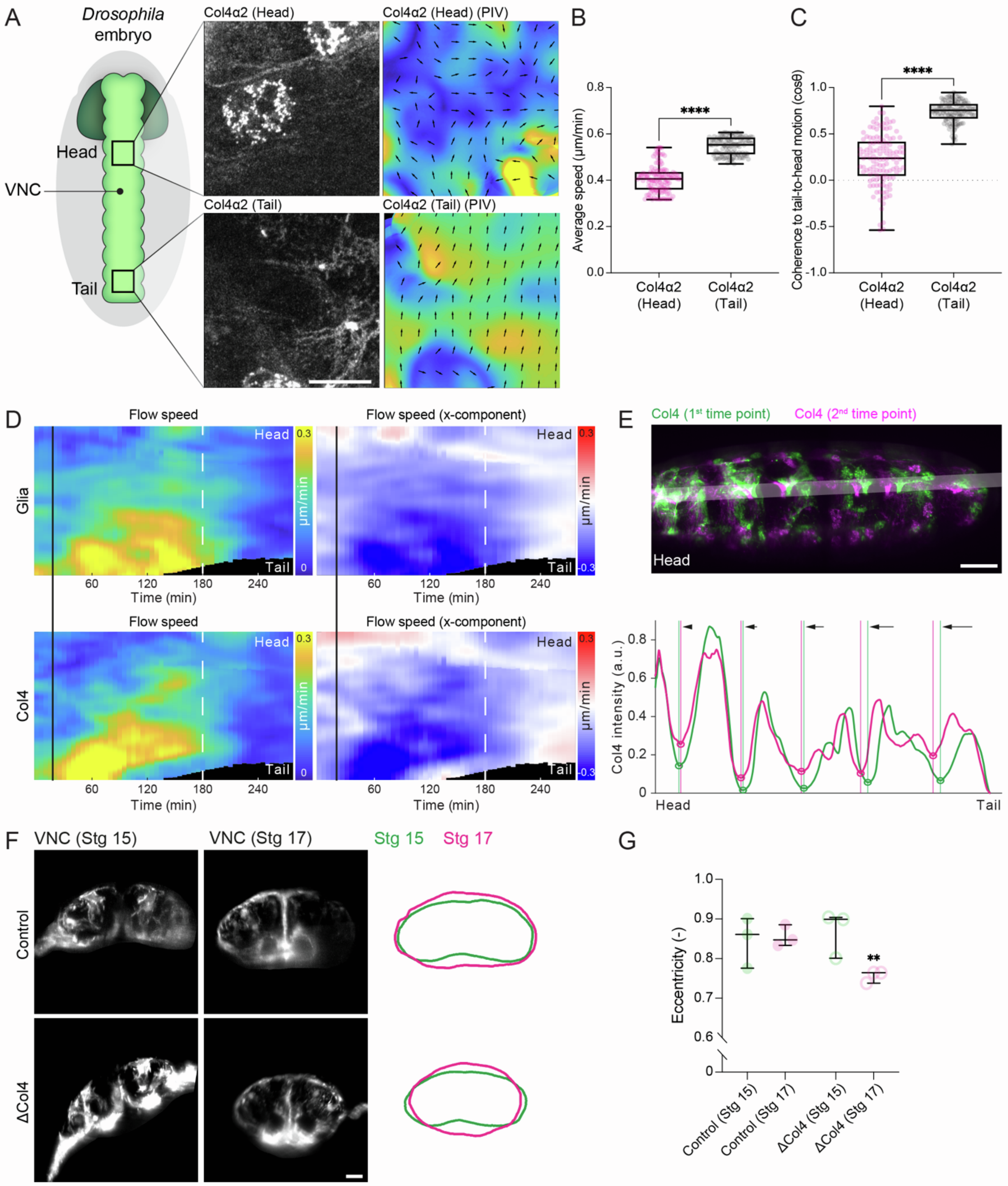
A
Drosophila embryo
Head
VNC
Tail
Col4α2 (Head)
Col4α2 (Head) (PIV)
Col4α2 (Tail)
Col4α2 (Tail) (PIV)
B
Average speed (μm/min)
Col4α2 (Head)
Col4α2 (Tail)
****
C
Coherence to tail-to-head motion (cosθ)
Col4α2 (Head)
Col4α2 (Tail)
****
D
Flow speed
Flow speed (x-component)
Glia
Col4
Head
Tail
μm/min
Time (min)
E
Col4 (1st time point)
Col4 (2nd time point)
Head
Col4 intensity (a.u.)
Head
Tail
F
VNC (Stg 15)
VNC (Stg 17)
Stg 15
Stg 17
Control
ΔCol4
G
Eccentricity (-)
Control (Stg 15)
Control (Stg 17)
ΔCol4 (Stg 15)
ΔCol4 (Stg 17)
**

Fig. S4. Coherent flow of Col4 along the surface of the VNC is towards the head of the tissue, and driving a G552D temperature sensitive Col4 point mutant transgene specifically in hemocytes severely inhibits VNC condensation, related to Figures 3 and 4. (**A**) (Left panels) Col4 was live imaged during the 1st phase of VNC condensation simultaneously in the head and tail regions of the tissue. (Right panels) Tracking of Col4 motion by PIV in head vs. tail regions of the VNC. Scale bar = 10 μm (**B**) Quantification of the average Col4 speed for each frame reveals increased motion in the tail of the VNC when compared with the head. Mann-Whitney test. n = 127 frames for each sample. Each dot represents one frame. Boxplots show medians, 25th and 75th percentiles. ****p<0.0001.
(**C**) Correlation of the global alignment and orientation of PIV vectors for each frame reveals that the motion of the Col4 network is more coherent in the tail of the tissue and oriented towards the head (cosθ of 1 represents a tail-to-head orientation). Mann-Whitney test. n = 127 frames for each sample. Each dot represents one frame. Boxplots show medians, 25th and 75th percentiles. ****p<0.0001. **(D)** Kymographs of the average speed and the average x-component of the velocity of glial and Col4 motion during condensation (as in Figure S1A,B) from PIV analysis of lattice light-sheet imaging. Black line and white dotted line highlight the approximate start of condensation and end of the first phase, respectively. Note that the rapid wave of Col4 motion from tail to head slightly precedes movement of glia. **(E)** (top panel) Equidistant dorsoventral stripes were bleached across the Col4 network, which allowed for direct tracking of BM movement over time from the start of VNC condensation. (bottom panel) Linescan analysis through bleached regions (white shaded stripe) allowed for identification of the bleached fiducial marks over time (green and magenta lines highlight the trough of the bleached stripe at the start of the imaging and after 30 min, respectively). Note that there is progressively greater motion towards the tail of the tissue. Scale bar = 30 μm **(F)** (left panels) Cross sections of control and Col4 mutant (ΔCol4) VNCs at stage 15 (Stg15) and stage 17 (Stg 17) of development, which represent the 1st and 2nd condensation phases, respectively. (right panels) Green and magenta lines highlighting the shape of the VNC in control and Col4 mutant embryos at Stg 15 and Stg 17, respectively. Scale bar = 10 μm **(G)** Quantification of the eccentricity in VNC cross sections in control and Col4 mutants as analysed in (F). Note the decrease in eccentricity in Col4 mutants by stage 17 revealing a more circular shape. One sample t-test against the mean of Control (Stg 15). n = 3 cross-sections for each sample and stage. Each dot represents one cross-section. Boxplots show medians, 25th and 75th percentiles. **p=0.0093.

Supplementary Table

Table S1. Genotypes of the embryos used for each experiment. Related to STAR Methods.

Figure 1	A	*w; Mhc*^{1}*; Repo-Gal4, UAS-LifeActGFP, UAS-RedStinger /+*
	B-C	*w; Mhc*^{1}*; Repo-Gal4, UAS-LifeActGFP /+*
	D-E	*w; Col4α2-GFP; Repo-Gal4, UAS-LifeAct-mScarlet /+*
Figure 2	A	*"Control": w;; Repo-Gal4, UAS-LifeActGFP /+*
		"ΔCol4": w; Df(2L)BSC172; Repo-Gal4, UAS-LifeActGFP /+
	B-D	"Control": *w;; Repo-Gal4, UAS-LifeActGFP /+*
		"ΔPerlecan": *w; trol*^{null}*; Repo-Gal4, UAS-LifeActGFP /+*
		"ΔCol4": *w; Df(2L)BSC172; Repo-Gal4, UAS-LifeActGFP /+*
		"ΔLaminin": *w; Df(2L)LanB1; Repo-Gal4, UAS-LifeActGFP /+*
Figure 3	B-E	*w; Mhc*^{1}*, Col4α2-GFP; Repo-Gal4, UAS-LifeAct-mScarlet /+*
Figure 4	B	"Control": *w;* PS7-Gal4 /+; faxGFP /+
		"PS7>RacDN": *w; PS7-Gal4 /+; faxGFP / UAS-RacN*^{17}
		"PS7>MMP2": *w; PS7-Gal4 / UAS-MMP2; faxGFP /+*
	C	"Control": *w;; Sn-Gal4, UAS-Col4α2-GFP*
		"G552D": *w; Col4α1*^{G552D}*; Sn-Gal4, UAS-Col4α2-GFP*
	D	"G552D": *w; Col4α1*^{G552D}*; Sn-Gal4, UAS-Col4α2-GFP*
	E	"Control": *w;; repo-Gal4, UAS-LifeActGFP /+*
		"G552D": *w; Col4α1*^{G552D}*; Repo-Gal4, UAS-LifeActGFP /+*
	F	"Hemocytes>wt Col4α1":<br>*w; Mhc*^{1}*, Col4α2-GFP; Sn-Gal4, UAS- Col4α1*^{wt}*-mScarlet*
		"Hemocytes>G552D Col4α1":<br>*w; Mhc*^{1}*, Col4α2-GFP; Sn-Gal4, UAS- Col4α1*^{G552D}*-mScarlet*
	G	"Hemocytes>wt Col4α1":<br>*w; elav-mYFP; Sn-Gal4, UAS-Col4α1*^{wt}*-mScarlet*
		"Hemocytes>G552D Col4α1":<br>*w; elav-mYFP; Sn-Gal4,UAS-Col4α1*^{G552D}*-mScarlet*
		"Hemocytes>RacDN":<br>*w; elav-mYFP; Sn-Gal4,UAS-Col4α1*^{wt}*-mScarlet / UAS-RacN*^{17}
		"Hemocytes>RacDN; G552D Col4α1":<br>*w; elav-mYFP; Sn-Gal4, UAS- Col4α1*^{G552D}*-mScarlet / UAS-RacN*^{17}
	J	"Hemocyte>wt Col4α1":<br>*w; Mhc*^{1}*, Col4α2-GFP; Sn-Gal4,UAS- Col4α1*^{wt}*-mScarlet*
		"Hemocyte>Δ7S Col4α1":<br>*w; Mhc*^{1}*, Col4α2-GFP; Sn-Gal4, UAS- Col4α1*^{Δ7S}*-mScarlet*
	K	"Hemocyte>wt Col4α1":<br>*w; elav-mYFP; Sn-Gal4, UAS-Col4α1*^{wt}*-mScarlet*
		"Hemocyte>Δ7S Col4α1":<br>*w; elav-mYFP; Sn-Gal4,UAS-Col4α1*^{Δ7S}*-mScarlet*

Supplementary Figure 1	A-B	*w; Mhc^{1}; Repo-Gal4, UAS-LifeActGFP, UAS-RedStinger /+*
Supplementary Figure 1	C	"Control": *w;; Repo-Gal4, UAS-LifeActGFP /+*
Supplementary Figure 1	C	"Glia>RacDN": *w;; Repo-Gal4, UAS-LifeActGFP / UAS-RacN^{17}*
Supplementary Figure 1	D	"Control":<br>*w;; Repo-Gal4, UAS-LifeActGFP /+*
Supplementary Figure 1	D	"Glia>Myosin II DN":<br>*w; UAS-Zipper DN-GFP /+; Repo-Gal4, UAS-LifeActGFP /+*
Supplementary Figure 1	D	"Glia>Myosin II RNAi":<br>*w; UAS-Zipper RNAi /+; Repo-Gal4,UAS-LifeActGFP /+*
Supplementary Figure 1	E-F	*w;; Repo-Gal4, UAS-LifeActGFP, UAS-RedStinger / UAS-RacN^{17}*
Supplementary Figure 1	G	"Control":<br>*Elav-Gal4 /+;;Repo-mCD8::Cherry /+*
Supplementary Figure 1	G	"Neuron>RacDN":<br>*Elav-Gal4 /+;;Repo-mCD8::Cherry / UAS-RacN^{17}*
Supplementary Figure 1	H	"Control":<br>*Elav-Gal4 /+;;Repo-mCD8::Cherry /+*
Supplementary Figure 1	H	"Neuron>Myosin II DN":<br>*Elav-Gal4 /+; UAS-Myosin II DN /+; Repo-mCD8::Cherry /+*
Supplementary Figure 1	I-L	*Mhc^{1}; w;; Repo-Gal4, UAS-LifeActGFP, UAS-RedStinger /+*
Supplementary Figure 2	A-B	*w; Mhc^{1}, Col4α2-GFP*
Supplementary Figure 2	C	"Control": *w; Col4α2-GFP; Repo-Gal4, UAS-LifeAct-mScarlet /+*
Supplementary Figure 2	C	"Glia>MMP2": *w; Col4α2-GFP, UAS-MMP2 / Col4α2-GFP;<br>Repo-Gal4, UAS-LifeAct-mScarlet /+*
Supplementary Figure 2	D	"Control":<br>*w; Repo-Gal4, UAS-LifeAct-mScarlet /+*
Supplementary Figure 2	D	"Glia>MMP2":<br>*w; UAS-MMP2 /+; Repo-Gal4, UAS-LifeAct-mScarlet /+*
Supplementary Figure 2	D	"ΔCol4":<br>*w; Df(2L)BSC172; Repo-Gal4, UAS-LifeActGFP /+*
Supplementary Figure 3	A-D	"Control":<br>*w; elav-mYFP; Sn-Gal4, UAS- Col4α1^{wt}-mScarlet*
Supplementary Figure 3	A-D	"ΔHemocytes":<br>*w; Srp^{AS}; elav-mYFP*
Supplementary Figure 3	A-D	"Hemocytes>RacDN":<br>*w; elav-mYFP; Sn-Gal4, UAS-Col4α1^{wt}-mScarlet/ UAS-RacN^{17}*
Supplementary Figure 4	A-E	*w; Mhc^{1}, Col4α2-GFP; Repo-Gal4, UAS-LifeAct-mScarlet /+*
Supplementary Figure 4	F-G	"Control":<br>*w; Mhc^{1}, Col4α2-GFP; Repo-Gal4, UAS-LifeAct-mScarlet /+*
Supplementary Figure 4	F-G	"ΔCol4":<br>*w; Df(2L)BSC172 / Cg25C^{k13420}; Repo-Gal4, UAS-LifeActGFP /+*

Movie 1	Part 1-2	*w; Col4α2-GFP; Repo-Gal4, UAS-LifeAct-mScarlet /+*
	Part 3-4	*w; Mhc*^{1}*; Repo-Gal4, UAS-LifeActGFP, UAS-RedStinger /+*
	Part 5-6	*w; Col4α2-GFP; Repo-Gal4, UAS-LifeAct-mScarlet /+*
	Part 7	*w; elav-mYFP; Sn-Gal4, UAS-RedStinger*
Movie 2	Part 1	"ΔLaminin":<br>*w; Df(2L)LanB1; Repo-Gal4, UAS-LifeActGFP /+*
		"ΔPerlecan":<br>*w; trol*^{null}*; Repo-Gal4, UAS-LifeActGFP /+*
		"ΔCol4":<br>*w; Df(2L)BSC172 / Cg25C*^{k13420}*; Repo-Gal4, UAS-LifeActGFP /+*
	Part 2	"Control":<br>*w; elav-mYFP; Sn-Gal4, UAS- Col4α1*^{wt}*-mScarlet*
		"ΔHemocytes":<br>*w; Srp*^{AS}*; elav-mYFP*
		"Hemocytes>RacDN":<br>*w; elav-mYFP; Sn-Gal4, UAS-Col4α1*^{wt}*-mScarlet / UAS-RacN*^{17}
	Part 3	"Control": *w; elav-mYFP; Sn-Gal4, UAS-Col4α1*^{wt}*-mScarlet*
		"Hemocytes>RacDN":<br>*w; elav-mYFP; Sn-Gal4, UAS-Col4α1*^{wt}*-mScarlet / UAS-RacN*^{17}
		"Hemocytes>RacDN":<br>*w; elav-mYFP; Sn-Gal4, UAS-Col4α1*^{G552D}*-mScarlet/ UAS-RacN*^{17}
Movie 3	Part 1-3	*w; Mhc*^{1}*, Col4α2-GFP; Repo-Gal4, UAS-LifeAct-mScarlet /+*

Supplementary Methods

Methods S1. Supplementary methods for FE modelling. Related to STAR Methods.

1 Introduction

In this document, we illustrate in further detail the Finite Element (FE) model simulations of ventral nerve cord (VNC) condensation presented in the main text. Information on how the model was constructed, on how simulations were run and on parameters choice are given.

VNC morphogenesis involves two distinct phases, a first rapid isovolumetric phase lasting about 3 hours which shows an anisotropic 25% reduction in the length of the tissue from tail to head, and a second slower phase which shows a more isotropic condensation and a reduction in volume. As most of the morphological changes happen in the first phase of condensation, the modelling approach presented here focuses on this part of the process, with the aim of explaining the loading conditions necessary to initiate the anisotropic motion.

All FE simulations were performed in Abaqus (Simulia, Dassault Systèmes) by using static modelling. An idealised geometry was employed with dimensions acquired through experimental observations (Figure 1C in the main text) and different loading conditions were simulated.

2 FE model

An idealised shape representing the approximate VNC morphology was designed as an elliptical cylinder (Fig. S5). The head side of the VNC is physically connected to the embryonic brain, while the tail of the tissue is free to move and has a semi-spherical end. Relevant dimensions were chosen according to experimental observations (Figure 1C in the main text), with the elliptical cylinder measuring 400 μm in length, and displaying a cross section of major and minor axes measuring 83 μm and 38 μm, respectively.

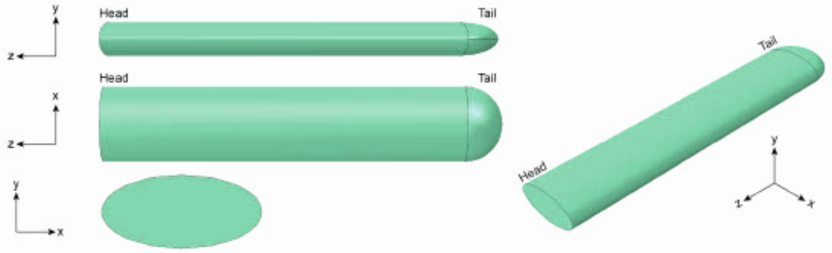

Fig. S5: Geometry of the FE model, related to Figure 3. Orthogonal views of the FE model of the VNC. The model is constituted by an elliptical cylindrical shape which dimensions were selected to match the experimental observations. The head side is connected to the tissue forming the brain, while the tail end is free to move.

The model was constituted by two materials, a tissue core which represents glia and neurons, and a surrounding thin shell representing the basement membrane. This surrounding layer was modelled as a 0.1 μm-thick skin shell. Both materials were defined as linear elastic with Poisson ratio of 0.48. Default Young's moduli were set to 70 Pa for the tissue and 30 Pa for the basement membrane, if not otherwise specified in certain simulation cases. These values were chosen to match the experimental observations performed by atomic force microscopy (Figure 2A in the main text). A shrinking coefficient was assigned to the shell layer when simulating surface tension.

To perform FE analysis, the geometry has to be meshed into a finite number of elements of known dimension. The geometry was therefore meshed with a total of 79818 nodes and 61689 elements for analysis, corresponding to elements of about 5 μm in size (Fig. S6). Two different types of elements were used: 7436 linear triangular elements for the shell (type S3) and 54253 quadratic tetrahedral elements for the tissue (type C3D10). Convergence tests were run for a range of different element dimensions (from 2.5 to 20 μm), each analysis showed little variation in the simulated reduction in length when loading the model with uniform pressure.

The model was constrained at the head side, where the VNC is attached to the brain tissue in the *Drosophila* embryo. As the brain tissue is of considerable dimensions compared to the VNC, it was hypothesised that this link could be simulated with an encastre, constraining all translational and rotational degrees of motion (Fig. S7).

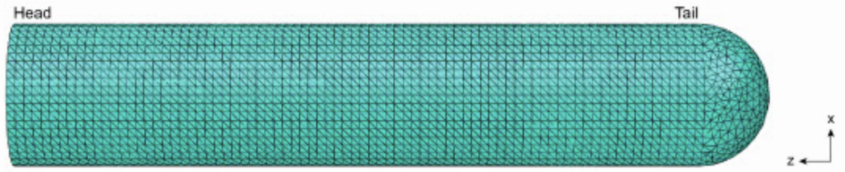

Fig. S6: Mesh of the FE model, related to Figure 3. Top-down view of the FE model showing the mesh used, with elements of about 5 μm in size. Linear triangular elements or quadratic tetrahedral elements were employed for the shell and tissue, respectively.

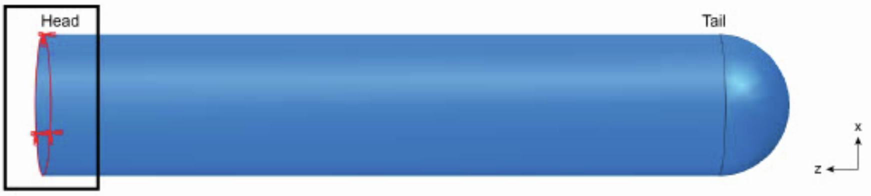

Fig. S7: Boundary conditions for the FE model, related to Figure 3. The model was constrained by imposing an encastre at the head side (black square) simulating its attachment to the brain tissue in the *Drosophila* embryo.

3 Loading scenarios

Different loading scenarios were simulated with the goal of reproducing the experimentally observed change in shape of the VNC during the first phase of morphogenesis. This phase of the process is characterised by an anisotropic reduction in length from the tail to the head of the tissue. The first phase does not display a change in the volume of the VNC, and the cross-section of the tissue expands along both axes to compensate for the reduction in length (Figure 1C in the main text).

The different simulations performed and their rationales are outlined in the following sections. The simulated magnitude of the displacement along the length of the VNC was used as output, as a proxy for the experimentally observed motion of the tissue.

3.1 Uniform pressure

We first loaded the model with a uniform pressure, normal to each element. This would correspond to a cell-driven active compression applied on the tissue, as the cells would locally contract resulting in a perpendicular force on the VNC. This loading scenario caused a reduction in length from tail to head as experimentally observed; however, it was accompanied by a reduction in the size of both axes in the cross-section, suggesting a change in volume which diverged from experimental data (Fig. S8). Increasing the magnitude of the applied pressure resulted in a more marked shrinking in all axes (Fig. S9). To obtain a reduction in length of 25%, a uniform pressure of $0.38\ kPa$ was applied.

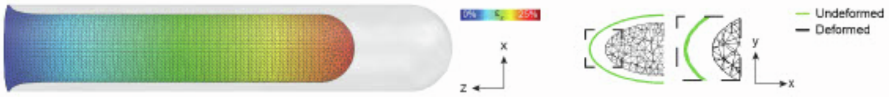

Fig. S8: Loading the FE model with a uniform pressure, related to Figure 3. Simulating loading with a perpendicular uniform pressure on the VNC resulted in its anisotropic reduction in length from tail to head (left panel). However, reduction in both the height and width of the cross-section was also observed (right panel), diverging from experimental evidence. Colours represent the magnitude of displacement along the length of the tissue (z-axis) with values ranging from 0% to 25% of strain.

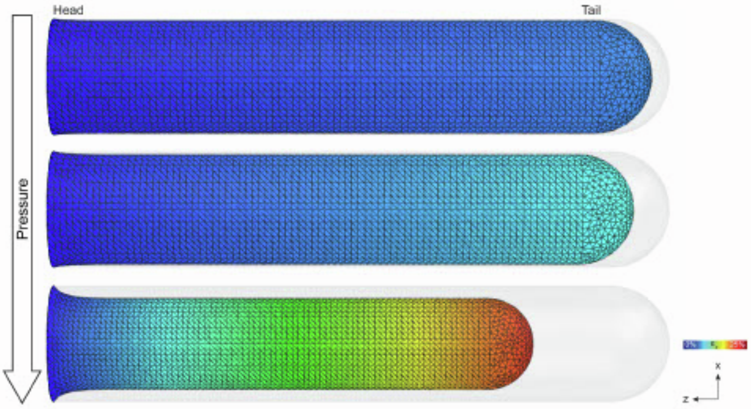

Fig. S9: Loading the FE model with a uniform pressure - effect of increasing the pressure magnitude, related to Figure 3. Increasing the magnitude of the simulated normal pressure exacerbated the amount of length reduction (top panel: pressure magnitude $0.05\ kPa$, middle: $0.1\ kPa$, bottom: $0.38\ kPa$). Colours represent the magnitude of displacement along the length of the tissue (z-axis) with values ranging from 0% to 25% of strain.

3.2 Isotropic surface tension

We then simulated a loading scenario involving surface tension, as we hypothesised a role for the basement membrane layer surrounding the tissue in driving its condensation. To this aim, we assigned a temperature-controlled expansion coefficient to the shell material: by simulating a drop in temperature, we could cause the material of the shell to shrink. Similarly to loading the model with uniform pressure, this simulation resulted in an anisotropic reduction in length of the VNC. When looking at the cross-section of the tissue, we observed the expected increase in size along the y-axis, but also a decrease along the x-axis, with a tendency towards a more circular shape, which did not match with the experimental observations (Fig. S10).

We could modulate the ability of the shell to shrink by keeping the temperature drop constant (i.e., 100 to 0 degrees) and subsequently changing the expansion coefficient (Fig. S11). This resulted in a progressively larger reduction in the VNC length. To simulate a 25% reduction in length, an expansion coefficient of 0.65 was required.

The magnitude of the surface tension could also be adjusted by changing the material properties of the shell. In fact, by increasing the stiffness of the shell, it was possible to increase the reduction in length of the tissue, while keeping constant both the temperature drop (100 to 0 degrees) and expansion coefficient (0.65).

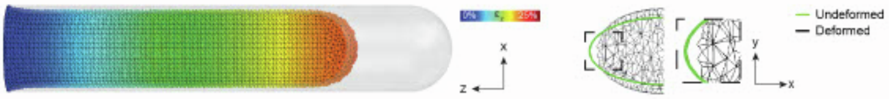

Fig. S10: Loading the FE model with an isotropic surface tension, related to Figure 3. Simulating surface tension by allowing the shell material to shrink resulted in an anisotropic reduction in length towards the head (left panel). The change in shape in the cross-section showed increased dimensions in height and decreased dimensions in width (right panel). Colours represent the magnitude of displacement along the length of the tissue (z-axis) with values ranging from 0% to 25% of strain.

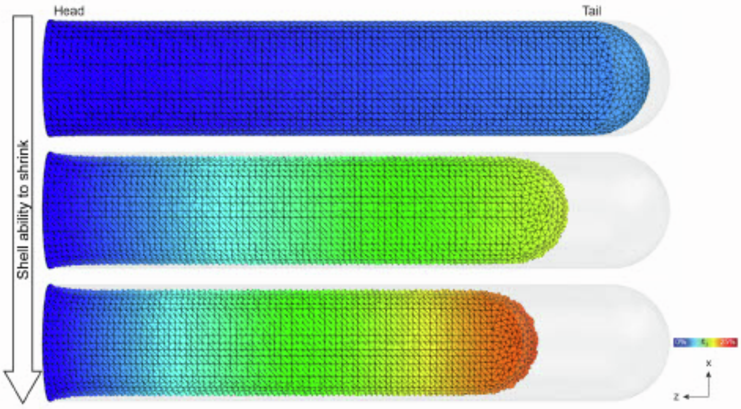

Fig. S11: Loading the FE model with an isotropic surface tension - effect of increasing the shell ability to shrink, related to Figure 3. Simulating surface tension by allowing the shell material to shrink resulted in an anisotropic reduction in length towards the head, proportional to the assigned expansion coefficient (top panel: expansion coefficient 0.1, middle: 0.5, bottom: 0.65). Colours represent the magnitude of displacement along the length of the tissue (z-axis) with values ranging from 0% to 25% of strain.

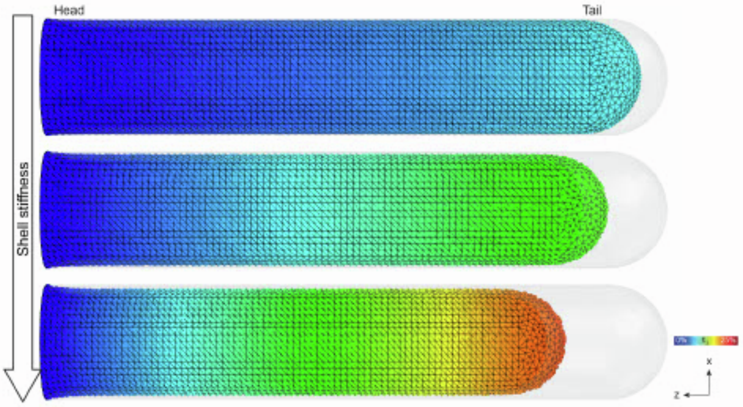

Fig. S12: Loading the FE model with an isotropic surface tension - effect of increasing the shell stiffness, related to Figure 3. Simulating surface tension by allowing the shell material to shrink resulted in an anisotropic reduction in length towards the head, proportional to the assigned shell stiffness (top panel: shell/tissue Young's moduli $10/90\ kPa$, middle: $20/80\ kPa$, bottom: $30/70\ kPa$). Colours represent the magnitude of displacement along the length of the tissue (z-axis) with values ranging from 0% to 25% of strain.

3.3 Longitudinal surface tension

Finally, we simulated a longitudinal surface tension along the length of the tissue (z-axis), as we hypothesised that the observed gradient of collagen from head to tail may lead to a preferential directionality of surface tension (Supplementary Figure 2AB). This was achieved by defining an anisotropic shell shrinking behaviour, which was described by an expansion coefficient for each of the three material orientations (Fig. S13). We set to zero the expansion coefficients for the normal and radial orientations, and only kept active the longitudinal component for each element (0.65), which prevented the tissue from shrinking in the normal and radial axes. This showed an anisotropic reduction in VNC length from tail to head, which correlated with an expansion in both axes of the cross-section (Fig. S14). This result was analogous to what was observed experimentally, suggesting that a longitudinal surface tension dictated by the collagen layer surrounding the tissue might be the driving force of the first phase of the condensation process.

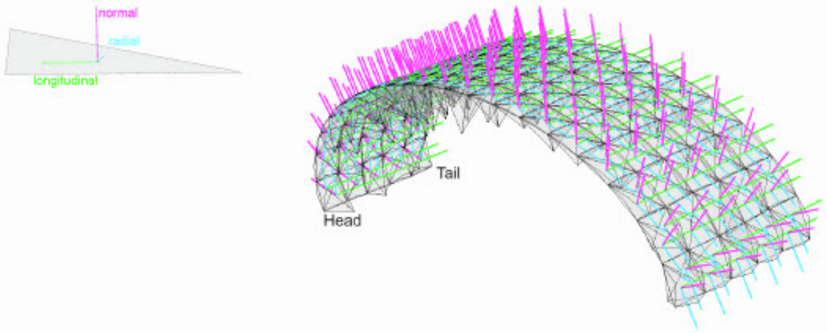

Fig. S13: Loading the FE model with an anisotropic surface tension - evaluation of material orientation, related to Figure 3. Simulating an anisotropic surface tension required assigning an expansion coefficient for each material orientation. Each element is assigned its own reference, with a normal (magenta), radial (cyan), and longitudinal (green) orientation.

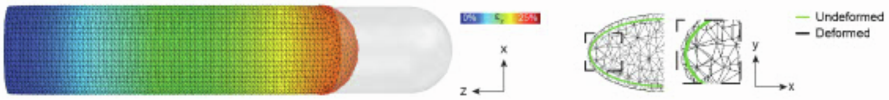

Fig. S14: Loading the FE model with an anisotropic surface tension, related to Figure 3. Simulating a longitudinal surface tension by allowing the shell material to shrink only along the length of the tissue resulted in an anisotropic reduction in length towards the head (left panel) and an increase in both height and width of the tissue (right panel). This result mimicked the experimental observations in the *Drosophila* embryo. Colours represent the magnitude of displacement along the length of the tissue (z-axis) with values ranging from 0% to 25% of strain.

3.4 Comparison

While all three simulations (uniform pressure, isotropic surface tension, and longitudinal surface tension) showed an anisotropic reduction in the length of the tissue from tail to head (Fig. S15), the results when looking at the tissue cross-section varied, with only the longitudinal surface tension scenario predicting the expansion in both height and width of the tissue which was observed experimentally in the *Drosophila* embryo (Fig. S14).

This difference is further highlighted by the vectorial representation of the displacement (Fig. S16): it can be noted how, in the case of the uniform pressure loading, the vectors point towards a central node at the head of the tissue

suggesting a deformation along all axes. For the isotropic surface tension, this behaviour is less pronounced, but can still be observed towards the head showing additional deformation along the cross-section of the tissue. Finally, in the longitudinal surface tension case, the vectors have a tendency to point outwards from the tissue centre which explains the expansion in both x- and y-axis. The FE simulations, therefore, suggested that VNC condensation can be explained by a preferentially directed surface tension.

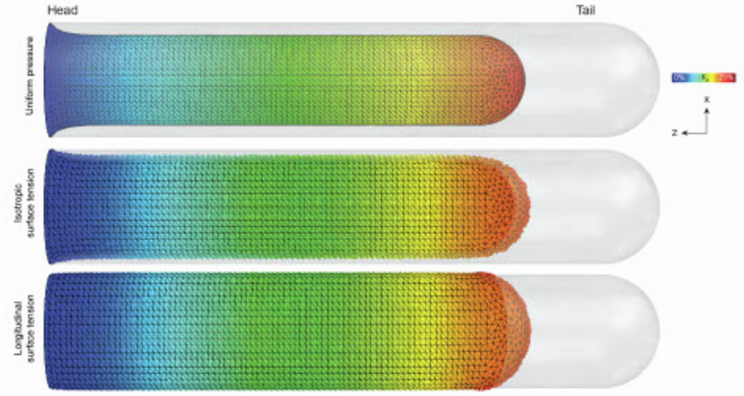

Fig. S15: Comparing the displacement along the length of the tissue in the different loading conditions, related to Figure 3. Loading the model by uniform pressure, isotropic, and longitudinal surface tension resulted in an anisotropic reduction in the VNC length from tail to head. Colours represent the magnitude of displacement along the length of the tissue (z-axis) with values ranging from 0% to 25% of strain.

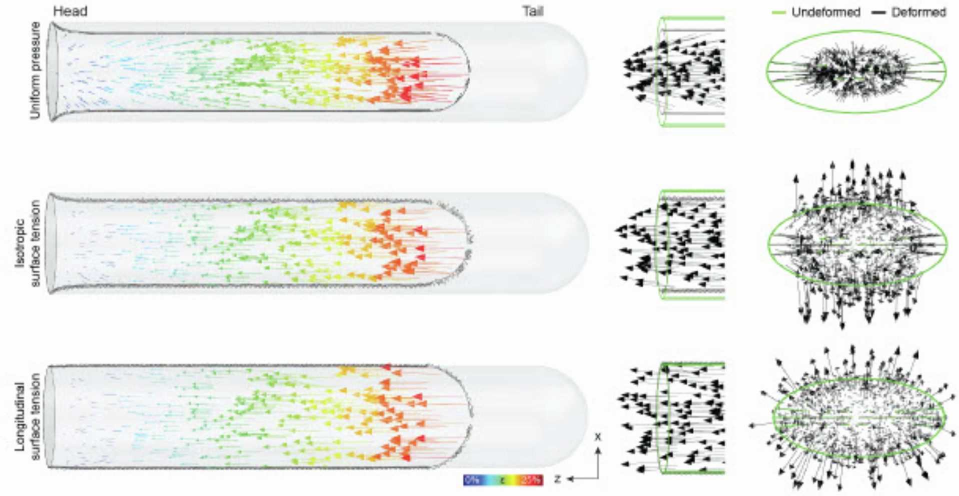

Fig. S16: Comparing the displacement vectors in the different loading conditions, related to Figure 3. Vectorial representation of the displacement of the VNC when comparing loading the model by uniform pressure, isotropic, and longitudinal surface tension (left panels). The representation with unit vectors on a vertical cut along the VNC length highlights the directionality of the displacement (right panels). Colours represent the magnitude of overall displacement with values ranging from 0% to 25% of strain.

4 Disrupting the longitudinal surface tension

We hypothesised that an increase in longitudinal surface tension would drive the tissue reduction in length by global transmission of ECM-generated stresses through the polymer network. We therefore tested the effects of a local disruption in the surface tension on VNC condensation.

To achieve this, we defined the initial temperature field controlling the shell shrinking as a function of the tissue length (z-axis), before turning the temperature to 0 degrees to enable the shell shrinking. If no disruption in surface tension was to be simulated, the temperature value was kept constant along z (top panel Fig. S17, and Fig. S14). Otherwise, we analytically defined the shape of the initial temperature field along z with a Gaussian-shaped drop in temperature around the centre of the VNC (middle and bottom panels Fig. S17). The drop could be modulated to simulate a partial or null surface tension in a stripe of the tissue, by setting the target temperature to 50% (middle panel Fig. S17) or 0% (bottom panel Fig. S17) of the temperature away from the stripe.

Simulations revealed that a local disruption of the surface tension had an effect on the reduction in length of the tissue, proportional to the severity of the disruption (Fig. S17). Similar results were obtained when experimentally causing a local disruption of the collagen network, by expressing surface-bound matrix metalloproteases in a stripe along the VNC (Figure 4B in the main text). These results taken together suggest that surface tension-driven change in tissue shape should involve long-range and coordinated morphological remodelling.

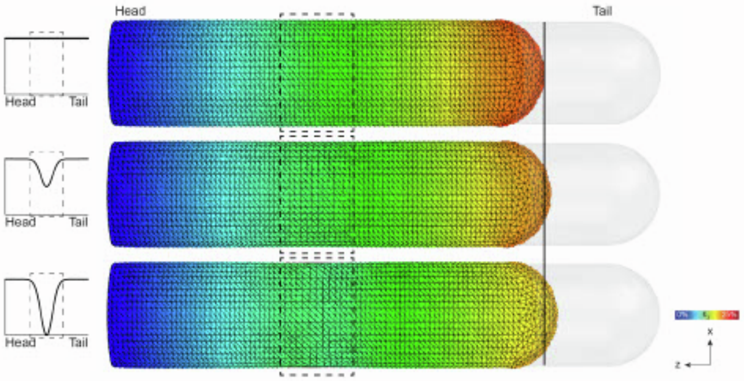

Fig. S17: Modelling the disruption of surface tension, related to Figure 4. The reduction in VNC length was affected by locally disrupting the surface tension (black vertical line). This was achieved by locally modifying the initial temperature field of the simulation with a drop around the middle of the tissue (black squares) of either 50% (middle panel) or 100% (bottom panel), corresponding to halving or deleting the surface tension in a stripe of the VNC, respectively. Colours represent the magnitude of displacement along the length of the tissue (z-axis) with values ranging from 0% to 25% of strain.
